# Epidemiological Trends and Attributable Risk Burden of Cervical Cancer: An Observational Study from 1990 to 2019

**DOI:** 10.1155/2022/3356431

**Published:** 2022-09-30

**Authors:** Hu Yao, Chen Yan, He Qiumin, Zhou Li, Ai Jiao, Li Xin, Li Hong

**Affiliations:** ^1^Department of Obstetrics and Gynecology, Renmin Hospital of Wuhan University, Wuhan, Hubei 430060, China; ^2^Department of Obstetrics and Gynecology, Jingzhou Central Hospital, The Second Clinical Medical College, Yangtze University, Jingzhou, Hubei 434020, China; ^3^Department of Urinary Surgery, Jingzhou Central Hospital, The Second Clinical Medical College, Yangtze University, Jingzhou, Hubei 434020, China

## Abstract

**Background:**

Cervical cancer, especially in underdeveloped areas, poses a great threat to human health. In view of this, we stratified the age and social demographic index (SDI) based on the epidemiological development trend and attributable risk of cervical cancer in countries and regions around the world.

**Methods:**

According to the data statistics of the global burden of disease database (GBD) in the past 30 years, we adopted the annual percentage change (EAPCs) to evaluate the incidence trend of cervical cancer, that is, incidence rate, mortality, and disability adjusted life expectancy (DALY). Meanwhile, we investigated the potential influence of SDI on cervical cancer's epidemiological trends and relevant risk factors for cervical cancer-related mortality.

**Results:**

In terms of incidence rate and mortality, the high SDI areas were significantly lower than those of low SDI areas. The incidence and mortality in women aged 20 to 39 were relatively stable, whereas an upward trend existed in patients aged 40 to 59. The global cervical cancer incidence rate increased from 335642 in 1990 to 565541 in 2019 (an increase of 68.50%, with an average annual growth rate of 2.28%), while the age-standardized incidence rate (ASIR) showed a slight downward trend of 14.91/100000 people (95% uncertainty interval [UI], 13.37-17.55) in 1990 to 13.35/100,000 persons (95% UI, 11.37-15.03) in 2019. The number of annual deaths at a global level increased constantly and there were 184,527 (95% UI, 164,836-218,942) deaths in 1990 and 280,479 (95% UI, 238,864-313,930) deaths in 2019, with an increase of 52.00%(average annual growth rate: 1.73%). The annual age-standardized disability adjusted annual life rate showed a downward trend (decline range: 0.95%, 95% confidence interval [CI], from −1.00% to − 0.89%). In addition, smoking and unsafe sex were the main attributable hazard factors in most GBD regions.

**Conclusions:**

In the past three decades, the increase in the global burden of cervical cancer is mainly concentrated in underdeveloped regions (concentrated in low SDI). On the contrary, in countries with high sustainable development index, the burden of cervical cancer tends to be reduced. Alarmingly, ASIR in areas with low SDI is on the rise, which suggests that policy makers should pay attention to the allocation of public health resources and focus on the prevention and treatment of cervical cancer in underdeveloped areas, so as to reduce its incidence rate, mortality, and prognosis.

## 1. Introduction

Worldwide, cervical cancer, as the fourth most common malignant tumor, still has a high mortality rate among women [1–3]. As a major global health concern, the predominance of cervical cancer has been significantly ascended in underdeveloped regions [4]. In view of this, the United Nations General Assembly clearly proposed in the sustainable development strategy to strive to reduce the premature mortality of noncommunicable diseases by at least 30% by the middle of this century [5]. Meanwhile, the World Health Organization (WHO) has launched a global initiative to expand prevention, screening, and strive to eliminate the public health problem of cervical cancer in the 21st century [6]. Considering the sustainable development goals, an irreplaceable goal is to understand the level and trend of cervical cancer burden to guide national and regional health policies, so as to improve the quality of life of cancer patients and prolong their life.

Up to now, there is still a lack of systematic evaluation of cervical cancer disease surveillance and investigation, health administration report, major disease registration, and all available data sources [7, 8]. Fortunately, the global burden of disease (GBD) collects disease data (including cervical cancer) from most countries and regions around the world. From a macro level, it provides an excellent opportunity to explore the epidemiological development trend of cervical cancer and the attributable risk [9, 10]. To date, the GBD database continuously provides available data on disease, injury, and risk factor burden by integrating global, regional, and national assessments [9, 11]. Thus far, epidemiological studies may draw more reliable conclusions from the GBD database [12].

With this in mind, we conducted this study to find attributive risk factors based on the incidence rate, mortality, DALY, and prevalence trends of cervical cancer in 192 countries and regions worldwide recorded based on the GBD database over the past 30 years and stratified them by age and social demographic index (SDI). The results of this study will help to analyze the regional differences in the global burden of cervical cancer and can formulate targeted health policies to optimize the allocation of medical resources for cervical cancer patients in underdeveloped areas.

## 2. Methods

### 2.1. Data Acquisition

In this study, the data of cervical cancer disease burden were obtained from the online Global Health Data Exchange (GHDx) query tool (http://ghdx.healthdata.org/gbd-results-tool). To further grade the disease burden of cervical cancer, the social population index (SDI) can be used as the optimal quantitative standard, that is, according to the SDI index (a total of five grades, including higher, high-middle, middle, low-middle and low), that is, all countries and regions included in the SDI can be classified into the corresponding SDI grade. Additionally, we also map and visualize the epidemiological trends (incidence rate, mortality, and annual disability adjusted life rate) of cervical cancer in 192 countries and regions where relevant data can be obtained, so as to clarify the regional distribution of the annual standardized incidence, death, and disability adjustment of cervical cancer, as well as the corresponding trends in different countries and regions in the past 30 years. Our research strictly respected the implementation of the declaration of Helsinki and complied with relevant regulations. It was approved by the Institutional Review of the Ethics Committee of Renmin Hospital of Wuhan University (WDRY2021-K014) to carry out this research.

### 2.2. Statistical Analysis

To meet the criteria of GBD data survey, we conducted statistical analysis on the incidence trend and death trend of cervical cancer, as well as age-standardized incidence rate (ASIR) and age-standardized mortality (ASDR). In addition, in order to evaluate the health life-years lost by cervical cancer patients due to illness, we used the annual disability adjusted life rate (DALY) analysis. It is worth mentioning that because the incidence trend of cervical cancer in the past 30 years has not continued to increase or decrease, we also adopted the annual percentage change (EAPCs) to accurately evaluate the epidemiological trend of cervical cancer incidence rate, mortality, and disability adjusted life age [13]. EAPCs were calculated using a linear regression model as follows: ln (ASR) = *α* + *β x* + *ε*, where *x* refers to the calendar year, and the ASR was obtained as follows [14]:

(1) $$\begin{array}{c} \text{ASR}=\frac{\sum_{i=1}^{A}\text{aiwi}}{\sum_{i=1}^{A}\text{wi}}\times 100,000. \end{array}$$

In the *i*th age subgroup, *a*_*i*_ is represented as age class. w_*i*_ denotes the number of persons (or weight), where *i* is equal to the selected reference standard population. Meanwhile, the EAPC = 100 × (exp(*β*) − 1) and 95% uncertainty intervals (UIs) were calculated via the regression model. If the lower limit and confidence interval of EAPC are greater than zero, ASR represents that the incidence or death of cervical cancer is on the rise, and vice versa [15, 16]. In addition, the human development index can be used as a quantitative standard for the evaluation of medical level in all regions of the world. We also used a scatter diagram for visualization to depict the correlation between EAPC, ASR, and SDI, in which the Pearson's correlation coefficient(*R*) represents the strength of the correlation. The data statistics and visual analysis involved in this study are based on *R* language (version 4.0.4, http://www.r-project.org/).

## 3. Results

### 3.1. Temporal and Spatial Changes of Incidence Rate of Cervical Cancer

#### 3.1.1. Global Level

**Figure 1:**
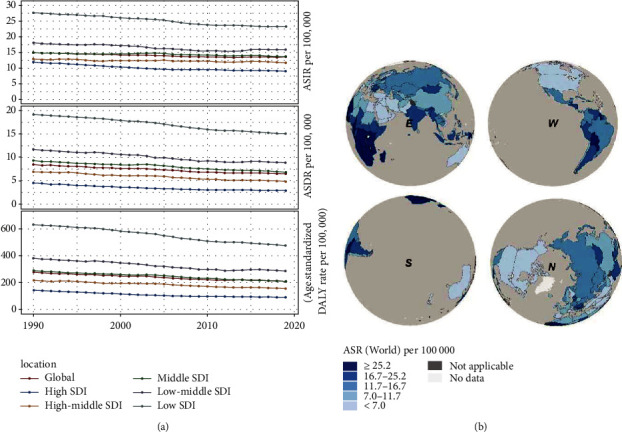
Trends in age-standardized incidence rate, mortality rate, and DALY rate among different SDI quantiles (a) and estimated age-standardized incidence rates on a global scale (b).

Compared with the cutoff point in 2019, the incidence rate of cervical cancer in the past 1990 was higher. There were 335,642 (95% UI, 300,354-393,893) incidences in 1990 and 565,541 (95% UI, 481,524-636,435) incidences in 2019 (Table 1, Additional file 1: Figure S1). The total incidence rate of the disease growth was 68.50%, and the average annual growth rate was 2.28%. On the contrary, the ASIR was relatively stable and shows a slight downward trend, with 14.91/100,000 persons (95% UI, 13.37-17.55) in 1990 to 13.35/100,000 persons (95% UI, 11.37-15.03) in 2019. Of note, the downward trend of ASIR was highly consistent with ASDR and DALY, and the downward trend of ASDR and DALY was slightly gentle compared with the ASIR (Figure 1).

#### 3.1.2. SDI Level

**Figure 2:**
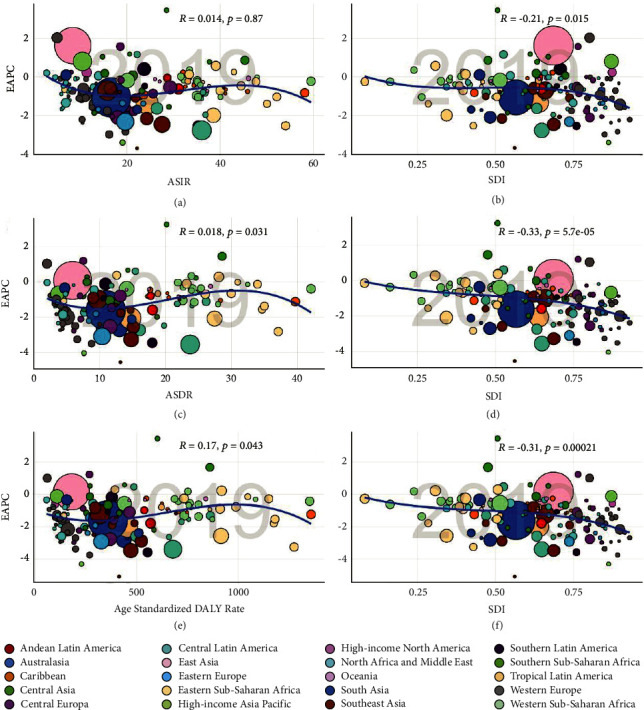
Correlation between EAPC and cervical cancer ASR (incidence (a), death (c), and DALY (e)) in 1990 and SDI (incidence (b), death (d), and DALY (f)) in 2019. The circle represents the country where the SDI data can be traced. The size of the circle represents the proportion of patients with cervical cancer. R index and *P* value were obtained by Pearson correlation analysis. ASR, age-standardized incidence/death/DALY rate; EAPC, estimated annual percentage change; SDI, social demographic index.

**Figure 3:**
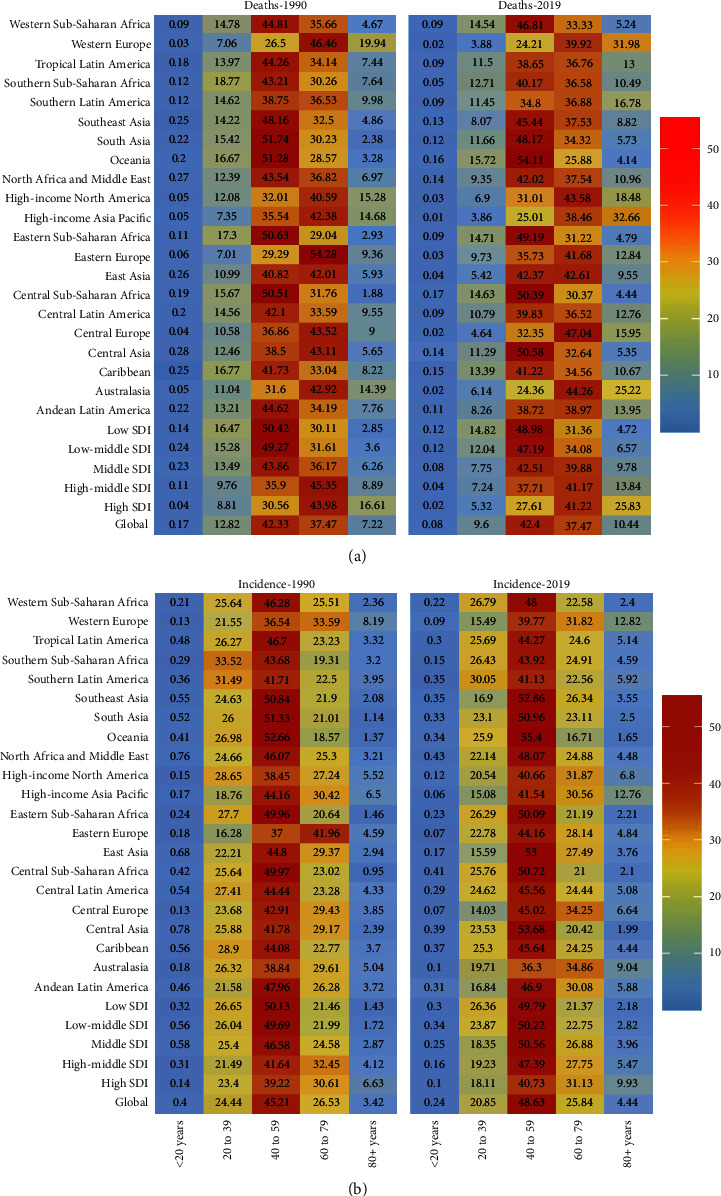
Incidence rate of cervical cancer and the distribution of death cases in different ages and regions. (a) The death rate from 1990 to 2019. (b) Incidence from 1990 to 2019.

As shown in Table 1, ASIR decreased in high SDI areas, with EAPCs of −0.95 (95% CI, from −1.06 to −0.85), followed by low SDI, with EAPCs of −0.69 (95% CI, from −0.73 to −0.65). The ASIR in other SDI regions maintained a basically flat development trend, while EAPC showed a negative correlation with different SDIs (*R* = −0.21, *P* < 0.05, Figure2(b)), but the correlation with ASIR was not obvious (*R* = 0.01, *P*=0.87, Figure 2(a)). In terms of incidence rate and age distribution, SDI was negatively correlated with the proportion of cases (especially young patients) (low SDI: 20-39 years old, 26.26%; medium and low SDI: 20-39 years old, 23.87%), while the proportion of elderly cases in 2019 (low SDI: 40-59 years old, 49.79/100000; medium and low SDI: 40-59 years old, 50.22/100000) remained relatively stable. However, the incidence of high incidence rate in 5 SDI regions was concentrated in 40 to 59 years (40.73/100,000 to 50.56/100,000). In the past 30 years, the incidence trend of the elderly population (60-79 years old) has remained relatively stable, with 2001 as the turning point, while the incidence rate of middle-aged and elderly women (40-59 years old) has increased slightly(Figure 3, Additional file1: Figure S2, Additional file1: Table S6).

### 3.2. National and Regional Levels

**Figure 4:**
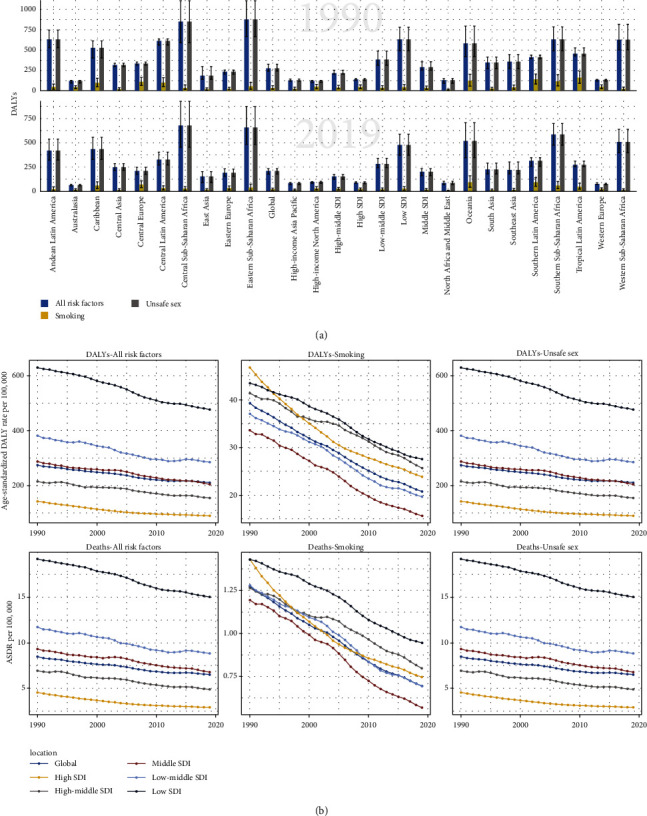
Cervical cancer DALY (a), age-standardized rates of DALY, and ASDR (b) attributable to risk factors compared in 1990 and 2019. DALY, disability-adjusted life-years; ASDR, age-standardized death rate.

We observed the incidence from GBD regions and country level via ASIR, a total of 40 countries showed an uptrend of ASIR from 1990 to 2019. Among them, Lesotho (1990 : 28.52/100,000, 95% UI [18.78-42.2]; 2019 : 52.77/100,000, 95% UI [26.49-90.4]) had the most obvious upward trend, followed by Botswana (1990 : 37.72/100,000, 95% UI [24.27-57.1]; 2019 : 47.63/100,000, 95% UI [28.09-73.79]). On the contrary, Rwanda (1990 : 54.02/100,1000,95% UI [37.19-73.18]; 2019 : 32.39/100,000, 95% UI [21.4-48.8]) represented the most obvious downward trend (Additional file1: Table S3). The top three countries in ASIR were Solomon Islands, Guinea, and Lesotho, respectively. The bottom three countries were Egypt, the Syrian Arab Republic, and Kuwait, respectively. Meanwhile, the top three and bottom three countries of EAPC were Lesotho, Italy, China, Maldives, Singapore, and Austria respectively. In terms of region, the top and bottom three regions of ASIR were sub-Saharan Africa (southern, central, and eastern), North Africa, the Middle East, Australia, and Western Europe. Furthermore, the top three and bottom three regions of EAPC were East Asia, southern sub-Saharan Africa, Eastern Europe, Latin America, Tropical Latin America, and South Asia, respectively (Additional file1: Tables S1 and S2.  Figure4 and Additional file1: Figure S6).

### 3.3. Temporal and Spatial Distributions of Death Trend of Cervical Cancer

#### 3.3.1. Global Level

Over the past three decades, the number of annual deaths at a global level increased constantly and there were 184,527 (95% UI, 164,836-218,942) deaths in 1990 and 280,479 (95% UI, 238,864-313,930) deaths in 2019 (Table 2). The total death rate of the disease growth was 52.00%, and the average annual growth rate was 1.73%. However, the downward trend of ASDR was very obvious, that is, EAPC is −0.93[95% CI, from −0.98 to −0.88], from 8.48/100000 people [95% UI, 7.59-10.07] in 1990 to 6.51/100000 people [95% UI, 5.55-7.29] in 2019.

#### 3.3.2. SDI Level

**Figure 5:**
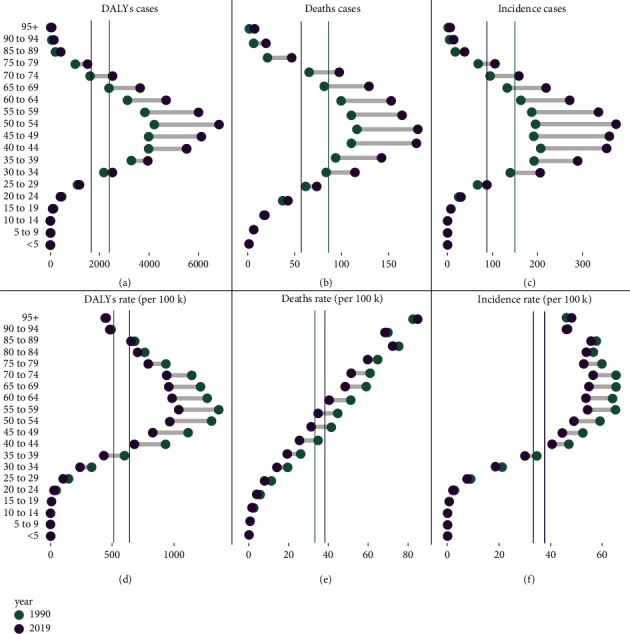
Incidence, death, and DALY rates of cervical cancer in different age groups.

Meanwhile, ASDR with low SDI had a significantly higher trend compared than the other four SDI regions, while ASDR with high SDI ranks lower. In addition, the ASDR of SDI regions showed a downward trend regardless of grade. In addition, EAPC showed a significant negative correlation with SDI (*R* = −0.33, *P* < 0.01, Figure 2(d)), while it showed a positive correlation with ASDR (*R* = 0.18, *P* < 0.05, Figure 2(c), Additional file1: Figure S3). Consistent with the SDI, the proportion of middle-aged deaths (40 to 59 years old) decreased with SDI in 1990 and 2019 (Figure 3). Meanwhile, the proportion of death among young patients (20-39 years old) had a downward trend year by year, while the proportion of death among the elderly (40-59 years old) had the opposite trend (Additional file1: Figure S4 and S5). Among them, there was a peculiar unimodal distribution (trend of death difference) among all age groups. The peak of low SDI was 60 to 64 years old, the peak of high medium, low medium, and medium SDI was 70 to 74 years old, and the peak of high SDI was 75 to 79 years old (Figure 5, Additional file1: Figure S5).

### 3.4. National and Regional Levels

From the changing trend of ASDR in various countries and regions around the world, most countries and regions had a downward trend. Among them, Rwanda (1990 : 37.11/100,000, 95% UI [25.71-49.62]; 2019 : 20.62/100,000, 95% UI [14.10-29.74]) had the most obvious downward trend, followed by Mexico (1990 : 23.68/100,000, 95% UI [18.62-24.98]; 2019 : 9.53/100,000, 95% UI [7.68-12.64]). On the contrary, Lesotho (1990 : 20.16/100,1000,95% UI [13.48-29.14]; 2019 : 35.96/100,000, 95% UI [18.42-60.81]) represented the most obvious upward trend. At the national level, the top three and bottom three countries in ASDR were Guinea, Lesotho, Zimbabwe, Kuwait, Egypt, and Finland, respectively (Additional file1: Table S4; Additional file1: Figure S6).

### 3.5. The Change in DALY of Cervical Cancer

#### 3.5.1. Global Level

At the global level, it was encouraging that DALYs have continued to rise. As shown in Table 3 and Additional file1: Table S5, there were 617,625 (95% UI, 543,767-731,693) DALY in 1990 and 895,501 (95% UI, 754,773-997,846) DALY in 2019. The total DALY rate of the disease growth was 44.99%, and the average annual growth rate was 1.50%. Consistent with the trend of DALY, the age-standardized DALY rate decreased significantly, that is, with the EAPC of −0.95[95% CI, from −1.00 to −0.89], from 275.05/100000 people [95% UI, 242.75-326.15] in 1990 to 210.64/100000 people [95% UI, 177.67-234.85] in 2019.

#### 3.5.2. SDI Level

From the level of sustainable development index, the age-standardized disability adjusted annual life rate in the five regions with sustainable development index showed a downward trend, especially in the regions with low sustainable development index, there was an obvious downward trend at the two time nodes (1990 : 630.59/100,000, 95% UI [487.61-777.41]; 2019 : 477.53/100,000, 95% UI [374.33-591.38]). Additionally, we found that EAPC and age-standardized DALY rate and SDI showed positive correlation (*R* = 0.17, *P* < 0.05, Figure 2(e)) and negative correlation (*R* = −0.31, *P* < 0.01, Figure 2(f)), respectively. The age distribution of DALY had the characteristics of “unimodal trend,” and the mortality distribution in various SDI regions also showed amazing similarity (aged 55-59 and aged 60-64) (Additional file1: Figure S5; Additional file1: Tables S7 and S8).

### 3.6. National and Regional Levels

**Figure 6:**
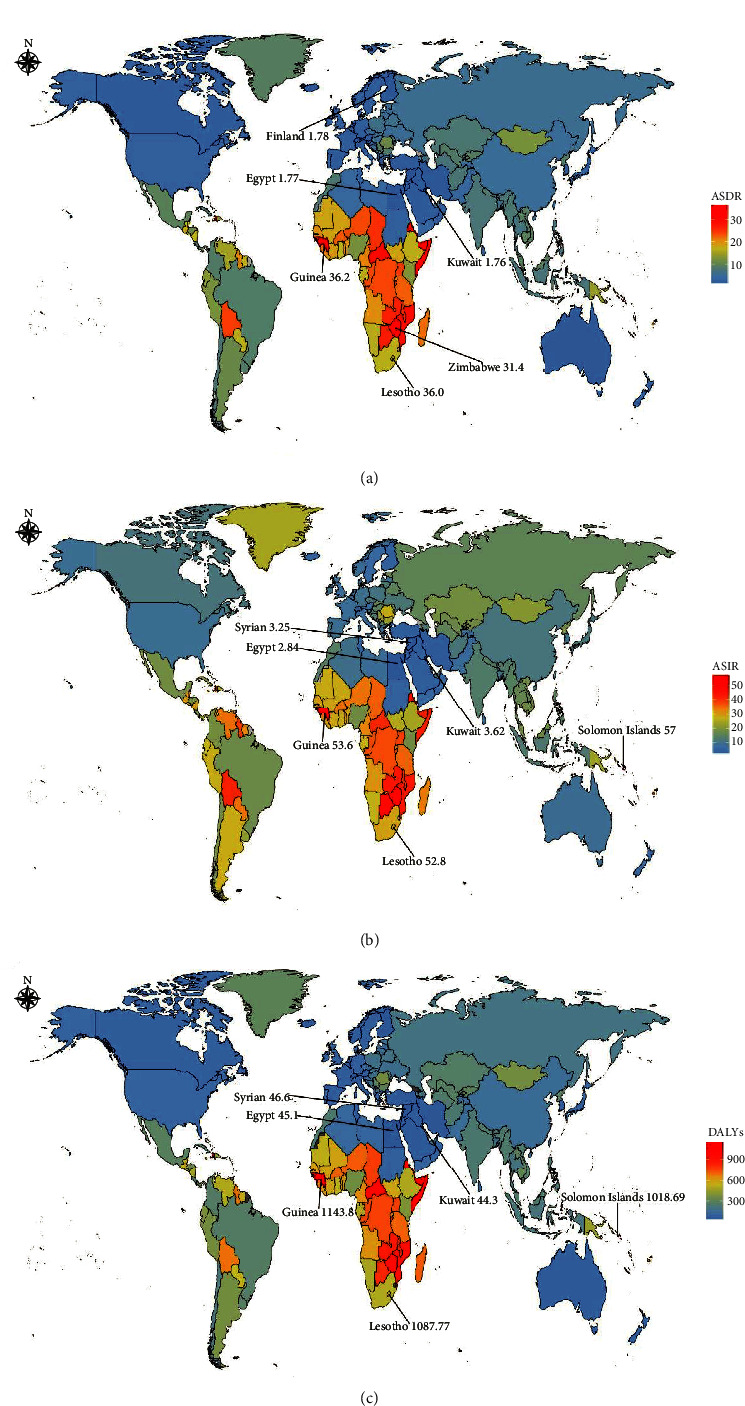
Global disease burden of cervical cancer in 192 countries. (a) The ASDR of cervical cancer in 2019. (b) The ASIR of cervical cancer in 2019. (c) The age-standardized DALY rate of cervical cancer in 2019. ASDR, age-standardized death rate; ASIR, age-standardized incidence rate.

From the perspective of countries in the GBD region, more than 90% of countries showed an upward trend in the age-standardized DALY rate. Among them, Guinea, Lesotho, Solomon Islands, Kuwait, Egypt, and the Syrian Arab Republic ranked first and last in the age-standardized DALY rate. At the regional level, the three regions with the highest and lowest age-standardized DALY rates were sub-Saharan Africa (central, eastern and southern), Australia, Western Europe, and the high-income Asia Pacific region. Details were depicted in Figure 6 and Additional file 1: Tables S1 and S2.

### 3.7. Risk Factors Attributable to the Cervical Cancer Burden

To explore potential hazard factors, we analyzed all the attributable risk factors included in the GBD database. In general, unsafe sex was the most important risk factor in all GBD regions, especially in low-SDI regions. Meanwhile, it is worth noting that smoking played an important role in DALY and deaths of cervical cancer. It is worth noting that from 1990 to 2019, the three regions with the largest number of DALY cases caused by unsafe sex were all concentrated in sub-Saharan Africa (central, eastern, and southern). As for smoking, DALY and deaths in Tropical Latin America, South Latin America, and Oceania were most affected. However, the rate of DALY has been on a downward trajectory since 1990 and fell significantly from 1990 to 2010, especially in low-SDI regions with distinct decrease trends (Figure 4, Additional file 1: Table S9).

## 4. Discussion

As the fastest and most convenient way to obtain the statistical data on the epidemiological burden of the vast majority of human diseases in the world, there is no doubt that obtaining the global epidemiological baseline data of cervical cancer from GBD big data has a very strong timeliness and reliability [17–19]. In this study, relying on the GBD database, our innovation mainly has two aspects. First, previous epidemiological studies of cervical cancer were focused on individual countries or regions [20–22], and this study shows the global epidemic trend and development mode of cervical cancer in the past 30 years. Secondly, our research shows that the distribution differences of cervical cancer in different regions of the world (incidence rate, mortality, and DALY)are directly conducive to the formulation of health policies and the better allocation of medical and health resources.

Cervical cancer is highly preventable and easy to treat if detected early. However, in low-income countries lacking in organized screening and prevention programs, the incidence rate and mortality burden of cervical cancer remain high [23]. Recent global data estimate that 527624 new cases and 265672 deaths of cervical cancer can be found every year [24]. Of note, our analysis revealed that the incidence of cervical cancer was higher in Africa and Asia and lower in North America and Europe. In aggregate, the incidence rate of cervical cancer in Western developed countries has been decreasing. It is not surprising that over the past three decades, especially for less-developed regions, the existing health infrastructure has organized the provision of prevention and screening services, access to screening facilities, follow-up management, and the full link between confirmed diagnosis and follow-up treatment. For example, in the United States, due to the extensive implementation of cytology screening, women benefit from this, so the incidence rate and mortality of cervical cancer have been declining [1, 25]. However, according to the international cancer research agency (IARC, 2012) report, Southern Africa reports the highest ASR of cervical cancer worldwide (43.1 per 100,000) [26], and the incidence rate of cervical cancer in Nepal is 19.0/100000, and the age-standardized mortality rate is 12.0/100000 [26–30]. Synchronously, the mortality of cervical cancer also showed a consistent geographical distribution, in line with the result of the Global Cancer Observatory (GCO) (http://gco.iarc.fr). It is worth noting that cervical cancer is the main cause of cancer-related death, especially in Africa, followed by China (with 106,000 cases and 48,000 deaths) and India(with 97,000 cases and 60,000 deaths) in 2018 [6]. Taken together, the gap between countries and regions in HPV vaccination and Medicaid expansion fully illustrates the geographical differences. In addition, for many underdeveloped countries, the lack of resources and infrastructure makes such prevention and treatment plans limited, leading to the early warning of cervical cancer is still difficult.

In this study, the incidence was equipped with a bimodal distribution, especially in people aged 25-44 and 50 years or older, consistent with previous studies [31, 32]. In addition, the incidence in several countries represented the bimodal distribution of age, such as Fiji, Djibouti, Haiti, and Lebanon. On the contrary, some countries like Australia, Greece, Finland, and the United States of America showed a unimodal distribution over the past three decades. In addition, we also observed a phenomenon that the peak of incidence was higher in young women in low-SDI countries and for women with age ≥50 years in high-SDI countries. Previous studies have confirmed that developed countries face serious problems, such as aging and declining birth rates [33, 34]. Given this situation, we can speculate that even in different SDI regions in the same continent, the incidence rate of cervical cancer may be disaccord. Consistent with our assumptions, the incident and death of cervical cancer patients in Australasia, Central Europe, High-income North America, and East Asia tended to be aging; however, in Oceania and sub-Saharan Africa(eastern, western, and central), the overall mortality rate exceeded 10% (aged 20-39). In addition, in recent years, the rapid development of new treatment technologies has led to a significant improvement in the prognosis of cervical cancer, which further promotes this aging trend, while areas with relatively backward economies lag significantly [35, 36]. Taken together, this phenomenon indicates that the global aging and regional imbalance of cervical cancer are deteriorating, which highlights the importance of increasing investment in cervical cancer prevention and cure in different regions.

In this study, we also observed a significant correlation (negative correlation) between the changes of ASDR in the past 30 years and the baseline ASDR in 1990. Among them, compared with low SDI, ASDR with high SDI decreased more significantly, consistent with our previous conclusions. For example, from 1990 to 2019, the downward trend of ASDR is the most obvious. It is famous in Central and Latin America, which shows that countries with high ASDR should pay more attention to the early warning of cervical cancer as a health event, and vice versa. At the same time, in our study, we found that the variation of ASDR is very obvious in different SDI regions, and the age-standardized DALY also shows the opposite correlation with the trend of SDI. These data suggested that the medical resources in developed countries and regions are more abundant, so the benefits of female population are also fully guaranteed. In general, the inconsistent distribution of medical resources between high SDI and low SDI is another reason for the wide disparity of cervical cancer incidence rate in various countries and regions. Although the health care reform has been put into practice, some progress has been made in the past, but little effect has been achieved.

Cervical cancer is developed through persistent infection with high-risk human papillomavirus (hrHPV), which is the main cause of death of women all over the world [37].

Previous studies have shown that the increased rate of early sexual behavior and human papillomavirus (HPV) infection has increased the incidence of cervical cancer in young women [38]. Meanwhile, smoking will further increase the risk of high-level cervical lesions in women with persistent high-risk HPV infection [39]. Consistent with the results reported in previous studies, unsafe sex was the greatest distributor of DALY in all GBD regions, especially in low-SDI regions. It is not surprising that cervical screening and HPV vaccination have been popularized in high-income countries. However, the coverage of less-developed countries is still unsatisfactory [32]. For example, in sub-Saharan Africa, the incidence rate of young cervical cancer patients remains high, mainly due to the high prevalence of HPV and human immunodeficiency virus (HIV) infection [40, 41]. Indeed, multiple sexual partners, precocious behavior, and a high HIV incidence rate contribute to a higher chance of cervical cancer among young people (15 to 24 years old) [42]. Collectively, knowledge and awareness of HPV and HIV are far from satisfactory across developing countries, which provides a challenge to prevent the younger trend of cervical cancer.

Despite several strengths, there are some noteworthy limitations to this study. First, although our study provides some insights for a comprehensive assessment of the distribution and development trend of cervical cancer, this study has not yet explored the uncertainty between the years of healthy life loss of patients in various regions, which may lead to the underestimation of the uncertainty of DALY. Second, this study did not report the disease burden associated with each pathological type (such as squamous cell carcinoma, adenocarcinoma, and adenosquamous cell carcinoma), which may be difficult to provide targeted prevention suggestions worldwide. Third, we have not stratified the epidemic trend of cervical cancer between urban and rural areas, which leaves a regret for policy-making in economically underdeveloped areas. Therefore, future research should focus on disease subtypes, economic development, and geographical zoning reports.

## 5. Conclusions

Globally, in the past 30 years, the incidence rate and mortality of cervical cancer have gradually increased. It is worth paying attention to that the incidence rate in some economically backward areas is rising rapidly, such as middle-income countries and low - and middle-income countries. The primary cause of death and DALY in patients with cervical cancer is unsafe sexual behavior, followed by smoking. Although there are still difficulties in establishing AIDS/STD prevention services and collecting public health resource allocation, further efforts are still needed to reduce the growing burden of cervical cancer.

## Figures and Tables

**Table 1:** Incidence cases, age-standardized incidence, and temporal trends of cervical cancer from 1990 to 2019.

	1990		2019		1990-2019
	Incident cases no. ^*∗*^102 (95% UI)	ASIR per 100,000 No. (95% UI)	Incident cases no. ^*∗*^102 (95% UI)	ASIR per 100,000 No. (95% UI)	EAPC No. (95% CI)
Overall	3356.42 [3003.54-3938.93]	14.91 [13.37-17.55]	5655.41 [4815.24-6364.35]	13.35 [11.37-15.03]	−0.38 [−0.41 to −0.34]

Socio-demographic index					
High SDI	596.9 [542.98-616.54]	11.83 [10.67-12.22]	638.64 [557.1-714.55]	8.91 [7.74-9.99]	−0.95 [−1.06 to −0.85]
High-middle SDI	758.05 [715.34-888.76]	12.77 [12.05-15]	1131.23 [897.8-1291.53]	11.59 [9.18-13.24]	−0.27 [−0.31 to −0.22]
Middle SDI	921.78 [814.53-1164]	14.87 [13.17-18.85]	1833.37 [1444.92-2088.59]	13.44 [10.61-15.28]	−0.29 [−0.34 to −0.24]
Low-middle SDI	662.16 [540.58-817.63]	18.04 [14.86-22.49]	1259.63 [1078.83-1501.05]	15.78 [13.57-18.87]	−0.56 [−0.65 to −0.46]
Low SDI	414.99 [317.75-508.02]	27.74 [21.56-34.25]	788.21 [616.13-979.25]	23.21 [18.31-28.76]	−0.69 [−0.73 to −0.65]

Region					
Andean Latin America	41 [34.53-48.59]	33.39 [28.2-39.63]	91 [69.29-116.15]	29.74 [22.67-37.83]	−0.53 [−0.66 to −0.4]
Australasia	13.73 [11.47-14.67]	11.83 [9.76-12.65]	16.48 [12.7-21.14]	8.22 [6.32-10.59]	−0.98 [−1.37 to −0.59]
Caribbean	41.2 [33.3-47.16]	28 [22.69-31.86]	68.62 [53.57-85]	26.23 [20.41-32.58]	−0.24 [−0.3 to −0.19]
Central Asia	52.74 [48.97-56.29]	18.58 [17.37-19.85]	76.66 [66.47-88.3]	16 [13.94-18.4]	−0.34 [−0.46 to −0.23]
Central Europe	153.92 [143.9-162.15]	20.67 [19.23-21.74]	136.77 [112.58-158.96]	15.8 [12.97-18.48]	−1.08 [−1.22 to −0.95]
Central Latin America	170.8 [158.04-178.45]	32.3 [29.43-33.73]	284.79 [231.09-350.27]	21.45 [17.44-26.37]	−1.77 [−1.93 to −1.61]
Central sub-Saharan Africa	58.43 [39.54-78.26]	37.38 [25.91-49.42]	122.97 [82.33-168.78]	32.32 [21.74-44.74]	−0.51 [−0.61 to −0.41]
East Asia	452.57 [353.84-793.61]	9 [7.08-15.63]	1153.77 [643.46-1471.15]	11.17 [6.25-14.26]	1.33 [1.11 to 1.56]
Eastern Europe	228.21 [196.72-246.5]	14.53 [12.66-15.79]	229.97 [189.12-280.32]	14.76 [11.91-18.14]	0.03 [−0.13 to 0.19]
Eastern sub-Saharan Africa	190.81 [144.07-238.1]	38.27 [28.81-47.55]	363.35 [257.56-484.49]	31.79 [22.9-41.68]	−0.8 [−0.87 to −0.72]
High-income Asia Pacific	124.66 [116.36-143.57]	11.65 [10.81-13.42]	150.61 [119.08-179.61]	10.33 [7.99-12.4]	−0.17 [−0.28 to −0.05]
High-income North America	175.3 [151.07-182.59]	10.39 [8.89-10.83]	218.52 [174.25-266.17]	8.93 [7.09-10.93]	−0.58 [−0.72 to −0.43]
North Africa and Middle East	70.32 [50.28-80.27]	6.9 [4.92-7.88]	146.26 [111.39-176.32]	5.78 [4.43-6.89]	−0.63 [−0.69 to −0.57]
Oceania	5.7 [4.04-7.57]	29.58 [21.39-39.83]	13.29 [8.6-18.2]	28.22 [19-38.09]	−0.05 [−0.12 to 0.03]
South Asia	563.56 [442.09-685.94]	16.04 [12.64–19.66]	1000.2 [801.06-1247.7]	12.37 [9.94-15.46]	−1.09 [−1.23 to −0.96]
Southeast Asia	311.27 [235.2-386.84]	18.75 [14.3-23.63]	520.62 [419.29-686.68]	14.48 [11.73-19]	−1.06 [−1.17 to −0.96]
Southern Latin America	64.79 [60.51-68.66]	26.3 [24.53-27.91]	98.44 [72.73-128.55]	24.85 [18.23-32.74]	−0.38 [−0.52 to −0.24]
Southern sub-Saharan Africa	61.71 [46.79-75.3]	33.33 [25.19-40.62]	120.21 [97.4-144.45]	32.9 [26.88-39.48]	0.28 [0.05 to 0.52]
Tropical Latin America	141.19 [133.57-163.6]	24.52 [23.12-28.28]	237.4 [221.28-271.79]	17.91 [16.69-20.43]	−1.29 [−1.4 to −1.19]
Western Europe	286.02 [259.1-296.83]	11.19 [9.88-11.62]	271.74 [226.94-317.02]	8.26 [6.85-9.68]	−0.97 [−1.07 to −0.87]
Western sub-Saharan Africa	148.49 [116.61-186.36]	28.64 [22.5-35.8]	333.74 [26137-425.35]	25.47 [20.17-31.94]	−0.35 [−0.4 to −0.31]

**Table 2:** Death cases, age-standardized death rate, and temporal trends of cervical cancer from 1990 to 2019.

	1990		2019		1990-2019
	Death cases No. ^*∗*^102 (95% UI)	ASDR per 100,000 No. (95% UI)	Death cases No. ^*∗*^102 (95% UI)	ASDR per 100,000 No. (95% UI)	EAPC No. (95% CI)
Overall	1845.27 [1648.36-2189.42]	8.48 [7.59-10.07]	2804.79 [2388.64-3139.3]	6.51 [5.55-7.29]	−0.93 [−0.98 to −.88]

Sociodemographic index					
High SDI	252.22 [232.75-261.93]	4.56 [4.22-4.71]	261.73 [228.23-281.49]	2.9 [2.6-3.1]	−1.57 [−1.68 to −1.46]
High-middle SDI	413.53 [386.94-484.05]	6.95 [6.5-8.13]	517.71 [416.64-578.74]	4.89 [3.92-5.47]	−1.25 [−1.31 to −1.19]
Middle SDI	525.26 [466.34-651.21]	9.32 [8.31-11.54]	901 [713.33-1032]	6.78 [5.4-7.76]	−1.03 [−1.09 to −0.97]
Low-middle SDI	392.09 [324.61-500.54]	11.71 [9.73-15.05]	666.78 [572.7-812.45]	8.85 [7.62-10.83]	−1.04 [−1.12 to −0.96]
Low SDI	260.8 [202.34-321.11]	19.18 [15-23.66]	455.4 [357.97-562.58]	15.05 [11.92-18.46]	−0.9 [−0.94 to −0.86]

Region					
Andean Latin America	23.31 [19.62-27.59]	20.39 [17.22-24.05]	42.78 [33.17-53.82]	14.37 [11.18-18.04]	−1.33 [−1.45 to −1.21]
Australasia	4.55 [3.91–4.77]	3.73 [3.16-3.92]	5.25 [4.48-5.83]	2.17 [1.88-2.4]	−1.57 [−1.97 to −1.17]
Caribbean	22.27 [17.46-25.49]	15.83 [12.55-18.04]	34.7 [27.24-42.61]	12.95 [10.11-15.96]	−0.65 [−0.72 to −0.58]
Central Asia	27.2 [24.8-28.87]	9.81 [8.95-10.43]	34.23 [30-39.27]	7.58 [6.68-8.7]	−0.75 [−0.88 to −0.61]
Central Europe	79.98 [76.03-85.32]	10.14 [9.63-10.79]	68.83 [58.24-79.88]	6.65 [5.59-7.75]	−1.57 [−1.69 to −1.46]
Central Latin America	95.87 [86.96-100.11]	20.35 [18.19-21.28]	138.31 [115.34-168.04]	10.65 [8.91-12.92]	−2.61 [−2.76 to −2.45]
Central sub-Saharan Africa	37.21 [26.09-48.39]	26.27 [18.78-34.25]	72.96 [49.08-100.58]	21.67 [14.49-30.24]	−0.67 [−0.79 to −0.54]
East Asia	284.04 [223.19-461.41]	6.05 [4.77-9.76]	559.6 [331.87-713.62]	5.18 [3.09-6.59]	−0.05 [−0.29 to 0.19]
Eastern Europe	129.39 [109.52-138.59]	7.62 [6.5-8.19]	100.37 [84.72-119.1]	5.54 [4.62-6.61]	−1.38 [−1.55 to −1.21]
Eastern sub-Saharan Africa	119.37 [90.76-149.22]	26.51 [20.07-33.45]	211.12 [154.77-278.56]	21.13 [15.15-27.62]	−0.9 [−0.96 to −0.84]
High-income Asia Pacific	46.42 [43.47-53.22]	4.2 [3.93-4.81]	56.04 [45.77-62.17]	2.7 [2.22-2.96]	−1.52 [−1.59 to −1.44]
High-income North America	67.41 [59.7-70.44]	3.71 [3.25-3.86]	87.99 [74.75-93.4]	2.99 [2.55-3.15]	−0.69 [−0.83 to −0.55]
North Africa and Middle East	39.71 [28.19-45.3]	4.37 [3.1-4.99]	70.05 [54.43-83.11]	3.15 [2.47-3.69]	−1.11 [−1.18 to −1.04]
Oceania	3.05 [2.19-4.21]	18.16 [13.38-25.22]	6.69 [4.48-9.13]	16.41 [11.5-22.19]	−0.18 [−0.27 to −0.08]
South Asia	333.37 [262.23-399.53]	10.49 [8.29-12.62]	533.03 [428.71-699.46]	7.01 [5.66-9.21]	−1.6 [−1.72 to −1.48]
Southeast Asia	167.14 [125.65-214.67]	11.05 [8.36-14.54]	251.29 [205.25-349.8]	7.36 [6.03-10.33]	−1.52 [−1.61 to −1.43]
Southern Latin America	30.67 [28.87-33.08]	12.34 [11.62-13.3]	41.76 [35.52-45.98]	9.64 [8.17-10.56]	−1.01 [−1.13 to −0.89]
Southern sub-Saharan Africa	32.39 [24.83-40.68]	19.17 [14.63-24.27]	65.61 [53.9-77.52]	19.34 [15.82-22.77]	0.46 [0.18 to 0.73]
Tropical Latin America	76.85 [72.12-88.85]	14.72 [13.7-17.12]	115.8 [107.15-136.57]	8.69 [8.04-10.23]	−2.01 [−2.11 to −1.92]
Western Europe	131.3 [120.35-136.07]	4.36 [3.99-4.51]	117.52 [102.71-126.89]	2.65 [2.38-2.85]	−1.65 [−1.76 to −1.54]
Western sub-Saharan Africa	93.78 [75.57-123.09]	19.74 [15.94-25.76]	190.88 [150.41-240.11]	16.83 [13.38-21]	−0.48 [−0.53 to −0.43]

**Table 3:** Disability-adjusted life-years, age-standardized disability-adjusted life-years, and temporal trends of cervical cancer from 1990 to 2019.

	1990		2019		1990-2019
	Age-standardized		Age-standardized		EAPC No. (95% UI)
	DALY No. ^*∗*^103 (95% UI)	DALY rate per 100,000 No. (95% UI)	DALY No. ^*∗*^103 (95% UI)	DALY rate per 100,000 No. (95% CI)	
Southeast Asia	592.31 [440.72-733.9]	354.96 [266.86-447.24]	808.25 [653.21-1088.29]	223.36 [181.44-302.65]	−1.73 [−1.83 to −1.63]
Southern Latin America	101.76 [96.27-107.78]	413.34 [390.66-437.19]	127.49 [105.4-140.05]	317.23 [260.3-348.08]	−1.07 [−1.18 to −0.95]
Southern sub-Saharan Africa	116.35 [89.85-142.79]	633.63 [488.91-782.66]	213.94 [173.97-254.95]	586.79 [476.2-698.37]	0.23 [−0.05 to 0.52]
Tropical Latin America	262.8 [247.91-303.91]	455.15 [428.99-524.5]	365.28 [340.28-419.75]	274.27 [255.5-314.34]	−1.95 [−2.06 to −1.84]
Western Europe	351.5 [321.56-363.85]	134.35 [119.97-138.99]	277.36 [248.48-299.74]	79.19 [71.88-85.34]	−1.77 [−1.9 to −1.63]
Western sub-Saharan Africa	327.53 [261.59-424.73]	626.47 [502.32-815.1]	672.6 [524.74-854.86]	507.97 [398.99-640.76]	−0.69 [−0.74 to −0.64]

## Data Availability

In this study, the data of cervical cancer disease burden were obtained from the online Global Health Data Exchange (GHDx) query tool (https://ghdx.healthdata.org/gbd-results-tool).

## Abbreviations

GBD: The global burden of disease
GCO: Global cancer observatory
GHDx: Global Health Data Exchange
SDG: Sustainable development goal
EAPC: Estimated annual percentage changes
SDI: Social-demographic index
WHO: World Health Organization
ASIR: Age-standardized incidence rate
ASDR: Age-standardized death rate
DALY: Disability-adjusted life-years.

## Supplementary Materials

Supplementary Materials Supplement Table S1. Three countries with the largest and lowest number of incidence, death, or DALY. Supplement Table S2. Three regions with the largest and lowest number of incidence, death, or DALY. Supplement Table S3. The incident cases and age-standardized incidence rate of cervical cancer in 1990 and 2019, and its temporal trends from 1990 to 2019. Supplement Table S4. The death cases and age-standardized death rate of cervical cancer in 1990 and 2019, and its temporal trends from 1990 to 2019. Supplement Table S5. The DALY and age-standardized DALY rate of cervical cancer in 1990 and 2019, and its temporal trends from 1990 to 2019. Supplement Table S6. Age distribution of incidence (per 100,000) for cervical cancer in different countries in 2019. Supplement Table S7. Age distribution of death rate (per 100,000) for cervical cancer in different countries in 2019. Supplement Table S8. Age distribution of DALY rate (per 100,000) for cervical cancer in different countries in 2019. Supplement Table S9. The cervical cancer death rate and DALY attributable to risk factors among different SDI quantiles from 1990 to 2019. Supplementary Figure 1. The incident cases (A), age-standardized incidence (B), death (C), and DALY (D) rates of cervical cancer from 1990 to 2019. Supplementary Figure 2. The EAPC of cervical cancer ASR from 1990 to 2019, by SDI and region. (A) The EAPC of ASIR, (B) the EAPC of ASDR, (C) the EAPC of age-standardized DALY rate, and (D) the ratio of incidence among different age groups in 2019. Supplementary Figure 3. The age-standardized incidence (A), death (B), and DALY (C) rates of cervical cancer per 100,000 population among regions based on SDI in 2019. Supplementary Figure 4. The proportion of different ages in cervical cancer death (A) and incidence (B) and by years. Supplementary Figure 5. Distribution of different ages in cervical incidence (A), death(B), and DALY(B) among different SDI quantiles from 1990 to 2019. Supplementary Figure 6. The global EAPC of cervical cancer in 192 countries. (A). The EAPC of ASDR. (B). The EAPC of ASIR. (C) The EAPC of age-standardized DALY rate. ASDR, age-standardized death rate; ASIR, age-standardized incidence rate; EAPC, estimated annual percentage changes.
