# Supplementary material for: Epidemiological Trends and Attributable Risk Burden of Cervical Cancer: An Observational Study from 1990 to 2019
Source: Int J Clin Pract. 2022 Sep 30;2022:3356431. doi: 10.1155/2022/3356431 (PMC9546700; doi:10.1155/2022/3356431)
Supplement: Supplementary Materials — Supplement Table S1. Three countries with the largest and lowest number of incidence, death, or DALY. Supplement Table S2. Three regions with the largest and lowest number of incidence, death, or DALY. Supplement Table S3. The incident cases and age-standardized incidence rate of cervical cancer in 1990 and 2019, and its temporal trends from 1990 to 2019. Supplement Table S4. The death cases and age-standardized death rate of cervical cancer in 1990 and 2019, and its temporal trends from 1990 to 2019. Supplement Table S5. The DALY and age-standardized DALY rate of cervical cancer in 1990 and 2019, and its temporal trends from 1990 to 2019. Supplement Table S6. Age distribution of incidence (per 100,000) for cervical cancer in different countries in 2019. Supplement Table S7. Age distribution of death rate (per 100,000) for cervical cancer in different countries in 2019. Supplement Table S8. Age distribution of DALY rate (per 100,000) for cervical cancer in different countries in 2019. Supplement Table S9. The cervical cancer death rate and DALY attributable to risk factors among different SDI quantiles from 1990 to 2019. Supplementary Figure 1. The incident cases (A), age-standardized incidence (B), death (C), and DALY (D) rates of cervical cancer from 1990 to 2019. Supplementary Figure 2. The EAPC of cervical cancer ASR from 1990 to 2019, by SDI and region. (A) The EAPC of ASIR, (B) the EAPC of ASDR, (C) the EAPC of age-standardized DALY rate, and (D) the ratio of incidence among different age groups in 2019. Supplementary Figure 3. The age-standardized incidence (A), death (B), and DALY (C) rates of cervical cancer per 100,000 population among regions based on SDI in 2019. Supplementary Figure 4. The proportion of different ages in cervical cancer death (A) and incidence (B) and by years. Supplementary Figure 5. Distribution of different ages in cervical incidence (A), death(B), and DALY(B) among different SDI quantiles from 1990 to 2019. Supplementary Figure 6. T [file 3356431.f1.pdf]

Supplementary Tables

Supplement Table S1. Three countries with the largest and lowest number of incidence, death, or DALY.

Supplement Table S2. Three regions with the largest and lowest number of incidence, death, or DALY.

Supplement Table S3. The incident cases and age-standardized incidence rate of cervical cancer in 1990 and 2019, and its temporal trends from 1990 to 2019.

Supplement Table S4. The death cases and age-standardized death rate of cervical cancer in 1990 and 2019, and its temporal trends from 1990 to 2019.

Supplement Table S5. The DALY and age-standardized DALY rate of cervical cancer in 1990 and 2019, and its temporal trends from 1990 to 2019.

Supplement Table S6. Age distribution of incidence (per 100,000) for cervical cancer in different countries in 2019.

Supplement Table S7. Age distribution of death rate (per 100,000) for cervical cancer in different countries in 2019.

Supplement Table S8. Age distribution of DALYs rate (per 100,000) for cervical cancer in different countries in 2019.

Supplement Table S9. The cervical cancer death rate and DALYs attributable to risk factors among different SDI quantiles from 1990 to 2019.

Supplementary Figures

Supplementary Figure1. The incident cases (A), age standardized incidence (B), death (C) and DALY (D) rates of cervical cancer from 1990 to 2019.

Supplementary Figure2. The EAPC of cervical cancer ASR from 1990 to 2019, by SDI and region. (A) The EAPC of ASIR; (B) The EAPC of ASDR; (C) The EAPC of age-standardized DALY rate;(D) The ratio of incidence among different age groups in 2019.

Supplementary Figure3. The age standardized incidence (A), death (B) and DALY (C) rates of cervical cancer per 100,000 population among regions based on SDI in 2019.

Supplementary Figure4. The proportion of different ages in cervical cancer death (A) and incidence (B) and by years.

Supplementary Figure5. Distribution of different ages in cervical incidence(A), death(B), and DALYs(B) among different SDI quantiles from 1990 to 2019.

Supplementary Figure6. The global EAPC of cervical cancer in 192 countries. (A). The

EAPC of ASDR. (B). The EAPC of ASIR.(C) The EAPC of age-standardized DALY rate. ASDR, age-standardized death rate; ASIR, age-standardized incidence rate; EAPC, estimated annual percentage changes.

Supplement Table S1 Three countries with the largest and lowest number of incidence, death, or DALY.

Measure	Top three countries			Bottom three countries		
2019ASR (per 100,000 people)						
ASIR	Solomon Islands 57	Guinea 53.61	Lesotho 52.77	Egypt 2.84	Syrian Arab Republic 3.25	Kuwait 3.62
ASDR	Guinea 36.16	Lesotho 35.96	Zimbabwe 31.39	Kuwait 1.76	Egypt 1.77	Finland 1.78
Age Standardized DALY Rate	Guinea 1143.8	Lesotho 1087.77	Solomon Islands 1018.69	Kuwait 44.34	Egypt 45.13	Syrian Arab Republic 46.56
1990-2019 increase times						
Incidence(cases)	United Arab Emirates 501.17	Saudi Arabia 453.61	Qatar 431.94	Denmark -46.3	Latvia -45.66	Ukraine -43.72
Death(cases)	United Arab Emirates 349.62	Guatemala 276.37	Qatar 269.66	Ukraine -50.7	Denmark -48.58	Latvia -46.4
DALY(Year)	United Arab Emirates 409.37	Qatar 264.9	Guatemala 239.23	Denmark -54.73	Latvia -53.52	Ukraine -50.22
EAPC						
Incidence	Lesotho 3.43	Italy 2.02	China 1.61	Maldives -3.68	Singapore -3.4	Austria -2.98
Death	Lesotho 3.25	Zimbabwe 1.46	Bulgaria 1.2	Maldives -4.54	Singapore -4.04	Bermuda -3.56
DALY	Lesotho 3.44	Zimbabwe 1.67	Bulgaria 1.23	Maldives -5.06	Singapore -4.31	Republic of Korea -3.91

Supplement Table S2 Three regions with the largest and lowest number of incidence, death, or DALY.

Measure	Top three regions			Bottom three regions		
2019ASR (per 100,000 people)						
ASIR	Southern Sub-Saharan Africa 32.9	Central Sub-Saharan Africa 32.32	Eastern Sub-Saharan Africa 31.79	North Africa and Middle East 5.78	Australasia 8.22	Western Europe 8.26
ASDR	Central Sub-Saharan Africa 21.67	Eastern Sub-Saharan Africa 21.13	Southern Sub-Saharan Africa 19.34	Australasia 2.17	Western Europe 2.65	High-income Asia Pacific 2.7
Age Standardized DALY Rate	Central Sub-Saharan Africa 678.72	Eastern Sub-Saharan Africa 660.28	Southern Sub-Saharan Africa 586.79	Australasia 65.47	Western Europe 79.19	High-income Asia Pacific 85.67
1990-2019 increase in the number of cases/years (-fold)						
Incidence(cases)	East Asia 1.55	Oceania 1.33	Western Sub-Saharan Africa 1.25	Central Europe -0.11	Western Europe -0.05	Eastern Europe 0.01
Death(cases)	Oceania 1.19	Western Sub-Saharan Africa 1.04	Southern Sub-Saharan Africa 1.03	Eastern Europe -0.22	Central Europe -0.14	Western Europe -0.1
DALY(Year)	Oceania 1.2	Western Sub-Saharan Africa 1.05	Central Sub-Saharan Africa 0.93	Central Europe -0.24	Western Europe -0.21	Eastern Europe -0.16
EAPC						
Incidence	East Asia 1.33	Southern Sub-Saharan Africa 0.28	Eastern Europe 0.03	Central Latin America -1.77	Tropical Latin America -1.29	South Asia -1.09
Death	Southern Sub-Saharan Africa 0.46	East Asia -0.05	Oceania -0.18	Central Latin America -2.61	Tropical Latin America -2.01	Western Europe -1.65
DALY	Southern Sub-Saharan Africa 0.23	East Asia 0.02	Oceania -0.17	Central Latin America -2.48	Tropical Latin America -1.95	Australasia -1.83

Supplementary Table S3. The incident cases and age-standardized incidence rate of Cervical cancer in 1990 and 2019, and its temporal trends from 1990 to 2019.

Nation	Incident cases No.(95% UI)		change absolute number(%)	ASIR per 100,000 No.(95% UI)		1990-2019 EAPC No. (95% CI)
	1990	2019		1990	2019	
Afghanistan	493.44 [149.24-767.51]	1069.57 [379.83-1698.83]	116.76	12.79 [4.02-19.73]	11.39 [4.58-17.47]	-0.52 [-0.68 to -0.36]
Albania	103.23 [90.1-139.42]	140.41 [97.17-194.35]	36.02	8.06 [7.07-11]	7.98 [5.5-11.18]	0.23 [0.05 to 0.42]
Algeria	1028.01 [683.4-1351.63]	1917.5 [1303-2692.61]	86.53	13.71 [9.32-17.87]	9.48 [6.64-12.96]	-1.28 [-1.43 to -1.14]
American Samoa	2.38 [1.87-3.28]	4.85 [3.64-6.33]	103.78	16.83 [13.45-22.98]	18.65 [14-24.23]	0.44 [0.27 to 0.61]
Andorra	3.92 [2.76-5.45]	7.52 [5.26-10.28]	91.84	13.41 [9.44-18.58]	12.49 [8.68-16.96]	-0.33 [-0.43 to -0.24]
Angola	1002.32 [644.03-1480.98]	2596.65 [1614.97-3845.55]	159.06	36.67 [23.69-52.94]	30.31 [19.32-44.76]	-0.8 [-0.94 to -0.67]
Antigua and Barbuda	6.61 [5.68-7.67]	11.95 [9.59-14.6]	80.79	23.31 [19.97-27.17]	21.44 [17.36-26.27]	-0.45 [-0.59 to -0.31]
Argentina	3938.63 [3625.32-4307.97]	7281.87 [5168.87-9649.49]	84.88	23.71 [21.77-25.97]	28.02 [19.75-37.24]	0.39 [0.22 to 0.57]
Armenia	315.27 [281.84-350.99]	322.96 [259.87-398.73]	2.44	19.15 [17.25-21.52]	15.61 [12.47-19.13]	-0.63 [-0.89 to -0.37]
Australia	1096.64 [955.39-1185.47]	1446.45 [1070.89-1895]	31.90	11.3 [9.74-12.23]	8.55 [6.36-11.27]	-0.7 [-1.07 to -0.33]
Austria	873.03 [704.88-947.98]	506.17 [399.41-682.35]	−42.02	16.22 [12.91-17.73]	7.28 [5.59-9.66]	-2.98 [-3.22 to -2.74]
Azerbaijan	440 [373.29-530.67]	704.63 [518.27-987.44]	60.14	13.94 [11.94-17.05]	11.88 [8.77-16.66]	-0.67 [-0.78 to -0.56]
Bahamas	29.75 [25.54-33.94]	53.93 [41-69.88]	81.28	27.98 [24.24-31.69]	23.82 [18.16-30.8]	-0.74 [-0.85 to -0.62]
Bahrain	8.49 [6.8-10.86]	29.26 [21.79-39.26]	244.64	7.8 [6.35-10.13]	5.65 [4.29-7.4]	-1.21 [-1.43 to -1]
Bangladesh	5370.69 [2393.12-7044.64]	7407.12 [4071.32-11533.27]	37.92	19.35 [8.73-25.15]	10.07 [5.5-15.46]	-2.09 [-2.36 to -1.81]
Barbados	46.49 [41.15-51.4]	62.06 [49.4-76.85]	33.49	32.55 [28.68-36.2]	27.97 [22.26-34.78]	-0.5 [-0.55 to -0.44]
Belarus	1138.66 [1047.85-1255.72]	1114.8 [819.41-1551.38]	−2.10	16.42 [15.08-18.13]	15.41 [11.18-21.48]	-0.5 [-0.65 to -0.35]
Belgium	702.65 [605.7-764.89]	630.96 [475.79-810.49]	−10.20	10.27 [9.05-11.29]	7.49 [5.58-9.75]	-1.14 [-1.25 to -1.03]
Belize	17.32 [15.09-20.07]	63.1 [51.12-75.5]	264.32	33.57 [29.43-38.55]	35.78 [29.22-42.55]	-0.08 [-0.48 to 0.32]
Benin	407.55 [313.21-540.45]	1024.09 [700.73-1470.94]	151.28	32.25 [25.53-42.38]	30.15 [21.48-42.28]	-0.17 [-0.23 to -0.12]

Nation	Incident cases No.(95% UI)		change absolute number(%)	ASIR per 100,000 No.(95% UI)		1990-2019 EAPC No. (95% CI)
	1990	2019		1990	2019	
Bermuda	5.36 [4.57-6.22]	4.43 [3.48-5.68]	−17.35	15.04 [12.85-17.45]	8.34 [6.44-10.74]	-2.57 [-2.8 to -2.35]
Bhutan	32.2 [14.82-46.12]	41.19 [23.83-66.45]	27.92	20.04 [9.16-28.38]	12.94 [7.61-20.35]	-1.72 [-1.98 to -1.46]
Bolivia (Plurinational State of)	971.65 [616.94-1238.21]	2070.18 [1435.28-2857.83]	113.06	49.06 [31.72-62.24]	41.59 [29.32-57.22]	-0.77 [-0.9 to -0.65]
Bosnia and Herzegovina	298.22 [261.38-357.55]	336.38 [225.87-439.17]	12.80	12.2 [10.74-14.72]	13.47 [8.94-18]	0.41 [0.23 to 0.59]
Botswana	141.6 [89.2-218.61]	476.04 [272.5-747.46]	236.19	37.72 [24.27-57.1]	47.63 [28.09-73.79]	0.77 [0.52 to 1.01]
Brazil	13670.95 [12922.02-15883.92]	22650.54 [21142.19-26301.08]	65.68	24.32 [22.91-28.18]	17.51 [16.34-20.26]	-1.34 [-1.44 to -1.23]
Brunei Darussalam	29.77 [21.07-38.25]	52.85 [40.17-70.49]	77.53	38.78 [28.48-48.5]	25.04 [19.58-32.57]	-1.49 [-1.73 to -1.25]
Bulgaria	1024.18 [906.12-1155.18]	1163.69 [776.85-1525.14]	13.62	18.6 [16.14-20.8]	22.94 [14.44-30.39]	1.56 [1.27 to 1.85]
Burkina Faso	1021.59 [739.79-1369.09]	2275.31 [1620.05-3033.59]	122.72	36.74 [27.05-49.48]	34.46 [25.14-45.25]	-0.25 [-0.41 to -0.1]
Burundi	787.75 [503.46-1094.31]	1171.04 [717.43-1785.72]	48.66	50.06 [32.65-69.32]	38.08 [23.51-56.86]	-1.39 [-1.57 to -1.21]
Cabo Verde	39.05 [32.02-48.04]	58.21 [45.6-86.82]	49.07	30.03 [24.71-38.37]	23.02 [18.09-33.58]	-0.67 [-1.01 to -0.34]
Cambodia	740.37 [411.78-1054.11]	1377.91 [971.66-2072.56]	86.11	22.72 [12.85-32.33]	17.82 [12.77-27.14]	-1.01 [-1.12 to -0.9]
Cameroon	1020.58 [799.76-1373.56]	2733 [1679.23-4175.45]	167.79	35.11 [27.68-46.53]	32.57 [20.76-48.61]	-0.2 [-0.36 to -0.03]
Canada	1865.76 [1697.83-2048.9]	2732.59 [2046.64-3588.21]	46.46	11.38 [10.34-12.53]	11.26 [8.25-14.94]	0.22 [0.06 to 0.39]
Central African Republic	383.71 [245.72-528.4]	650 [375.57-972.71]	69.40	47.14 [30.65-64.29]	40.68 [24.48-61.06]	-0.56 [-0.69 to -0.42]
Chad	567.27 [432.33-779.8]	1250.57 [842.74-1682.15]	120.45	33.47 [25.62-46.44]	35.47 [24.31-47.3]	0.34 [0.24 to 0.45]
Chile	2120.63 [1847.64-2285.49]	2079.78 [1548.8-2740.68]	−1.93	35.29 [30.48-37.92]	18.08 [13.36-24.15]	-2.54 [-2.76 to -2.32]
China	40680.98 [30919.5-73182.36]	109759.9 [58188.68-141538.97]	169.81	8.41 [6.44-15]	11.01 [5.87-14.22]	1.61 [1.35 to 1.88]

Nation	Incident cases No.(95% UI)		change absolute number(%)	ASIR per 100,000 No.(95% UI)		1990-2019 EAPC No. (95% CI)
	1990	2019		1990	2019	
Colombia	2971.35 [2752.53-3311.97]	5154.19 [3852.37-6731.89]	73.46	26.46 [24.61-29.32]	18.73 [13.99-24.45]	-1.63 [-1.84 to -1.41]
Comoros	49.89 [20.29-77.26]	102.5 [63.15-155.64]	105.45	37.65 [16.69-57.37]	34.71 [21.85-51.81]	-0.48 [-0.7 to -0.25]
Congo	339.09 [217.13-461.35]	693.11 [429.97-1036.99]	104.40	47.76 [32.24-64.15]	37.24 [23.63-54.19]	-0.86 [-1.04 to -0.69]
Costa Rica	302.92 [261.93-331.26]	459.73 [342.54-618.4]	51.77	28.16 [24.41-30.66]	16.63 [12.37-22.34]	-2.27 [-2.69 to -1.86]
Cote d'Ivoire	869.66 [644.1-1150.29]	2034.16 [1337.08-2899.89]	133.90	31.4 [23.99-40.64]	28.24 [19.14-39.64]	-0.15 [-0.27 to -0.03]
Croatia	662.11 [546.49-745.55]	425.06 [317.51-557.55]	−35.80	20.12 [16.92-22.69]	12.25 [9-16.24]	-1.6 [-1.96 to -1.24]
Cuba	1259.97 [1104.76-1375.76]	1553.45 [1195.93-1931.44]	23.29	23.6 [20.64-25.75]	19.25 [14.65-24.19]	-0.92 [-1.11 to -0.74]
Cyprus	31.5 [25.19-45.74]	60.79 [42.31-74.45]	92.98	7.61 [6.06-10.98]	6.49 [4.56-8]	-0.39 [-0.57 to -0.22]
Czechia	1246.51 [1155.19-1335.65]	897.89 [717.84-1127.47]	−27.97	18.73 [17.28-20.18]	11.2 [8.86-14.28]	-1.87 [-1.95 to -1.79]
Democratic People's Republic of Korea	2106.34 [1358.84-3588.19]	3145.82 [1991.68-4673.31]	49.35	19.77 [13.05-33.65]	18.46 [11.61-27.49]	-0.08 [-0.18 to 0.03]
Democratic Republic of the Congo	3949.66 [2635.05-5364.95]	8068.21 [5208.5-11294.96]	104.28	36.16 [24.54-48.68]	32.31 [21.05-45.37]	-0.35 [-0.45 to -0.25]
Denmark	679.25 [532.8-730.02]	364.73 [275.18-503.76]	−46.30	19.71 [15.3-21.33]	8.98 [6.63-12.41]	-2.84 [-3.16 to -2.52]
Djibouti	37.97 [22.32-57.33]	139.04 [75.69-249.63]	266.18	36.34 [22.28-52.87]	33.9 [19.41-59.47]	-0.27 [-0.35 to -0.18]
Dominica	13.93 [11.51-16.89]	13.32 [10.1-17.03]	−4.38	41.14 [33.4-49.95]	33.46 [25.16-43.25]	-0.86 [-0.96 to -0.76]
Dominican Republic	468.53 [389.02-611.36]	1311.75 [914.17-1860.59]	179.97	19.78 [16.58-25.62]	25.4 [17.82-36.07]	0.94 [0.77 to 1.1]
Ecuador	909.13 [776.45-1049.59]	2252.66 [1686.51-3045.42]	147.78	28.69 [24.53-33]	27.2 [20.37-36.61]	-0.06 [-0.3 to 0.18]
Egypt	493.15 [418.94-622.91]	969.85 [655.72-1423.06]	96.66	2.86 [2.44-3.69]	2.84 [1.94-4.13]	0.19 [0.09 to 0.28]
El Salvador	491.45 [437.03-633.41]	991.38 [707.89-1362.12]	101.73	27.41 [24.46-35.27]	29.33 [20.92-40.42]	-0.43 [-0.9 to 0.04]

Nation	Incident cases No.(95% UI)		change absolute number(%)	ASIR per 100,000 No.(95% UI)		1990-2019 EAPC No. (95% CI)
	1990	2019		1990	2019	
Equatorial Guinea	50.98 [30.48-74.25]	105.35 [60.92-179.44]	106.65	37.71 [22.52-55.23]	27.74 [16.5-45.72]	-1.08 [-1.23 to -0.94]
Eritrea	344.43 [208.22-530.51]	914.76 [572.15-1358.92]	165.59	42.99 [25.84-66.52]	44.96 [28.1-65.61]	0.24 [0.16 to 0.31]
Estonia	253.13 [220.37-276.48]	168.66 [125.37-219.75]	−33.37	23.53 [20.58-25.9]	16.61 [12.17-21.99]	-1.38 [-1.56 to -1.21]
Eswatini	70.83 [45.93-100.45]	165.62 [83.47-284.03]	133.83	34.31 [22.22-48.24]	41.02 [20.87-70.5]	1.16 [0.54 to 1.79]
Ethiopia	5124.11 [2464.79-8127.28]	6566.86 [4468.65-10642.14]	28.16	38.56 [20.63-60.52]	24.6 [17.1-39.23]	-1.99 [-2.19 to -1.78]
Fiji	117.32 [58.04-156.66]	176.63 [73.47-243.29]	50.55	46.1 [22.97-60.94]	40.44 [16.93-55.32]	-0.08 [-0.4 to 0.24]
Finland	207.1 [187.06-229.67]	240.27 [171.9-314.29]	16.02	5.75 [5.18-6.5]	5.18 [3.8-6.88]	-0.1 [-0.23 to 0.04]
France	4057.92 [3480.87-4362.49]	3888.35 [2939.72-5085.6]	−4.18	10.89 [9.44-11.71]	8.1 [6.02-10.65]	-0.94 [-1.02 to -0.86]
Gabon	116.94 [80.97-157.6]	183.6 [115.77-272.98]	57.00	36.03 [24.93-48.65]	26.93 [17.31-39.47]	-1.12 [-1.37 to -0.87]
Gambia	49.69 [33.08-69.08]	166.11 [111.72-233.01]	234.29	22.04 [14.96-30.35]	26.1 [18.07-36.18]	0.36 [0.12 to 0.59]
Germany	7814.84 [7067.49-8352.93]	6220.26 [4677.35-8459.1]	−20.40	13.8 [11.93-14.87]	9.33 [6.93-12.75]	-1.43 [-1.54 to -1.33]
Ghana	1536.3 [1121.72-2089.75]	3232.86 [2150.48-4530.33]	110.43	35.03 [26.54-47.73]	27.65 [18.63-37.87]	-0.97 [-1.05 to -0.88]
Greece	753.46 [677.43-820.45]	689.08 [527.77-901.91]	−8.54	11.19 [9.88-12.26]	8.28 [6.2-11.03]	-1.09 [-1.29 to -0.88]
Greenland	8.15 [6.42-10.26]	7.64 [5.77-10.07]	−6.26	36.09 [28.79-45.41]	24.62 [18.46-32.45]	-1.75 [-1.93 to -1.56]
Grenada	14.58 [12.6-16.9]	19.4 [15.89-22.9]	33.06	41.28 [35.53-47.95]	34.28 [28.06-40.41]	-0.65 [-0.88 to -0.42]
Guam	7.01 [5.7-8.73]	12.3 [9.27-15.7]	75.46	15.25 [12.6-18.85]	13.65 [10.27-17.49]	-0.75 [-1.07 to -0.43]
Guatemala	517.09 [413.44-904.33]	2214.98 [1609.68-2920.61]	328.35	22.2 [18.01-37.81]	31.18 [22.27-40.9]	1.15 [0.61 to 1.7]
Guinea	1152.33 [880.82-1458.09]	1921.25 [1378.48-2616.18]	66.73	59.45 [45.47-75.24]	53.61 [38.58-72]	-0.23 [-0.28 to -0.18]
Guinea-Bissau	135.65 [88.66-194.04]	249.93 [153.74-357.51]	84.25	47.94 [31.42-67.88]	44.77 [28.38-62.6]	-0.03 [-0.14 to 0.07]
Guyana	115.39 [93.19-140.05]	145 [104.98-193.51]	25.66	45.9 [37.35-55.6]	38.85 [28.34-51.69]	-0.75 [-0.96 to -0.53]
Haiti	1243.17 [489.46-1693.28]	2208.11 [1003.85-3330.53]	77.62	58.11 [24.4-77.15]	44.12 [20.18-65.83]	-0.82 [-0.92 to -0.72]

Nation	Incident cases No.(95% UI)		change absolute number(%)	ASIR per 100,000 No.(95% UI)		1990-2019 EAPC No. (95% CI)
	1990	2019		1990	2019	
Honduras	278.35 [212.09-358.22]	730.78 [435.85-1143.22]	162.54	19.58 [15.07-25.1]	18.99 [11.67-29]	-0.24 [-0.39 to -0.08]
Hungary	1479.69 [1354.75-1611.21]	1066.75 [835.97-1351.99]	−27.91	21.48 [19.47-23.46]	14.27 [10.96-18.31]	-1.77 [-1.94 to -1.6]
Iceland	14.09 [11.94-16.02]	12.68 [10.32-15.34]	−10.01	10.51 [8.87-11.95]	5.77 [4.7-6.96]	-2.37 [-2.55 to -2.19]
India	47408.5 [37133.95-60574.48]	84981.9 [65941.69-110275.84]	79.25	16.65 [13.2-21.45]	13.1 [10.18-17.09]	-1.07 [-1.3 to -0.84]
Indonesia	10404.46 [6474.07-15388.92]	17054.07 [11395.37-26818.41]	63.91	15.86 [10.02-23.65]	12.81 [8.63-20.42]	-0.7 [-0.83 to -0.57]
Iran (Islamic Republic of)	835.21 [580.97-977]	1679.71 [1221.61-1933.2]	101.11	5.36 [3.78-6.38]	3.99 [2.87-4.57]	-1.28 [-1.49 to -1.08]
Iraq	215.6 [154.88-302.96]	689.49 [479.74-968.71]	219.80	4.65 [3.39-6.46]	4.61 [3.25-6.33]	-0.03 [-0.21 to 0.14]
Ireland	209.88 [184.65-233.63]	304.09 [199.42-409.47]	44.89	11.25 [9.72-12.54]	9.64 [6.23-13.04]	-0.11 [-0.31 to 0.09]
Israel	157.94 [140.53-185.7]	345.32 [244.07-454.44]	118.64	6.39 [5.69-7.51]	6.65 [4.63-8.83]	-0.03 [-0.16 to 0.11]
Italy	2027.74 [1903.23-2484.3]	3947.79 [2308.45-5135.96]	94.69	5.06 [4.73-6.21]	7.57 [4.48-9.94]	2.02 [1.69 to 2.35]
Jamaica	288.17 [252.47-314.94]	521.31 [384.25-687.13]	80.90	32.08 [27.97-35.05]	34.14 [25.15-45.04]	0.21 [-0.11 to 0.54]
Japan	9048.09 [8514.78-10079.55]	11105.44 [8100.57-14016.25]	22.74	10.48 [9.86-11.62]	11.17 [7.66-14.29]	0.79 [0.59 to 1]
Jordan	51.81 [38.25-67.19]	160.15 [110.73-215.42]	209.11	6.04 [4.56-7.8]	4.03 [2.88-5.36]	-1.66 [-1.87 to -1.45]
Kazakhstan	1677.83 [1523.41-1868.08]	1939.47 [1608.09-2349.33]	15.59	21.28 [19.4-23.58]	18.48 [15.33-22.34]	0.06 [-0.22 to 0.34]
Kenya	979.05 [643.88-1632.67]	2814.46 [1844.04-4546.74]	187.47	18.54 [12.33-30.34]	18.3 [12.09-29.19]	-0.17 [-0.33 to 0]
Kuwait	24.92 [20.12-29.58]	63.7 [47.2-93.06]	155.62	6.32 [5.08-7.49]	3.62 [2.69-5.1]	-1.59 [-1.86 to -1.33]
Kyrgyzstan	414.86 [354.92-456.91]	549.89 [437.69-660.48]	32.55	23.24 [20.05-25.54]	18.27 [14.68-21.87]	-0.72 [-0.99 to -0.45]
Lao People's Democratic Republic	337.44 [172.11-502.7]	451.07 [274.52-671.1]	33.67	25.47 [13.6-38.45]	15.69 [9.64-23.64]	-1.93 [-2.04 to -1.82]
Latvia	267.7 [232.56-291.62]	145.46 [106.33-200.64]	−45.66	13.89 [12.24-15.18]	8.68 [6.19-12.16]	-1.68 [-1.97 to -1.39]

Nation	Incident cases No.(95% UI)		change absolute number(%)	ASIR per 100,000 No.(95% UI)		1990-2019 EAPC No. (95% CI)
	1990	2019		1990	2019	
Lebanon	94.33 [66.29-121.96]	176.61 [117.63-248.54]	87.23	7.32 [5.24-9.43]	6.08 [4.06-8.52]	-0.73 [-0.85 to -0.61]
Lesotho	166.75 [109.21-246.76]	434.72 [215.09-748.7]	160.70	28.52 [18.78-42.2]	52.77 [26.49-90.4]	3.43 [2.88 to 3.98]
Liberia	204.21 [153.98-274.61]	436.8 [288.59-627.51]	113.90	34.53 [26.42-46.17]	30.99 [20.85-43.31]	-0.39 [-0.51 to -0.27]
Libya	94.37 [63.73-122.99]	276.57 [173.1-378.59]	193.07	9 [6.16-11.68]	8.29 [5.44-11.2]	-0.1 [-0.4 to 0.2]
Lithuania	458.59 [388.92-498.38]	297.39 [231.23-369.26]	−35.15	19.07 [16.44-20.81]	12.43 [9.48-15.81]	-1.36 [-1.6 to -1.12]
Luxembourg	26.3 [23.02-29.44]	22.81 [18.13-29.65]	−13.27	10.26 [8.9-11.56]	5.32 [4.21-6.97]	-2.25 [-2.33 to -2.17]
Madagascar	1362.74 [948.95-1815.89]	2750.3 [1753.11-4053.28]	101.82	40.04 [27.78-52.83]	33.54 [21.59-48.54]	-0.7 [-0.77 to -0.64]
Malawi	1177.69 [847.78-1576.45]	2088.61 [1332.58-3146.28]	77.35	44.52 [32.34-59.2]	39.8 [25.91-57.57]	-0.46 [-0.69 to -0.24]
Malaysia	1239.74 [831.27-1435.34]	2602.39 [1849.22-3446.1]	109.91	21.85 [14.51-25.15]	17.92 [12.6-23.65]	-1.09 [-1.38 to -0.8]
Maldives	11.71 [4.76-16.87]	15.19 [11.69-20.23]	29.72	22.18 [9.36-31.64]	8.78 [6.85-11.47]	-3.68 [-4.02 to -3.34]
Mali	912.22 [689.14-1131.02]	1538.23 [1062.67-2151.44]	68.62	35.28 [26.85-43.72]	28.07 [19.56-38.89]	-1.04 [-1.15 to -0.93]
Malta	16.83 [14.6-19.04]	16.61 [13.28-20.95]	−1.31	7.46 [6.46-8.49]	4.94 [3.92-6.28]	-1 [-1.24 to -0.76]
Marshall Islands	3.95 [2.58-5.87]	8.83 [4.93-14.17]	123.54	36.76 [24.75-56.62]	37.9 [21.94-60.33]	0.03 [-0.11 to 0.16]
Mauritania	239.25 [169.23-315.74]	350.5 [238.59-497.97]	46.50	40.08 [28.38-53.02]	28.01 [19.42-38.91]	-1.04 [-1.15 to -0.94]
Mauritius	80.81 [71.42-90.05]	108.22 [83.76-141.91]	33.92	18.22 [16.17-20.28]	12.09 [9.34-15.7]	-2.07 [-2.3 to -1.84]
Mexico	9583.69 [8177.92-10062.05]	12194.83 [9655.93-16526.58]	27.25	36.09 [29.87-37.98]	18.34 [14.55-24.83]	-2.77 [-2.98 to -2.56]
Micronesia (Federated States of)	11.15 [6.98-17.33]	15.73 [8.76-25.73]	41.08	39.56 [25.6-62.06]	36.21 [20.8-58.83]	-0.29 [-0.42 to -0.16]
Mongolia	181.09 [140.86-241.7]	346.86 [244.29-508.01]	91.54	29.05 [22.7-38.61]	20.97 [15.17-29.49]	-1.55 [-1.74 to -1.36]
Montenegro	45.21 [36.65-58.15]	56.62 [44.95-72.11]	25.24	13.48 [10.9-17.33]	13.58 [10.71-17.24]	-0.02 [-0.38 to 0.34]

Nation	Incident cases No.(95% UI)		change absolute number(%)	ASIR per 100,000 No.(95% UI)		1990-2019 EAPC No. (95% CI)
	1990	2019		1990	2019	
Morocco	1127.71 [753.19-1393.38]	2541.71 [1591.55-3585.04]	125.39	13.89 [9.31-17.07]	14.03 [9.16-19.52]	0.05 [-0.04 to 0.13]
Mozambique	1584.39 [1020.59-2289.73]	3465.69 [2062.66-5214.32]	118.74	39.09 [25.49-55.66]	43.21 [26.52-63.97]	0.56 [0.28 to 0.83]
Myanmar	3597.02 [1947.55-5637.53]	4142.22 [2793.75-6664.13]	15.16	24.27 [13.6-38.86]	14.25 [9.64-23.27]	-2.16 [-2.38 to -1.95]
Namibia	87.92 [57.9-127.06]	249.76 [160.19-375.5]	184.08	20.6 [13.68-29.94]	26.93 [17.67-39.93]	1.17 [1.03 to 1.32]
Nepal	1348.43 [625.67-1849.17]	1890.56 [1162.51-2756.87]	40.20	22.23 [10.42-30.33]	13.91 [8.66-20.11]	-1.69 [-2.14 to -1.24]
Netherlands	784.51 [698.65-852.66]	842.33 [631.41-1077.88]	7.37	8.34 [7.41-9.11]	6.97 [5.11-9.13]	-0.71 [-0.82 to -0.59]
New Zealand	276.18 [183.64-304.16]	201.81 [152.44-264.32]	−26.93	14.52 [9.5-16.06]	6.36 [4.75-8.52]	-2.59 [-3.06 to -2.12]
Nicaragua	388.33 [312.92-445.88]	829.21 [639.56-1119.73]	113.53	36.36 [28.9-41.26]	29.76 [23.27-40.25]	-0.8 [-0.97 to -0.62]
Niger	651.31 [472.85-905.12]	1762.57 [1205.06-2480.76]	170.62	35.16 [25.74-48.01]	33.98 [23.54-46.95]	-0.26 [-0.34 to -0.17]
Nigeria	4757.19 [3015.48-6786.53]	11256.08 [7300.9-16604.56]	136.61	20.13 [12.91-28.12]	18.23 [12.14-26.47]	-0.19 [-0.28 to -0.1]
North Macedonia	151.64 [131.82-198.48]	213.14 [152.43-286.29]	40.56	14.58 [12.71-19.21]	14.51 [10.49-19.37]	-0.6 [-1.08 to -0.13]
Northern Mariana Islands	6.55 [4.62-8.99]	9.74 [7.13-12.9]	48.70	46.19 [34.09-61.58]	36.17 [26.58-47.24]	-0.89 [-1.04 to -0.74]
Norway	338.6 [312.69-364.54]	289.87 [224.42-371.04]	−14.39	12.53 [11.33-13.52]	8.04 [6.16-10.38]	-1.58 [-1.64 to -1.51]
Oman	29.09 [19.85-40.24]	63.05 [45.36-81.89]	116.74	7.73 [5.28-10.54]	5.94 [4.46-7.43]	-0.65 [-1 to -0.3]
Pakistan	2195.77 [1758.51-2774.01]	5698.99 [4024.8-8109.41]	159.54	6.97 [5.63-8.84]	7.7 [5.51-10.8]	0.09 [-0.15 to 0.33]
Palestine	31.28 [19.9-42.39]	66.94 [43.88-83.05]	114.00	5.95 [3.84-8]	4.66 [2.97-5.72]	-0.88 [-1.22 to -0.54]
Panama	325.76 [246.77-358.96]	475.56 [345.32-636.35]	45.98	36.87 [28.26-40.35]	22.52 [16.33-30.15]	-1.96 [-2.26 to -1.67]
Papua New Guinea	264.26 [155.83-416.02]	777.1 [438.85-1157.82]	194.07	22.17 [13.33-36.04]	23.52 [13.91-36.42]	0.32 [0.25 to 0.38]
Paraguay	447.82 [347.48-529.72]	1089.78 [751.75-1488.01]	143.35	33.09 [25.65-39.06]	34.26 [23.77-46.57]	-0.22 [-0.47 to 0.03]
Peru	2218.9 [1833.2-2764.41]	4777.12 [3269.21-6546.43]	115.29	31.09 [25.82-38.41]	27.64 [18.91-37.77]	-0.62 [-0.85 to -0.39]

Nation	Incident cases No.(95% UI)		change absolute number(%)	ASIR per 100,000 No.(95% UI)		1990-2019 EAPC No. (95% CI)
	1990	2019		1990	2019	
Philippines	3358.14 [2472.84-3983.65]	6775.69 [4723.14-9141.36]	101.77	16.44 [12.72-19.81]	13.9 [9.78-18.89]	-0.59 [-0.79 to -0.39]
Poland	4184.41 [3828.89-4382.95]	3252 [2447.19-4195.29]	−22.28	18 [16.5-18.88]	10.53 [7.88-13.77]	-2.1 [-2.29 to -1.91]
Portugal	1018.75 [916.57-1114.99]	936.36 [696.69-1225.7]	−8.09	15.79 [14.05-17.36]	10.37 [7.61-13.77]	-1.76 [-2.03 to -1.49]
Puerto Rico	214.85 [192.88-238.24]	275.65 [205.22-365.4]	28.30	11.2 [10.03-12.43]	10.86 [7.82-14.73]	-0.01 [-0.16 to 0.14]
Qatar	6.23 [4.54-8.73]	33.14 [23.37-46.8]	431.94	9.47 [7.14-12.92]	8.34 [6.26-11.04]	-0.08 [-0.37 to 0.2]
Republic of Korea	3109.97 [2587.85-3641.45]	3596.92 [2787.09-4661.05]	15.66	15.02 [12.84-18.23]	9.08 [6.98-11.85]	-2.34 [-2.6 to -2.08]
Republic of Moldova	527.57 [444.35-575.11]	398.54 [315.4-492.78]	−24.46	20.71 [17.37-22.58]	14.77 [11.43-18.42]	-0.51 [-0.8 to -0.21]
Romania	3965.54 [3672.73-4242.59]	3931.71 [2736.16-4965.29]	−0.85	29.28 [27.1-31.47]	27.36 [19.1-34.98]	-0.53 [-0.83 to -0.23]
Russian Federation	12438.13 [11730.45-13759.05]	16516.85 [12330.12-21065.03]	32.79	12.1 [11.38-13.49]	15.66 [11.27-20.22]	1.02 [0.78 to 1.27]
Rwanda	1092.9 [733.29-1506.83]	1421.32 [921.71-2198.51]	30.05	54.02 [37.19-73.18]	32.39 [21.4-48.8]	-2.53 [-2.87 to -2.19]
Saint Lucia	20.92 [18.46-23.34]	31.25 [25.2-38.3]	49.38	42.02 [37.16-46.67]	28.52 [22.96-34.91]	-1.64 [-1.87 to -1.41]
Saint Vincent and the Grenadines	19.82 [17.41-22.33]	26.09 [21.41-31.42]	31.63	50.46 [44.23-56.94]	41.01 [33.53-49.57]	-1.04 [-1.22 to -0.87]
Samoa	12.96 [8.42-17.75]	21.03 [12.55-30.69]	62.27	26.06 [17.18-35.45]	25.68 [15.39-37.17]	-0.12 [-0.19 to -0.05]
Sao Tome and Principe	14.51 [9.94-18.27]	31.01 [21.49-43.19]	113.71	40.54 [28.31-50.67]	44.01 [30.62-59.89]	-0.05 [-0.32 to 0.22]
Saudi Arabia	115.23 [77.82-194.8]	637.92 [440.02-920.2]	453.61	3.65 [2.57-6.11]	4.95 [3.65-6.79]	1.59 [1.3 to 1.88]
Senegal	634.27 [463.34-853.11]	1408.53 [992.86-1894.26]	122.07	31.6 [23.39-41.9]	29.88 [21.46-39.78]	0.01 [-0.2 to 0.23]
Serbia	1445.84 [1160.7-1695.22]	1370.31 [998.37-1797.79]	−5.22	25.62 [20.59-30.07]	22.27 [16.09-29.46]	-0.86 [-1.11 to -0.62]
Seychelles	12.07 [10.12-14.56]	19.55 [15.31-25.76]	61.97	40.81 [34.17-49.11]	33.51 [26.38-44.12]	-0.55 [-0.62 to -0.47]
Sierra Leone	303.49 [212.51-419.06]	803.56 [515.45-1145.63]	164.77	27.64 [19.64-37.83]	34 [22.22-48.48]	1.09 [0.88 to 1.29]

Nation	Incident cases No.(95% UI)		change absolute number(%)	ASIR per 100,000 No.(95% UI)		1990-2019 EAPC No. (95% CI)
	1990	2019		1990	2019	
Singapore	277.81 [236.92-308.38]	305.52 [235.59-399.63]	9.97	19.08 [16.31-21.03]	7.7 [5.91-10.05]	-3.4 [-3.62 to -3.17]
Slovakia	574.99 [483.33-656.78]	664.62 [427.8-881.36]	15.59	18.77 [15.76-21.46]	16.72 [10.72-22.36]	-0.17 [-0.37 to 0.03]
Slovenia	210.61 [153.87-282.76]	158.33 [114.43-220.51]	−24.82	16.88 [12.19-22.76]	10.03 [7.11-14.34]	-2.06 [-2.24 to -1.88]
Solomon Islands	49.27 [20.2-82.28]	133.42 [53.01-207.06]	170.79	55.51 [25.27-93.57]	57 [25.78-86.4]	0.15 [0.08 to 0.22]
Somalia	946.82 [546.06-1438.16]	2131.22 [1168.31-3320.29]	125.09	47.7 [27.23-70.63]	42.18 [23.88-65.21]	-0.25 [-0.31 to -0.18]
South Africa	4547.07 [3458.6-5681.61]	8246.15 [6613.55-10163.17]	81.35	31.75 [23.92-40.01]	29.19 [23.67-35.87]	-0.04 [-0.3 to 0.21]
South Sudan	422.83 [261.55-619.47]	659.37 [385.27-1103.74]	55.94	31.87 [19.88-45.37]	25.59 [15.57-41.43]	-0.72 [-0.85 to -0.6]
Spain	2597.29 [2214.4-2813.76]	3262.65 [2217.44-4293.21]	25.62	10.66 [8.96-11.6]	8.7 [5.98-11.52]	-0.61 [-0.75 to -0.48]
Sri Lanka	510.98 [427.3-692.09]	1077.09 [728.56-1498.8]	110.79	7.69 [6.48-10.6]	7.84 [5.34-10.94]	0.18 [-0.06 to 0.42]
Sudan	363.6 [163.22-505.7]	662.94 [373.04-997.9]	82.33	6.63 [2.97-9.08]	5.49 [3.2-8.02]	-0.66 [-0.73 to -0.59]
Suriname	51.82 [41.39-59.91]	104.72 [80.34-134.37]	102.08	34.36 [27.68-39.63]	32.61 [24.95-41.91]	-0.44 [-0.69 to -0.2]
Sweden	570.47 [521.33-657.3]	496.28 [387.28-625.16]	−13.01	9.8 [8.9-11.18]	6.93 [5.34-8.8]	-1.11 [-1.21 to -1]
Switzerland	494.73 [372.86-545]	392.87 [298.13-517.18]	−20.59	10.4 [8.07-11.57]	5.73 [4.26-7.62]	-2.21 [-2.31 to -2.1]
Syrian Arab Republic	125.96 [86.71-169.76]	219.43 [155.04-313.03]	74.21	3.87 [2.74-5.11]	3.25 [2.33-4.61]	-0.74 [-0.94 to -0.54]
Tajikistan	215.32 [152.39-249.66]	263.46 [194.74-432.4]	22.36	13.01 [9.13-15.05]	7.65 [5.82-11.86]	-1.86 [-2.37 to -1.35]
Thailand	6504.62 [4705.09-7765.53]	8335.44 [5835.18-12253.11]	28.15	27.56 [20.26-32.65]	16.27 [11.26-24.12]	-2.46 [-2.82 to -2.1]
Timor-Leste	38.23 [20.77-55.26]	68.81 [40.13-105.09]	79.99	18.49 [10.81-27.16]	15.52 [9.4-23.67]	-0.78 [-1.11 to -0.45]
Togo	332.33 [255.35-444.49]	840.46 [581.06-1171.62]	152.90	35.77 [28.09-47.67]	30.94 [21.92-42.44]	-0.41 [-0.5 to -0.31]
Tonga	11.92 [9.16-15.04]	13.76 [9.83-19.13]	15.44	37.28 [28.73-46.48]	31.45 [22.53-43.86]	-0.72 [-0.88 to -0.57]

Nation	Incident cases No.(95% UI)		change absolute number(%)	ASIR per 100,000 No.(95% UI)		1990-2019 EAPC No. (95% CI)
	1990	2019		1990	2019	
Trinidad and Tobago	142.86 [129.97-162.06]	200.62 [144.78-270.96]	40.43	30.23 [27.49-33.7]	22.28 [16.03-30.26]	-1.41 [-1.59 to -1.22]
Tunisia	185.09 [132.72-230.97]	388.83 [254.7-544.41]	110.08	6.43 [4.7-7.99]	5.72 [3.79-7.98]	-0.46 [-0.51 to -0.41]
Turkey	1458.5 [914.31-1840.91]	2201.82 [1528.53-2827.72]	50.96	6.91 [4.39-8.65]	4.67 [3.23-5.99]	-1.34 [-1.67 to -1]
Turkmenistan	183.54 [165.57-203.12]	389.62 [276.16-521.47]	112.28	14.79 [13.27-16.35]	15.59 [11.08-20.66]	1.03 [0.61 to 1.46]
Uganda	1384.88 [948.08-1891.98]	3996.54 [2791.48-5393.56]	188.58	33.39 [23.39-44.86]	37.92 [26.74-49.75]	0.12 [-0.13 to 0.36]
Ukraine	7737.16 [5212.86-8452.92]	4354.81 [3239.37-5878.71]	−43.72	19.66 [13.26-21.51]	11.91 [8.76-16.42]	-2.32 [-2.54 to -2.1]
United Arab Emirates	31.55 [22.35-44.19]	189.67 [130.27-283]	501.17	16.83 [11.34-23.71]	10.82 [7.66-14.68]	-1.4 [-2.06 to -0.74]
United Kingdom	5194.05 [4374.59-5373.55]	3668.82 [2811.18-4814.18]	−29.36	13.97 [10.98-14.48]	8.19 [6.19-10.71]	-1.71 [-2 to -1.43]
United Republic of Tanzania	2748.52 [1859.08-3736.55]	5894.98 [3874.41-8557.86]	114.48	39.53 [26.76-53.08]	35.17 [23.77-49.46]	-0.34 [-0.49 to -0.18]
United States of America	15655.91 [13227.88-16361.49]	19111.68 [14884.97-23573.48]	22.07	10.28 [8.59-10.73]	8.67 [6.7-10.73]	-0.68 [-0.83 to -0.54]
United States Virgin Islands	11.44 [8.85-13.97]	12.8 [9.63-16.4]	11.89	21.53 [16.77-26.18]	15.46 [11.43-20.33]	-1.17 [-1.27 to -1.08]
Uruguay	419.92 [380.7-466.19]	482.22 [357.26-631.47]	14.84	23.84 [21.49-26.51]	21.81 [15.92-28.93]	-0.47 [-0.63 to -0.3]
Uzbekistan	1071.41 [958.37-1199.6]	2704.05 [2101.48-3346.58]	152.38	15.26 [13.69-16.92]	17.26 [13.49-21.22]	0.17 [-0.06 to 0.4]
Vanuatu	10.64 [5.64-16.42]	29.34 [15.46-46.51]	175.75	27.06 [15.08-41.78]	28.7 [15.49-44.39]	-0.23 [-0.47 to 0.01]
Venezuela (Bolivarian Republic of)	2220.83 [2048.93-2427.15]	5428.43 [3862.72-7463.5]	144.43	34.11 [31.52-37.53]	34.36 [24.47-47.24]	-0.31 [-0.53 to -0.1]
Viet Nam	4250.27 [3146.87-5548.01]	9966.02 [6955.55-13157.58]	134.48	17.58 [13.09-23.22]	17.77 [12.52-23.37]	0.03 [-0.09 to 0.16]
Yemen	214.01 [95.62-331.68]	573.11 [340.7-871.27]	167.80	6.92 [3.27-10.42]	6.39 [3.98-9.4]	-0.29 [-0.37 to -0.21]

Nation	Incident cases No.(95% UI)		change absolute number(%)	ASIR per 100,000 No.(95% UI)		1990-2019 EAPC No. (95% CI)
	1990	2019		1990	2019	
Zambia	1022.98 [708.43-1353.96]	2189.25 [1436.43-3097.98]	114.01	52.18 [36.49-67.98]	43.37 [28.91-60.93]	-1.03 [-1.2 to -0.87]
Zimbabwe	1156.87 [811.14-1496.36]	2448.9 [1631.82-3410.78]	111.68	45.33 [31.58-58.26]	48.95 [33.04-67.77]	0.85 [0.5 to 1.2]

Supplementary Table S4. The death cases and age-standardized death rate of Cervical cancer in 1990 and 2019, and its temporal trends from 1990 to 2019.

Nation	Death Cases No.(95% UI)		change absolute number(%)	ASDR per 100,000 No.(95% UI)		1990-2019 EAPC No. (95% CI)
	1990	2019		1990	2019	
Afghanistan	332.23 [111.15-507.53]	608.59 [238.82-938.83]	83.18	8.96 [3.18-13.47]	7.59 [3.4-11.25]	-0.71 [-0.9 to -0.52]
Albania	44.74 [39.44-64.08]	64.03 [44.95-87.93]	43.12	3.85 [3.38-5.63]	3.13 [2.22-4.32]	-0.45 [-0.65 to -0.26]
Algeria	494.46 [343.71-641.01]	786.67 [561.78-1061.63]	59.10	7.61 [5.56-9.73]	4.52 [3.3-5.95]	-1.73 [-1.86 to -1.59]
American Samoa	1.16 [0.94-1.57]	2.39 [1.8-3.07]	106.03	9.75 [7.97-13.11]	9.59 [7.22-12.23]	0.04 [-0.16 to 0.24]
Andorra	1.4 [1-1.98]	2.85 [2.02-3.88]	103.57	5.22 [3.75-7.35]	4.1 [2.9-5.64]	-0.87 [-1.1 to -0.63]
Angola	635.86 [418.93-920.99]	1460.94 [928.2-2148.17]	129.76	26.12 [17.31-37.21]	19.85 [13.12-29.7]	-1.11 [-1.26 to -0.95]
Antigua and Barbuda	3.48 [3.03-4.01]	5.89 [4.81-7.13]	69.25	11.95 [10.43-13.75]	10.81 [8.89-12.98]	-0.6 [-0.77 to -0.42]
Argentina	1853.37 [1734.82-2181.56]	3034.8 [2408.9-3353.53]	63.74	10.85 [10.15-12.7]	10.85 [8.52-11.96]	-0.11 [-0.29 to 0.07]
Armenia	152.22 [139.77-168.3]	158.02 [127.07-191.35]	3.81	9.55 [8.77-10.55]	7.09 [5.72-8.59]	-1.03 [-1.26 to -0.8]
Australia	344.92 [312.53-364.55]	436.38 [363.5-487.92]	26.52	3.37 [3.03-3.57]	2.14 [1.8-2.37]	-1.27 [-1.66 to -0.88]
Austria	428.33 [356.69-459.45]	239.32 [211.51-302.17]	−44.13	6.56 [5.43-7.01]	2.66 [2.37-3.36]	-3.26 [-3.5 to -3.03]
Azerbaijan	225.22 [195.45-281.62]	333.5 [248.15-477.24]	48.08	7.44 [6.43-9.42]	6.04 [4.56-8.72]	-0.79 [-0.95 to -0.64]
Bahamas	13.75 [12.03-15.56]	24.07 [18.77-30.66]	75.05	14.31 [12.51-16.19]	10.85 [8.48-13.77]	-1.08 [-1.19 to -0.98]
Bahrain	4.31 [3.51-5.58]	11.46 [8.71-15.51]	165.89	4.98 [4.06-6.6]	2.96 [2.3-3.99]	-1.89 [-2.1 to -1.68]
Bangladesh	3243.86 [1468.88-4235.01]	3864.42 [2149.88-5840.58]	19.13	12.99 [5.87-16.83]	5.67 [3.14-8.52]	-2.69 [-2.89 to -2.48]
Barbados	26.71 [23.89-29.26]	32.83 [26.54-40.08]	22.91	17.22 [15.41-18.83]	13.11 [10.57-16.04]	-0.82 [-0.9 to -0.73]
Belarus	635.69 [590.14-697.46]	507.31 [382.23-692.94]	−20.20	8.35 [7.77-9.12]	5.9 [4.41-8.08]	-1.57 [-1.76 to -1.39]
Belgium	369.42 [301.93-397.81]	306.4 [257.24-342.01]	−17.06	4.52 [3.79-4.84]	2.69 [2.31-2.99]	-1.82 [-2.01 to -1.63]
Belize	9.83 [8.68-11.14]	27.75 [22.77-32.78]	182.30	20.28 [17.89-22.83]	17.66 [14.59-20.77]	-0.64 [-0.99 to -0.28]
Benin	259.03 [206.37-338.95]	593.7 [425.51-828.42]	129.20	22.62 [18.1-29.48]	20.12 [14.87-27.41]	-0.3 [-0.35 to -0.25]

Nation	Death Cases No.(95% UI)		change absolute number(%)	ASDR per 100,000 No.(95% UI)		1990-2019 EAPC No. (95% CI)
	1990	2019		1990	2019	
Bermuda	2.73 [2.36-3.12]	2.17 [1.73-2.75]	−20.51	7.75 [6.72-8.86]	3.25 [2.59-4.1]	-3.56 [-3.83 to -3.29]
Bhutan	19.86 [9.18-28.03]	21.07 [12.53-32.37]	6.09	13.55 [6.25-18.92]	7.24 [4.35-11.01]	-2.38 [-2.63 to -2.13]
Bolivia (Plurinational State of)	616.01 [399.87-777.39]	1137.89 [810.75-1544.87]	84.72	33.21 [21.89-41.64]	24.14 [17.53-32.4]	-1.25 [-1.37 to -1.13]
Bosnia and Herzegovina	150.85 [132.13-179.75]	163.01 [115.52-209.21]	8.06	6.35 [5.62-7.64]	5.61 [3.89-7.24]	-0.55 [-0.73 to -0.38]
Botswana	83.75 [54.35-126.02]	230.79 [137.33-359.37]	175.57	24.57 [16.15-35.97]	26.7 [16.4-40.82]	0.23 [-0.05 to 0.51]
Brazil	7448.47 [6986.32-8582.9]	11074.54 [10242.19-13171.71]	48.68	14.63 [13.63-16.94]	8.51 [7.87-10.1]	-2.06 [-2.16 to -1.97]
Brunei Darussalam	10.54 [7.85-13.06]	18.31 [14.79-23.58]	73.72	17.51 [13.31-21.37]	10.46 [8.59-13.36]	-1.79 [-1.94 to -1.65]
Bulgaria	452.62 [409.71-512.73]	509.58 [368.69-661.5]	12.58	7.47 [6.73-8.42]	8.25 [5.75-10.78]	1.2 [0.9 to 1.49]
Burkina Faso	653.1 [477.6-872.81]	1311.42 [959.17-1722.99]	100.80	25.67 [19.07-33.96]	22.76 [17.05-29.16]	-0.44 [-0.62 to -0.27]
Burundi	504.07 [328.35-702.1]	680.8 [419.32-1013.72]	35.06	34.64 [22.86-47.71]	25.34 [15.7-37.38]	-1.56 [-1.74 to -1.37]
Cabo Verde	24.69 [20.14-29.76]	34.06 [27.2-46.07]	37.95	19 [15.74-22.96]	13.86 [11.13-18.73]	-0.98 [-1.33 to -0.63]
Cambodia	437.37 [243.61-628.1]	708.89 [508.29-1095.94]	62.08	14.66 [8.29-21.33]	9.67 [7.05-15.15]	-1.59 [-1.69 to -1.48]
Cameroon	628.02 [492.9-833.35]	1489.09 [958.82-2231.92]	137.11	24.01 [19-31.94]	20.87 [13.88-30.38]	-0.38 [-0.55 to -0.2]
Canada	536.28 [493.27-568.74]	800.79 [660.63-889.52]	49.32	3.09 [2.87-3.27]	2.44 [2.04-2.7]	-0.64 [-0.78 to -0.49]
Central African Republic	252.16 [162.96-344.27]	420.82 [251.57-627.76]	66.89	33.74 [22.56-45.49]	29.31 [18.31-43.24]	-0.57 [-0.71 to -0.43]
Chad	376.85 [290.1-524.25]	764.31 [528.07-1027.71]	102.82	23.88 [18.33-33.13]	25.08 [17.7-33.2]	0.34 [0.21 to 0.47]
Chile	1007.69 [841.48-1069.42]	911.3 [814.06-1035.59]	−9.57	17.97 [14.88-19.11]	7.19 [6.42-8.29]	-3.46 [-3.64 to -3.27]
China	26419.82 [20524.28-43527.4]	53441.4 [30398.79-68856.44]	102.28	5.85 [4.59-9.57]	5.13 [2.92-6.6]	0.09 [-0.18 to 0.36]

Nation	Death Cases No.(95% UI)		change absolute number(%)	ASDR per 100,000 No.(95% UI)		1990-2019 EAPC No. (95% CI)
	1990	2019		1990	2019	
Colombia	1612.05 [1502.01-1789.54]	2395.02 [1838.53-3120.04]	48.57	16.09 [14.99-18.07]	8.41 [6.41-10.99]	-2.73 [-2.96 to -2.49]
Comoros	32.69 [15.08-49.17]	62.09 [40.02-90.55]	89.94	26.29 [13.03-39.14]	22.17 [14.41-32.16]	-0.79 [-0.98 to -0.6]
Congo	216.5 [145.33-287.68]	385.79 [243.43-556.38]	78.19	32.87 [22.37-43.44]	23.8 [15.54-33.42]	-1.18 [-1.36 to -1]
Costa Rica	144.39 [124.83-156.14]	201.19 [153.12-267.31]	39.34	14.99 [12.91-16.22]	7.22 [5.47-9.59]	-3.12 [-3.51 to -2.73]
Cote d'Ivoire	502.36 [379.81-660.87]	1140.22 [775.94-1591.42]	126.97	21.56 [16.83-27.77]	18.94 [13.18-25.96]	-0.24 [-0.36 to -0.12]
Croatia	253.63 [200.09-280.64]	164 [122.59-211.92]	−35.34	7.1 [5.67-7.86]	3.68 [2.77-4.75]	-2.11 [-2.48 to -1.73]
Cuba	570.55 [507.86-612.53]	702.41 [548.65-869.74]	23.11	10.86 [9.66-11.65]	7.6 [5.89-9.39]	-1.28 [-1.37 to -1.2]
Cyprus	17.26 [13.51-23.75]	28.61 [18.86-33.84]	65.76	4.27 [3.32-5.92]	2.89 [1.93-3.41]	-1.17 [-1.38 to -0.96]
Czechia	638.62 [595.08-676.12]	443.75 [357.03-543.4]	−30.51	8.61 [8.06-9.12]	4.35 [3.52-5.41]	-2.4 [-2.49 to -2.31]
Democratic People's Republic of Korea	972.54 [668.25-1677.31]	1486.09 [982.38-2247.93]	52.81	9.41 [6.55-16.44]	8.28 [5.42-12.52]	-0.26 [-0.38 to -0.15]
Democratic Republic of the Congo	2506.24 [1698.07-3345.77]	4872.35 [3145.16-6858.51]	94.41	25.35 [17.41-34.49]	21.84 [14.12-31.24]	-0.45 [-0.58 to -0.33]
Denmark	353.43 [273.41-374.31]	181.75 [160.11-245.96]	−48.58	8.72 [6.76-9.24]	3.28 [2.92-4.5]	-3.51 [-3.84 to -3.18]
Djibouti	22.39 [13.62-33.22]	75.09 [43.19-132.82]	235.37	24.92 [15.53-35.73]	21.77 [13-36.51]	-0.52 [-0.65 to -0.4]
Dominica	8.26 [6.93-9.82]	7.52 [5.82-9.55]	−8.96	22.15 [18.32-26.88]	17.33 [13.33-22.01]	-0.86 [-0.93 to -0.78]
Dominican Republic	256.19 [216.06-329.12]	658.27 [476.32-915.44]	156.95	12.29 [10.4-15.26]	13.31 [9.61-18.49]	0.51 [0.38 to 0.64]
Ecuador	516.18 [439.32-596.06]	1056.68 [794.65-1397.6]	104.71	17.73 [15.08-20.4]	13.3 [10-17.5]	-0.79 [-1.02 to -0.56]
Egypt	297.14 [254.65-391.73]	503.13 [340.04-732.57]	69.32	1.97 [1.68-2.6]	1.77 [1.2-2.55]	-0.03 [-0.14 to 0.09]
El Salvador	290.4 [260.85-377.1]	522.81 [377.02-694.14]	80.03	17.17 [15.43-22.43]	15.26 [10.99-20.32]	-1.07 [-1.49 to -0.64]

Nation	Death Cases No.(95% UI)		change absolute number(%)	ASDR per 100,000 No.(95% UI)		1990-2019 EAPC No. (95% CI)
	1990	2019		1990	2019	
Equatorial Guinea	34.44 [20.09-50.22]	54.31 [32.88-89.58]	57.69	27.31 [16.07-39.74]	17.09 [10.9-26.96]	-1.76 [-1.92 to -1.61]
Eritrea	225.92 [138.8-348.68]	550.92 [347.59-805.4]	143.86	30.94 [18.72-47.65]	30.26 [19.35-43.43]	-0.01 [-0.09 to 0.08]
Estonia	118.09 [101.59-127.28]	68.3 [51.96-87.72]	−42.16	9.8 [8.47-10.57]	5.08 [3.77-6.59]	-2.61 [-2.79 to -2.42]
Eswatini	42.79 [27.87-59.92]	97.39 [50.14-165.74]	127.60	23.24 [15.27-32.38]	26.85 [13.98-45.7]	1.07 [0.39 to 1.75]
Ethiopia	3255.82 [1678.3-4991.26]	3869.58 [2677.55-6286.13]	18.85	27.4 [15.34-41.2]	16.82 [11.73-27.12]	-2.08 [-2.27 to -1.89]
Fiji	57.88 [28.53-76.19]	89.55 [37.2-123.1]	54.72	26.79 [13.39-34.7]	22.1 [9.26-29.74]	-0.25 [-0.56 to 0.07]
Finland	103.73 [92.61-111.92]	113.17 [77.19-129.18]	9.10	2.47 [2.26-2.68]	1.78 [1.33-2.02]	-0.77 [-0.99 to -0.54]
France	2048.74 [1693.84-2187.44]	1856.95 [1550.95-2093.17]	−9.36	4.68 [3.89-4.97]	2.75 [2.37-3.08]	-1.73 [-1.9 to -1.56]
Gabon	75.57 [53.04-101.9]	102.08 [66.15-148.49]	35.08	23.98 [16.75-32.29]	16.73 [11.04-24.33]	-1.35 [-1.62 to -1.08]
Gambia	28.98 [19.62-39.85]	93.56 [65.32-129.07]	222.84	14.94 [10.36-20.13]	16.69 [11.73-22.67]	0.2 [-0.02 to 0.42]
Germany	3341.12 [3057.6-3578.28]	2609.47 [2303.28-2894.66]	−21.90	4.98 [4.61-5.26]	2.87 [2.57-3.16]	-2 [-2.16 to -1.85]
Ghana	869.74 [661.22-1183.56]	1716.63 [1159.4-2341.8]	97.37	22.54 [17.16-30.77]	16.94 [11.55-22.77]	-1.04 [-1.12 to -0.96]
Greece	358.92 [328.09-385.75]	342.73 [302.49-389.43]	−4.51	4.66 [4.21-5.02]	2.96 [2.67-3.35]	-1.63 [-1.83 to -1.43]
Greenland	3.38 [2.72-4.14]	2.93 [2.24-3.85]	−13.31	17.22 [13.88-20.95]	9.1 [6.99-11.85]	-2.77 [-3.01 to -2.52]
Grenada	8.54 [7.47-9.75]	9.81 [8.08-11.31]	14.87	22.73 [19.86-26.07]	17 [13.97-19.59]	-0.86 [-1.17 to -0.55]
Guam	3.1 [2.52-3.81]	5.67 [4.36-7.07]	82.90	8.39 [6.75-10.22]	5.94 [4.57-7.43]	-1.44 [-1.92 to -0.96]
Guatemala	317.49 [260.01-553.37]	1194.93 [834.19-1554.9]	276.37	15.4 [12.83-26.22]	18.44 [12.71-23.79]	0.61 [0.11 to 1.12]
Guinea	762.24 [584.12-958.25]	1148.75 [824.36-1527.85]	50.71	42.09 [32.63-53.22]	36.16 [26.43-47.56]	-0.39 [-0.44 to -0.34]
Guinea-Bissau	82.34 [53.85-117.02]	140.91 [88.24-197.59]	71.13	32.43 [21.5-46.02]	29.28 [18.87-40.39]	-0.14 [-0.25 to -0.04]
Guyana	63.56 [51.85-76.26]	74.45 [55.9-98.17]	17.13	28.31 [23.24-33.82]	21.01 [15.93-27.5]	-1.05 [-1.21 to -0.89]
Haiti	779.76 [329.88-1044.06]	1232.59 [566.03-1831.57]	58.07	39.7 [17.45-51.88]	27.76 [12.82-40.33]	-1.12 [-1.21 to -1.04]

Nation	Death Cases No.(95% UI)		change absolute number(%)	ASDR per 100,000 No.(95% UI)		1990-2019 EAPC No. (95% CI)
	1990	2019		1990	2019	
Honduras	150 [115.93-193.07]	387.79 [246.55-591.32]	158.53	11.73 [9.04-15.25]	11.14 [7.25-16.71]	-0.21 [-0.39 to -0.04]
Hungary	680.76 [638.83-731.74]	481.27 [386.71-597.74]	−29.30	8.79 [8.24-9.49]	5.18 [4.13-6.51]	-1.99 [-2.15 to -1.83]
Iceland	5.74 [5.03-6.41]	5.2 [4.25-6.1]	−9.41	3.96 [3.46-4.43]	1.9 [1.58-2.23]	-2.74 [-2.95 to -2.52]
India	27896.8 [22139.81-35328.05]	45446.63 [35004.18-62351.78]	62.91	10.9 [8.59-13.74]	7.38 [5.71-10.13]	-1.6 [-1.81 to -1.39]
Indonesia	5743.12 [3520.01-8697.13]	8703.43 [5768.88-14505.65]	51.55	9.66 [6.01-14.9]	7.08 [4.77-12.05]	-0.98 [-1.1 to -0.86]
Iran (Islamic Republic of)	416.06 [297.35-500.81]	761.14 [551.75-874.4]	82.94	3.22 [2.33-4.05]	2.06 [1.49-2.37]	-1.64 [-1.85 to -1.43]
Iraq	120.95 [88.74-168.1]	306.47 [218.69-414.73]	153.39	2.88 [2.12-4.04]	2.4 [1.72-3.18]	-0.71 [-0.85 to -0.57]
Ireland	86.18 [79.42-97.46]	101.48 [82.16-117.46]	17.75	4.25 [3.9-4.69]	2.81 [2.2-3.26]	-1.18 [-1.31 to -1.05]
Israel	76.06 [69.47-92.6]	150.41 [117.67-169.98]	97.75	3.01 [2.75-3.65]	2.53 [1.99-2.85]	-0.79 [-0.91 to -0.68]
Italy	906.45 [852.38-1108.52]	1589.35 [970.15-1770.64]	75.34	1.94 [1.83-2.36]	2.23 [1.38-2.47]	1.04 [0.78 to 1.3]
Jamaica	161.88 [138.6-174.86]	246.54 [186.64-315.38]	52.30	17.66 [15.07-19.07]	15.9 [11.93-20.5]	-0.44 [-0.76 to -0.11]
Japan	3393.98 [3110.77-3735.39]	4210.49 [3348.02-4688.09]	24.06	3.66 [3.37-4.06]	2.78 [2.18-3.04]	-0.67 [-0.81 to -0.53]
Jordan	26.49 [20.5-33.82]	66.54 [48.33-89.13]	151.19	3.66 [2.84-4.79]	2.04 [1.52-2.74]	-2.34 [-2.59 to -2.09]
Kazakhstan	893.09 [803.67-977.29]	792.7 [667.39-964.41]	−11.24	11.49 [10.37-12.56]	7.65 [6.44-9.24]	-1.05 [-1.41 to -0.7]
Kenya	595.33 [400.02-962.29]	1724.99 [1160.77-2798.6]	189.75	12.55 [8.53-19.92]	12.92 [8.82-20.82]	0.38 [0.2 to 0.55]
Kuwait	9.06 [7.4-10.63]	20.78 [15.59-29.33]	129.36	3.19 [2.51-3.76]	1.76 [1.3-2.41]	-1.4 [-1.73 to -1.06]
Kyrgyzstan	224.51 [187.62-243.67]	252.05 [202.73-297.99]	12.27	12.66 [10.65-13.76]	8.94 [7.19-10.54]	-1.14 [-1.35 to -0.93]
Lao People's Democratic Republic	210.84 [113.94-319.57]	233.8 [144.58-359.09]	10.89	16.93 [9.36-26.11]	8.98 [5.53-14.29]	-2.43 [-2.53 to -2.34]
Latvia	176.35 [147.94-190.7]	94.52 [70.91-127.96]	−46.40	8.32 [7.1-9]	4.46 [3.29-6.09]	-2.21 [-2.47 to -1.95]

Nation	Death Cases No.(95% UI)		change absolute number(%)	ASDR per 100,000 No.(95% UI)		1990-2019 EAPC No. (95% CI)
	1990	2019		1990	2019	
Lebanon	47.14 [35.06-59.37]	69.69 [50.61-97.19]	47.84	3.94 [3-4.95]	2.43 [1.77-3.39]	-1.76 [-1.82 to -1.7]
Lesotho	113.58 [75.93-165.7]	277.69 [139.56-475.41]	144.49	20.16 [13.48-29.14]	35.96 [18.42-60.81]	3.25 [2.7 to 3.8]
Liberia	135.03 [103.32-178.33]	244.02 [161.81-341.51]	80.72	24.48 [18.96-32.19]	20.49 [13.88-28.24]	-0.62 [-0.73 to -0.51]
Libya	46.87 [32.33-60.79]	115.92 [78.84-155.57]	147.32	4.95 [3.46-6.37]	4.02 [2.82-5.28]	-0.57 [-0.82 to -0.31]
Lithuania	243.76 [201.9-262.61]	163.7 [127.43-201.61]	−32.84	9.39 [7.85-10.11]	5.41 [4.22-6.7]	-1.81 [-2.02 to -1.61]
Luxembourg	12.57 [11.1-13.83]	10 [8.38-12.45]	−20.45	4.3 [3.78-4.72]	1.94 [1.63-2.42]	-2.66 [-2.82 to -2.5]
Madagascar	800.72 [551.65-1057.47]	1573.74 [1010.02-2289.87]	96.54	26 [17.73-33.81]	21.87 [14.01-31.45]	-0.69 [-0.77 to -0.62]
Malawi	712.7 [517.83-948.69]	1184.79 [780.8-1713.97]	66.24	29.95 [21.85-39.88]	25.61 [17.41-35.46]	-0.65 [-0.86 to -0.43]
Malaysia	662.4 [436.26-761.07]	1203.52 [832.35-1578.24]	81.69	13.16 [8.53-15.15]	9 [6.25-11.73]	-1.76 [-2.03 to -1.49]
Maldives	5.98 [2.48-8.6]	5.96 [4.69-7.68]	−0.33	13.12 [5.71-18.24]	4.14 [3.25-5.28]	-4.54 [-4.87 to -4.21]
Mali	580.8 [437.2-720.18]	894.15 [620.6-1228.94]	53.95	24.3 [18.6-30.21]	18.42 [13.08-24.83]	-1.15 [-1.24 to -1.06]
Malta	7.9 [6.95-8.81]	8 [6.4-9.66]	1.27	3.39 [2.96-3.78]	1.78 [1.48-2.15]	-1.83 [-2.07 to -1.6]
Marshall Islands	2.13 [1.44-3.24]	4.32 [2.52-6.8]	102.82	22.73 [15.66-35.38]	21.42 [12.97-33.44]	-0.26 [-0.41 to -0.11]
Mauritania	156.29 [113.37-207.43]	201.93 [141.74-278.71]	29.20	27.94 [20.38-37.02]	18.07 [12.89-24.55]	-1.26 [-1.37 to -1.15]
Mauritius	40.92 [36.54-45.01]	53.58 [42.32-67.79]	30.94	9.97 [8.88-10.95]	5.76 [4.54-7.29]	-2.41 [-2.62 to -2.2]
Mexico	5558.15 [4534.23-5839.37]	6104.47 [4903.67-8120.16]	9.83	23.68 [18.62-24.98]	9.53 [7.68-12.64]	-3.55 [-3.75 to -3.34]
Micronesia (Federated States of)	6.24 [4.06-9.74]	7.85 [4.88-12.51]	25.80	24.59 [16.33-38.04]	19.94 [12.85-31.51]	-0.68 [-0.83 to -0.52]
Mongolia	109.94 [86.06-142.08]	175.49 [125.95-243]	59.62	18.7 [14.62-23.65]	12.2 [8.77-16.57]	-1.99 [-2.25 to -1.73]
Montenegro	17.49 [14.58-23.21]	23.18 [18.71-29.7]	32.53	5.12 [4.26-6.79]	4.84 [3.89-6.13]	-0.27 [-0.66 to 0.13]

Nation	Death Cases No.(95% UI)		change absolute number(%)	ASDR per 100,000 No.(95% UI)		1990-2019 EAPC No. (95% CI)
	1990	2019		1990	2019	
Morocco	685.33 [455.04-842.1]	1333.78 [873.88-1793.48]	94.62	9.12 [6.13-11.13]	7.83 [5.31-10.43]	-0.45 [-0.55 to -0.35]
Mozambique	1041.98 [683.18-1472.81]	2043.29 [1267.89-3028.95]	96.10	28.06 [18.5-39.66]	28.76 [18.43-41.69]	0.29 [0.03 to 0.55]
Myanmar	2090.4 [1211.59-3322.24]	2144.94 [1499.4-3611.85]	2.61	15.07 [8.95-24.35]	7.73 [5.48-13.24]	-2.57 [-2.79 to -2.36]
Namibia	57.81 [39.02-84.91]	139.95 [93.37-203.5]	142.09	14.26 [9.64-21.03]	16.38 [11.1-23.56]	0.67 [0.49 to 0.84]
Nepal	827.34 [384.8-1130.23]	1024.37 [637.84-1475.53]	23.81	14.83 [6.91-19.94]	8.15 [5.05-11.65]	-2.12 [-2.55 to -1.68]
Netherlands	342.22 [307.51-365.29]	373.21 [318.29-416.49]	9.06	3.19 [2.91-3.4]	2.25 [1.96-2.51]	-1.21 [-1.33 to -1.08]
New Zealand	109.87 [77.44-118.65]	88.22 [75.89-99.12]	−19.71	5.54 [3.84-6]	2.35 [2.06-2.65]	-2.73 [-3.16 to -2.31]
Nicaragua	206.79 [162.16-232.55]	420.13 [336.16-569.3]	103.17	21.76 [17.02-24.61]	16.74 [13.48-22.26]	-1.24 [-1.56 to -0.91]
Niger	411.72 [299.96-566.88]	1077.21 [743.68-1483.87]	161.64	25.33 [18.56-34.5]	23.62 [16.62-31.87]	-0.36 [-0.44 to -0.28]
Nigeria	3113.84 [2120.77-4536.81]	6436.13 [4282.56-9304.52]	106.69	14.07 [9.66-20.53]	12.08 [8.26-16.87]	-0.39 [-0.46 to -0.32]
North Macedonia	69.42 [60.06-95.48]	96.67 [70.93-127.81]	39.25	6.88 [5.96-9.57]	6.12 [4.48-8.12]	-0.94 [-1.4 to -0.47]
Northern Mariana Islands	2.13 [1.55-2.85]	4 [2.99-5.18]	87.79	21.67 [16.4-27.95]	15.64 [11.99-20.08]	-0.97 [-1.16 to -0.78]
Norway	163.64 [149.95-173.42]	131.44 [115.18-146.76]	−19.68	4.98 [4.65-5.24]	2.8 [2.52-3.12]	-2.02 [-2.14 to -1.91]
Oman	14.65 [9.97-19.85]	23.19 [17.36-28.86]	58.29	4.59 [3.16-6.34]	3.1 [2.37-3.8]	-0.94 [-1.19 to -0.7]
Pakistan	1349.35 [1086.58-1692.29]	2946.69 [2094.93-4139.3]	118.38	4.64 [3.69-5.77]	4.55 [3.23-6.28]	-0.35 [-0.62 to -0.07]
Palestine	18.55 [11.92-24.46]	34.19 [21.3-42.02]	84.31	3.91 [2.54-5.1]	2.87 [1.72-3.52]	-1.06 [-1.32 to -0.8]
Panama	160.74 [127.93-174.35]	214.56 [162.6-282.58]	33.48	19.76 [16-21.38]	10.02 [7.57-13.2]	-2.48 [-2.67 to -2.29]
Papua New Guinea	148.09 [88.19-245.23]	397.29 [229.27-601.62]	168.28	14.19 [8.84-23.91]	14.2 [8.49-22.27]	0.16 [0.08 to 0.25]
Paraguay	236.5 [184.83-279.38]	505.85 [362.42-675.1]	113.89	18.82 [14.87-22.27]	16.69 [12.02-22.19]	-0.66 [-0.96 to -0.37]
Peru	1198.94 [1002.31-1477.71]	2083.46 [1473.96-2804.94]	73.78	18.01 [15.11-22.03]	12.23 [8.68-16.51]	-1.59 [-1.82 to -1.36]

Nation	Death Cases No.(95% UI)		change absolute number(%)	ASDR per 100,000 No.(95% UI)		1990-2019 EAPC No. (95% CI)
	1990	2019		1990	2019	
Philippines	1608.3 [1325.79-1960.43]	3120.44 [2281.66-4237.57]	94.02	8.98 [7.59-11.29]	6.83 [5.03-9.25]	-0.9 [-1.11 to -0.69]
Poland	2795.57 [2565.61-2892.18]	2117.56 [1651.3-2683.18]	−24.25	11.54 [10.67-11.94]	5.95 [4.59-7.57]	-2.54 [-2.69 to -2.38]
Portugal	447.33 [407.86-483.22]	373.92 [327.51-423.74]	−16.41	6.24 [5.67-6.7]	3.17 [2.82-3.58]	-2.5 [-2.62 to -2.38]
Puerto Rico	101.87 [92.16-111.46]	123.71 [95-160.2]	21.44	5.27 [4.77-5.77]	3.77 [2.82-4.96]	-1.15 [-1.33 to -0.97]
Qatar	2.67 [2-3.74]	9.87 [7.22-13.69]	269.66	5.99 [4.51-8.33]	5.03 [3.83-6.62]	-0.18 [-0.48 to 0.13]
Republic of Korea	1140.21 [998.06-1479.71]	1269.67 [1009.87-1586.86]	11.35	6.04 [5.45-8.27]	2.72 [2.17-3.4]	-3.47 [-3.82 to -3.12]
Republic of Moldova	284.18 [252.16-307.02]	191.96 [157.59-237.93]	−32.45	10.97 [9.71-11.79]	6.36 [5.19-7.87]	-1.27 [-1.56 to -0.97]
Romania	1899.37 [1786.55-2112.88]	1887.26 [1370.96-2340.01]	−0.64	13.12 [12.37-14.51]	10.96 [7.71-13.76]	-0.79 [-1.04 to -0.54]
Russian Federation	7086.67 [6465.04-7723.28]	6845.21 [5305.06-8419.96]	−3.41	6.36 [5.86-6.98]	5.6 [4.22-6.95]	-0.6 [-0.83 to -0.36]
Rwanda	692.73 [477.64-936.21]	807.92 [536.18-1204.67]	16.63	37.11 [25.71-49.62]	20.62 [14.1-29.74]	-2.82 [-3.16 to -2.49]
Saint Lucia	11.31 [10.15-12.5]	15.21 [12.48-18.45]	34.48	23.41 [20.98-25.85]	13.5 [11.07-16.4]	-2.32 [-2.62 to -2.01]
Saint Vincent and the Grenadines	10.7 [9.51-11.91]	13.36 [11.15-15.79]	24.86	27.65 [24.59-30.82]	20.61 [17.18-24.38]	-1.22 [-1.45 to -0.98]
Samoa	6.61 [4.53-8.92]	9.66 [6.02-13.56]	46.14	14.06 [9.84-18.93]	12.36 [7.82-17.15]	-0.44 [-0.55 to -0.33]
Sao Tome and Principe	9.45 [6.77-11.6]	16.3 [11.32-22.25]	72.49	27.39 [19.85-33.37]	26.48 [18.15-36.14]	-0.39 [-0.63 to -0.16]
Saudi Arabia	75.4 [52.3-123.89]	203.77 [148.58-279.31]	170.25	2.85 [2.04-4.59]	2.35 [1.73-3.11]	-0.56 [-0.78 to -0.35]
Senegal	394.69 [290.59-521.09]	849.81 [613.95-1117.4]	115.31	21.85 [16.2-28.64]	20.09 [14.71-26.1]	-0.03 [-0.24 to 0.18]
Serbia	686.28 [561.02-808.6]	630.07 [463.63-829.16]	−8.19	11.78 [9.64-13.74]	8.53 [6.31-11.15]	-1.46 [-1.7 to -1.21]
Seychelles	6.73 [5.66-8.2]	9.25 [7.37-12.01]	37.44	22.06 [18.59-26.91]	15.99 [12.87-20.68]	-0.99 [-1.06 to -0.93]
Sierra Leone	198.02 [141.26-272.23]	460.23 [302.77-647.01]	132.42	19.63 [14.26-26.58]	22.45 [14.92-31.28]	0.84 [0.64 to 1.04]

Nation	Death Cases No.(95% UI)		change absolute number(%)	ASDR per 100,000 No.(95% UI)		1990-2019 EAPC No. (95% CI)
	1990	2019		1990	2019	
Singapore	97.53 [86.28-105.6]	105.42 [91.57-122.88]	8.09	7.57 [6.73-8.18]	2.62 [2.27-3.06]	-4.04 [-4.27 to -3.8]
Slovakia	224.96 [194.17-263.27]	240.2 [158.56-313.2]	6.77	6.99 [6.01-8.18]	5.19 [3.39-6.74]	-0.79 [-0.96 to -0.62]
Slovenia	83.38 [61.52-108.53]	62 [45.16-82.58]	−25.64	6.04 [4.46-7.92]	2.89 [2.13-3.92]	-2.78 [-2.94 to -2.61]
Solomon Islands	25.02 [11.49-43.12]	58.97 [26.55-91.02]	135.69	32.01 [16.3-56.36]	29.44 [15.37-45.45]	-0.18 [-0.24 to -0.11]
Somalia	606.87 [349.45-903.1]	1417.5 [832.05-2231.52]	133.58	33.94 [19.32-49.58]	30.99 [18.2-48.26]	-0.13 [-0.2 to -0.07]
South Africa	2287.55 [1740.71-2974.36]	4415.07 [3643.74-5217.47]	93.00	17.44 [13.2-23.07]	16.64 [13.75-19.58]	0.1 [-0.25 to 0.45]
South Sudan	271.17 [168.44-385.26]	412.75 [238.63-679.5]	52.21	22.61 [14.06-31.5]	18.22 [11.03-28.83]	-0.73 [-0.82 to -0.63]
Spain	934.33 [830.05-998.59]	1149.33 [805.97-1286.34]	23.01	3.37 [2.92-3.6]	2.39 [1.64-2.66]	-1.01 [-1.19 to -0.82]
Sri Lanka	250.32 [210.74-351.49]	499.37 [335.92-687.84]	99.49	4.28 [3.6-6.12]	3.6 [2.43-4.96]	-0.41 [-0.72 to -0.1]
Sudan	221.45 [99.65-302.25]	317.76 [193.87-465.05]	43.49	4.51 [2.04-6.07]	3.27 [2.13-4.62]	-1.08 [-1.13 to -1.02]
Suriname	29.33 [23.99-33.41]	54.43 [42.72-68.53]	85.58	20.49 [16.75-23.5]	16.81 [13.15-21.28]	-0.88 [-1.11 to -0.64]
Sweden	302.01 [275.84-340.42]	278.46 [242.54-307.73]	−7.80	4.01 [3.7-4.58]	2.72 [2.43-3.02]	-1.35 [-1.44 to -1.25]
Switzerland	244.44 [169.62-264.92]	195.51 [162.45-220.06]	−20.02	4.35 [3.08-4.72]	2.17 [1.87-2.45]	-2.37 [-2.53 to -2.21]
Syrian Arab Republic	63.23 [44.9-84.6]	104.17 [74.99-143.7]	64.75	2.3 [1.67-3.13]	1.78 [1.31-2.43]	-1.1 [-1.28 to -0.92]
Tajikistan	119.32 [79.17-135.99]	135.54 [102.51-211.19]	13.59	7.55 [4.95-8.62]	4.68 [3.6-6.87]	-1.56 [-1.99 to -1.13]
Thailand	3135.62 [2332.45-3682.45]	3656.9 [2668.4-5232.53]	16.62	14.76 [11.1-17.29]	6.73 [4.9-9.6]	-3.27 [-3.55 to -2.99]
Timor-Leste	22.37 [12.94-32.79]	38.07 [24.99-58.62]	70.18	12.47 [7.66-18.71]	9.03 [6.06-14.06]	-1.3 [-1.58 to -1.02]
Togo	190.76 [150.11-252.05]	475.08 [339.63-650.36]	149.05	23.87 [18.91-31.46]	20.1 [14.66-26.99]	-0.49 [-0.56 to -0.43]
Tonga	6.52 [5.03-8.08]	7.53 [5.5-10.15]	15.49	21.91 [17.13-27.05]	17.4 [12.8-23.44]	-0.83 [-0.98 to -0.68]

Nation	Death Cases No.(95% UI)		change absolute number(%)	ASDR per 100,000 No.(95% UI)		1990-2019 EAPC No. (95% CI)
	1990	2019		1990	2019	
Trinidad and Tobago	82.69 [75.6-90.25]	108.63 [81.06-143.68]	31.37	18.47 [16.92-20.18]	11.55 [8.59-15.34]	-2.03 [-2.23 to -1.83]
Tunisia	91.65 [66.6-113.17]	168.78 [116.03-230.79]	84.16	3.55 [2.61-4.37]	2.57 [1.77-3.5]	-1.14 [-1.18 to -1.09]
Turkey	851.6 [541.62-1059.43]	1171.48 [800.47-1479.13]	37.56	4.39 [2.79-5.41]	2.53 [1.72-3.19]	-1.86 [-2.3 to -1.43]
Turkmenistan	93.69 [85.45-102.28]	172.58 [122.42-227.11]	84.20	8.06 [7.36-8.78]	7.18 [5.14-9.37]	0.28 [-0.12 to 0.68]
Uganda	865.51 [603.55-1157.28]	2221.74 [1577.22-2899.86]	156.70	23.15 [16.47-30.47]	24.29 [17.61-30.9]	-0.1 [-0.33 to 0.14]
Ukraine	4393.79 [3040.02-4782.3]	2166.15 [1674.17-2909.06]	−50.70	10.34 [7.19-11.23]	5.23 [4.02-7.13]	-3.09 [-3.33 to -2.85]
United Arab Emirates	16.93 [11.9-23.79]	76.12 [54.08-106.93]	349.62	12.26 [7.64-17.88]	6.8 [4.3-9.12]	-1.75 [-2.63 to -0.86]
United Kingdom	2565.88 [2383.01-2676.42]	1692.22 [1552-2253.23]	−34.05	5.75 [5.26-5.92]	2.89 [2.7-3.74]	-2.29 [-2.57 to -2.02]
United Republic of Tanzania	1690.23 [1144.7-2273.39]	3333.03 [2273.74-4648.68]	97.19	26.72 [18.09-35.33]	22.51 [15.48-30.9]	-0.52 [-0.65 to -0.38]
United States of America	6201.57 [5452.13-6488.87]	7994.66 [6756.53-8477.45]	28.91	3.77 [3.28-3.94]	3.05 [2.61-3.22]	-0.69 [-0.83 to -0.55]
United States Virgin Islands	5.84 [4.56-7.07]	7.27 [5.65-9.14]	24.49	11.88 [9.29-14.31]	7.77 [5.99-9.87]	-1.53 [-1.58 to -1.48]
Uruguay	205.6 [189.84-229.18]	229.6 [198.53-258.57]	11.67	10.56 [9.75-11.59]	8.45 [7.39-9.5]	-0.93 [-1.11 to -0.75]
Uzbekistan	534.9 [487.76-586.39]	1172.94 [931.67-1433.05]	119.28	8.01 [7.29-8.8]	8.45 [6.84-10.28]	0 [-0.25 to 0.26]
Vanuatu	5.9 [3.25-8.96]	15.56 [8.56-23.88]	163.73	17.49 [10.37-27.32]	17.07 [9.7-26.01]	-0.4 [-0.61 to -0.19]
Venezuela (Bolivarian Republic of)	1146.51 [1071.47-1275.99]	2390.41 [1743.68-3161.98]	108.49	19.84 [18.39-22.55]	15.18 [11.11-20.08]	-1.36 [-1.59 to -1.13]
Viet Nam	2477.5 [1845.65-3323.08]	4717.7 [3273.31-6161.43]	90.42	10.59 [7.92-14.24]	8.8 [6.11-11.45]	-0.63 [-0.78 to -0.47]
Yemen	132.05 [62.73-195.74]	303.95 [190.88-447.41]	130.18	4.77 [2.36-7.06]	4 [2.52-5.69]	-0.68 [-0.76 to -0.61]

Nation	Death Cases No.(95% UI)		change absolute number(%)	ASDR per 100,000 No.(95% UI)		1990-2019 EAPC No. (95% CI)
	1990	2019		1990	2019	
Zambia	609.8 [423.53-801.22]	1136.5 [755.55-1599.15]	86.37	34.99 [24.98-45.63]	26.42 [17.91-37.04]	-1.37 [-1.57 to -1.17]
Zimbabwe	653.74 [460.58-834.1]	1399.85 [937.47-1949.16]	114.13	28.55 [20.45-36.44]	31.39 [21.68-43.63]	1.46 [0.93 to 1.99]

Supplementary Table S5. The DALY and age-standardized DALY rate of Cervical cancer in 1990 and 2019, and its temporal trends from 1990 to 2019.

Nation	DALY No.(95% UI)		change absolute number(%)	Age Standardized DALY Rate No.(95% UI)		1990-2019 EAPC No. (95% CI)
	1990	2019		1990	2019	
Afghanistan	11599.28 [3400.13-18085.13]	22877.25 [8021.12-36388.26]	97.23	294.12 [88.46-454.94]	238.64 [93.29-368.12]	-0.87 [-1.08 to -0.65]
Albania	1601.48 [1403.33-2203.36]	1814.33 [1244.65-2513.94]	13.29	127.85 [112.42-178.71]	98.42 [68.41-137.9]	-0.63 [-0.83 to -0.43]
Algeria	17941.68 [11757.79-23396.9]	26554.73 [18218.46-36981.33]	48.01	239.74 [161.58-310.59]	133.16 [92.95-182.64]	-2 [-2.12 to -1.87]
American Samoa	39.94 [31.47-54.23]	75.82 [57.32-98.47]	89.83	281.62 [225.86-383.14]	287.21 [217.27-372.02]	0.15 [-0.05 to 0.35]
Andorra	44.53 [31.36-62.31]	77.58 [54.05-107.27]	74.22	155.75 [109.9-217.62]	122 [85.23-168.61]	-0.91 [-1.11 to -0.72]
Angola	24193.64 [15695.86-35454.34]	53777.67 [33635.75-79355.61]	122.28	862.06 [563.78-1251.84]	618.69 [394.47-913.24]	-1.3 [-1.47 to -1.13]
Antigua and Barbuda	105.29 [91.25-121.72]	174.19 [141.11-214.42]	65.44	381.14 [329.41-442.08]	308.72 [250.89-377.22]	-0.93 [-1.11 to -0.75]
Argentina	62634.33 [58238.11-69151.25]	96024.03 [72794.12-106818.51]	53.31	375.55 [348.5-412.38]	366.91 [275.7-408.93]	-0.19 [-0.34 to -0.04]
Armenia	5192.8 [4712.79-5759.49]	4678.75 [3789.27-5731.95]	−9.90	316.29 [287.45-350.78]	222.33 [179.36-273.31]	-1.21 [-1.47 to -0.95]
Australia	10401.6 [9076.96-11040.8]	11275.63 [9633.17-12565.33]	8.40	107.31 [93.3-114.22]	64.41 [55.7-72.27]	-1.48 [-1.91 to -1.05]
Austria	10868.08 [8911.17-11608.76]	5625.2 [5008.28-7051.65]	−48.24	195.81 [159.94-209.45]	76.51 [67.77-96.65]	-3.37 [-3.62 to -3.11]
Azerbaijan	7810.56 [6678.11-9431.6]	11320.83 [8318.58-15804.89]	44.94	249.19 [213.58-306.02]	188.35 [139.57-263.71]	-1.14 [-1.32 to -0.96]
Bahamas	499.25 [434.46-568.95]	813.08 [619.06-1047.44]	62.86	475.86 [415.44-540.96]	354.28 [270.7-456.68]	-1.21 [-1.34 to -1.07]
Bahrain	144.83 [116.25-185.48]	376.22 [282.82-503.55]	159.77	135.43 [110.11-174.31]	74.42 [56.8-99.68]	-2.25 [-2.45 to -2.04]
Bangladesh	121351.61 [55103.61-159389.17]	132532.19 [74025.18-204662.92]	9.21	433.38 [194.25-567.09]	180.42 [100.78-277.14]	-2.81 [-3.06 to -2.57]
Barbados	735.28 [657.42-805.44]	882.32 [702.18-1093.09]	20.00	526.42 [468.09-577.23]	388.56 [308.51-484.21]	-0.99 [-1.07 to -0.9]
Belarus	18540.76 [17127.67-20325.21]	14720.16 [10925.28-20640.18]	−20.61	263.05 [243.22-289.11]	194.83 [142.45-272.77]	-1.42 [-1.63 to -1.21]
Belgium	9130.32 [7869.37-9823.21]	7053.49 [6092.42-7893.18]	−22.75	129.78 [113.49-139.73]	78.16 [68.32-88.47]	-1.79 [-1.94 to -1.65]
Belize	318.74 [276.07-369.61]	1006.21 [821.3-1194.45]	215.68	623.05 [542.95-718.31]	573.7 [470.62-678.98]	-0.58 [-0.97 to -0.19]

Nation	DALY No.(95% UI)		change absolute number(%)	Age Standardized DALY Rate No.(95% UI)		1990-2019 EAPC No. (95% CI)
	1990	2019		1990	2019	
Benin	8960.59 [6948.04-11807.86]	20774.57 [14208.35-29812.02]	131.84	703.85 [553.31-929.01]	608.36 [428.27-861.22]	-0.46 [-0.52 to -0.41]
Bermuda	79.43 [68.07-92.6]	51.75 [40.79-66.07]	−34.85	223.8 [191.97-260.34]	93.44 [72.57-119.44]	-3.59 [-3.86 to -3.31]
Bhutan	732.86 [338.29-1053.2]	701.66 [408.26-1128]	−4.26	450.61 [208.27-640.18]	222.61 [131.29-352.43]	-2.69 [-2.95 to -2.43]
Bolivia (Plurinational State of)	20782.93 [13242.51-26563.95]	34290.3 [23624.42-47692.44]	64.99	1022 [659.2-1298.44]	682.13 [474.07-941.17]	-1.57 [-1.7 to -1.45]
Bosnia and Herzegovina	4867.34 [4273.74-5812.63]	4666.03 [3185.25-6108.42]	−4.14	197.44 [174.25-236.66]	179.4 [120.09-235.69]	-0.42 [-0.61 to -0.24]
Botswana	2832.19 [1785.31-4368.82]	8003.15 [4568.14-12585.15]	182.58	751.1 [479.07-1147.11]	810.03 [468.54-1263.81]	0.26 [-0.01 to 0.53]
Brazil	254453.98 [240125.89-295845.79]	348416.25 [324215.1-404298.38]	36.93	451.35 [424.4-522.6]	268 [249.59-309.88]	-2 [-2.1 to -1.89]
Brunei Darussalam	399.24 [289.43-504.31]	633.88 [497.75-830.29]	58.77	546.1 [411.3-674.07]	305.13 [245.47-395.91]	-1.98 [-2.23 to -1.73]
Bulgaria	14914.96 [13403.06-16676.29]	15434.23 [10557.6-20190.73]	3.48	264.12 [234.27-293.73]	293.89 [189.85-389.9]	1.23 [0.93 to 1.52]
Burkina Faso	22728.76 [16515.27-30424.87]	46561.08 [33164.22-61993.03]	104.86	803.18 [585.37-1080.76]	698.95 [507.68-922.64]	-0.54 [-0.73 to -0.36]
Burundi	18687.63 [11866.23-26271.47]	25370.49 [15534.22-38681.68]	35.76	1176.27 [756.52-1646.8]	818.37 [504.47-1231.79]	-1.77 [-1.97 to -1.57]
Cabo Verde	714.34 [592.53-897.86]	912.3 [715.74-1348.75]	27.71	559.7 [465.85-722.66]	365.13 [288.33-535.6]	-1.31 [-1.59 to -1.03]
Cambodia	16073.15 [8772.15-22809.45]	23732.03 [16721.15-35726.59]	47.65	486.73 [270.54-694.94]	305.98 [215.02-460.08]	-1.77 [-1.87 to -1.67]
Cameroon	22433.87 [17565.67-29905.31]	53264.01 [32945.23-82127.83]	137.43	759.22 [592.81-999.16]	635.16 [403.6-955.34]	-0.56 [-0.75 to -0.36]
Canada	15914.44 [14886.03-16944.41]	21079.2 [17810.03-23425.34]	32.45	96.74 [90.36-103.67]	77.31 [66.46-86.75]	-0.58 [-0.74 to -0.42]
Central African Republic	9297.96 [5830.35-12730.64]	15712.1 [9007.43-23777.98]	68.98	1114.49 [713.49-1522.71]	955.25 [567.83-1427.5]	-0.61 [-0.76 to -0.46]

Nation	DALY No.(95% UI)		change absolute number(%)	Age Standardized DALY Rate No.(95% UI)		1990-2019 EAPC No. (95% CI)
	1990	2019		1990	2019	
Chad	12875.86 [9789.47-17589.43]	27323.32 [18717.84-37265.47]	112.21	751.69 [571.04-1038.51]	761.71 [523.81-1026.84]	0.18 [0.03 to 0.33]
Chile	32787.03 [28319.05-34822.54]	25200.43 [22382.1-30005.25]	−23.14	552.07 [473.26-586.5]	212.58 [188.53-253.96]	-3.58 [-3.82 to -3.34]
China	855358.76 [654483.99-1432990.11]	1622241.69 [892584.72-2090864.16]	89.66	176.4 [135.68-294.69]	157.5 [86.9-202.91]	0.16 [-0.09 to 0.41]
Colombia	55543.03 [51789.66-61613.94]	73601.97 [55301.78-96605.66]	32.51	495.77 [463.64-547.54]	265.5 [199.02-347.9]	-2.58 [-2.78 to -2.38]
Comoros	1151.46 [447.22-1793.59]	2096.39 [1292.04-3134.3]	82.06	859.87 [360.48-1318.84]	707.02 [443.12-1051.25]	-0.92 [-1.17 to -0.68]
Congo	7680.86 [4957.9-10373.27]	14122.04 [8639.6-20834.03]	83.86	1068.34 [711.52-1438.64]	751.57 [473.04-1086.01]	-1.28 [-1.49 to -1.08]
Costa Rica	4760.91 [4166.63-5138.83]	6150.36 [4551.39-8252.44]	29.18	453.33 [396.84-489.77]	222.04 [164.39-298.15]	-2.97 [-3.4 to -2.55]
Cote d'Ivoire	19060.98 [14025.84-25357.53]	41431.49 [27503.56-59189.41]	117.36	672.74 [511.79-879.95]	569.24 [385.3-795.51]	-0.38 [-0.51 to -0.25]
Croatia	7209.12 [6072.76-8014.87]	3973.13 [2995.79-5158.68]	−44.89	213.49 [180.3-237.43]	108.55 [80.3-142.98]	-2.23 [-2.59 to -1.87]
Cuba	18962.66 [16444.43-20473.21]	20342.66 [15567.34-25454.07]	7.28	356.95 [309.72-385.12]	242.78 [184.66-304.63]	-1.52 [-1.67 to -1.38]
Cyprus	462.29 [371.79-668.85]	690.12 [467.4-820.51]	49.28	110.53 [88.92-158.84]	72.28 [49.31-85.95]	-1.38 [-1.52 to -1.24]
Czechia	18699.71 [17542.82-19809.61]	11400.77 [9077.29-14209.12]	−39.03	274.53 [256.81-291.3]	132.81 [105.37-168.05]	-2.55 [-2.65 to -2.45]
Democratic People's Republic of Korea	33565.22 [22098.54-58251.44]	48247.31 [30550.5-72213.92]	43.74	312.19 [207.22-539.82]	279.56 [175.29-415.39]	-0.2 [-0.32 to -0.09]
Democratic Republic of the Congo	90856.12 [60245.29-122379.43]	172684.72 [110519.87-242280.57]	90.06	811.92 [550.41-1085.86]	683.21 [440.04-957.89]	-0.54 [-0.67 to -0.41]
Denmark	9385.74 [7310.34-9969.24]	4248.49 [3768.37-5867.54]	−54.73	265.23 [206.22-282.92]	94.17 [82.88-128.45]	-3.75 [-4.1 to -3.4]
Djibouti	855.82 [503.54-1299.84]	2784.45 [1529.47-5070.68]	225.35	800.6 [488.78-1178.7]	675.46 [387.57-1185.74]	-0.66 [-0.8 to -0.52]

Nation	DALY No.(95% UI)		change absolute number(%)	Age Standardized DALY Rate No.(95% UI)		1990-2019 EAPC No. (95% CI)
	1990	2019		1990	2019	
Dominica	234.52 [192.07-282.75]	213.36 [161.09-276.26]	−9.02	706.62 [573.73-861.25]	535.72 [401.54-695.38]	-1.11 [-1.19 to -1.04]
Dominican Republic	9124.67 [7629.96-11894.28]	20912.88 [14432.97-29435.25]	129.19	381.81 [320.92-497.22]	405.51 [281.44-573.79]	0.33 [0.17 to 0.48]
Ecuador	17328.04 [14821.2-20225.04]	31893.57 [23923.44-43545.38]	84.06	535.57 [453.8-621.31]	383.79 [286.38-523.03]	-0.97 [-1.23 to -0.72]
Egypt	9528.9 [8038.21-12185.86]	15480.08 [10385.04-22751.62]	62.45	54.39 [46.62-70.55]	45.13 [30.67-65.66]	-0.35 [-0.46 to -0.24]
El Salvador	10021.77 [8935.54-12925.64]	15591.8 [11046.05-21226.64]	55.58	559.15 [499.1-721.63]	463.1 [328.34-631.33]	-1.3 [-1.72 to -0.89]
Equatorial Guinea	1241.73 [727.18-1807.29]	1915.36 [1097.43-3282.21]	54.25	899.27 [526.59-1309.48]	508.3 [302.02-846.19]	-2.12 [-2.29 to -1.94]
Eritrea	8633.82 [5270.23-13362.96]	20127.42 [12747.62-29694.16]	133.12	1047.27 [639.22-1606.41]	973.58 [614.4-1424.77]	-0.15 [-0.22 to -0.08]
Estonia	3387.79 [2943.53-3665.96]	1734.55 [1294.29-2256.56]	−48.80	307.74 [265.34-334.5]	160.82 [119.98-213.43]	-2.68 [-2.88 to -2.48]
Eswatini	1479.56 [957.8-2105.22]	3213.08 [1617.12-5577.24]	117.16	710.49 [459.87-1006.76]	794.74 [400.57-1360.64]	1.01 [0.28 to 1.73]
Ethiopia	124487.99 [58285.33-192605.88]	133579.84 [90860.96-219607.15]	7.30	914.79 [470.11-1411.45]	497.94 [343.9-810.21]	-2.56 [-2.78 to -2.35]
Fiji	2152.69 [1068.78-2858.58]	3008.66 [1243.74-4169.56]	39.76	839.53 [413.1-1108.9]	680.13 [283.37-939.18]	-0.28 [-0.61 to 0.05]
Finland	2480.27 [2287.27-2751.86]	2364.24 [1776.05-2689.96]	−4.68	67.2 [61.76-75.82]	47.49 [38.57-54.33]	-0.92 [-1.08 to -0.75]
France	52750.79 [44739.55-56248.43]	42685.95 [36718.6-48128.52]	−19.08	139.2 [118.87-148.44]	82.27 [71.37-93.13]	-1.71 [-1.86 to -1.56]
Gabon	2460.38 [1697.01-3336.78]	3418.28 [2150.82-5047.82]	38.93	755.24 [518.24-1021.93]	500.16 [318.3-731.05]	-1.53 [-1.83 to -1.23]
Gambia	1051.75 [698.94-1474.08]	3305.49 [2206.95-4701.28]	214.28	465.77 [310.54-647.99]	523.79 [356.56-732.28]	0.19 [-0.05 to 0.44]
Germany	90938.33 [84165.93-95882.33]	61055.8 [54569.25-67712.53]	−32.86	157.76 [139.76-167.13]	86.56 [77.18-97.32]	-2.16 [-2.28 to -2.04]
Ghana	32380.47 [23849.37-44095.91]	60248.1 [39856.2-84249.74]	86.06	733.5 [550.81-1000.45]	516.85 [344.09-709.92]	-1.29 [-1.39 to -1.19]

Nation	DALY No.(95% UI)		change absolute number(%)	Age Standardized DALY Rate No.(95% UI)		1990-2019 EAPC No. (95% CI)
	1990	2019		1990	2019	
Greece	9484.69 [8570.2-10204.63]	7723.52 [6966.17-8695.35]	−18.57	134.6 [120.52-144.84]	86.06 [77.46-96.73]	-1.53 [-1.79 to -1.26]
Greenland	124.03 [99.4-154.99]	96.77 [72.88-127.79]	−21.98	567.44 [455.23-700.88]	298.21 [224.13-393.34]	-2.65 [-2.83 to -2.48]
Grenada	256.43 [221.79-295.01]	301.68 [247.68-352.98]	17.65	746.63 [643.95-864.73]	525.91 [427.55-613.68]	-1.09 [-1.39 to -0.8]
Guam	101.58 [83.13-126.61]	171.64 [131.11-217.63]	68.97	222.88 [182.37-275.55]	187.8 [142.79-236.93]	-0.82 [-1.21 to -0.42]
Guatemala	11611.94 [9350.46-20926.62]	39390.88 [28114.65-51910.79]	239.23	484.16 [394.89-855.1]	559.06 [395-734.61]	0.46 [-0.01 to 0.94]
Guinea	26494.97 [20258.68-33276.28]	41279.99 [29273.08-56036.5]	55.80	1351.39 [1033.4-1701.11]	1143.8 [813.77-1527.84]	-0.43 [-0.49 to -0.38]
Guinea-Bissau	3123.67 [2030.55-4508.99]	5293.51 [3270.09-7581.37]	69.46	1085.85 [707.95-1560.41]	937.63 [586.74-1325.59]	-0.31 [-0.42 to -0.2]
Guyana	2297.77 [1852.23-2797.56]	2572.74 [1884.27-3473.12]	11.97	910.53 [741.94-1104.08]	683.14 [502.45-914.26]	-1.04 [-1.23 to -0.85]
Haiti	29639.11 [11410.63-40969.87]	46380.83 [21162.31-69676.09]	56.49	1358.26 [558.52-1843.94]	913.06 [419.09-1364.03]	-1.23 [-1.33 to -1.14]
Honduras	5977.26 [4545.45-7644.03]	13189.64 [7966.25-20744.85]	120.66	420.25 [322.77-541.17]	343.73 [210.79-530.99]	-0.8 [-0.94 to -0.66]
Hungary	20610.16 [19280.09-22437.12]	13341.9 [10591.5-16841]	−35.27	291.99 [271.32-317.76]	170.06 [132.84-217.74]	-2.12 [-2.28 to -1.96]
Iceland	161.15 [140.14-179.63]	127.99 [106.49-150.4]	−20.58	120.09 [103.81-134.65]	54.93 [45.72-64.42]	-2.94 [-3.16 to -2.72]
India	1029220.62 [808714.75-1290947.99]	1554489.11 [1198222.57-2094137.4]	51.04	355.76 [281.53-447.7]	239.49 [184.74-325.15]	-1.6 [-1.82 to -1.37]
Indonesia	209574.82 [125659.31-310811.1]	291925.32 [190114.25-465941.43]	39.29	316.01 [191.9-475.38]	216.87 [143.05-352.41]	-1.22 [-1.36 to -1.07]
Iran (Islamic Republic of)	13987.01 [9830.42-16221.23]	22367.43 [16198.67-25705.12]	59.92	90.42 [63.85-107.54]	54.11 [38.94-62.2]	-1.9 [-2.09 to -1.7]
Iraq	3944.48 [2856.87-5500.88]	10307.01 [7148.18-14210.95]	161.30	85.55 [61.93-118]	70.2 [49.79-95.2]	-0.77 [-0.91 to -0.63]
Ireland	2616.52 [2329.76-2874.06]	2979.87 [2167.18-3495.39]	13.89	138.91 [121.54-152.81]	90.3 [63.79-105.93]	-1.2 [-1.36 to -1.05]

Nation	DALY No.(95% UI)		change absolute number(%)	Age Standardized DALY Rate No.(95% UI)		1990-2019 EAPC No. (95% CI)
	1990	2019		1990	2019	
Israel	2167.09 [1977.16-2565.44]	3872.16 [3007.55-4379.29]	78.68	87.98 [80.08-104.66]	72.51 [56.03-82.16]	-0.83 [-0.94 to -0.72]
Italy	23716.91 [22557.15-28455.97]	35796.71 [22068.2-39678.4]	50.93	57.43 [54.58-69.29]	64.96 [40.66-71.73]	1 [0.74 to 1.26]
Jamaica	4826.32 [4188.54-5261.69]	7777.35 [5783.52-10183.02]	61.14	545.65 [469.67-593.95]	511.65 [379.85-672.19]	-0.26 [-0.6 to 0.08]
Japan	92262.22 [87262.21-103694.55]	97453.56 [76367.69-106871.01]	5.63	104.7 [99.43-117.74]	90.99 [67.46-99.86]	-0.13 [-0.25 to -0.01]
Jordan	909.42 [689.39-1166.36]	2148.34 [1514.27-2875.05]	136.23	106.21 [81.44-135.46]	55.25 [39.79-73.64]	-2.6 [-2.85 to -2.34]
Kazakhstan	27977.66 [25583.35-30993.83]	27646.41 [22960.38-33599.9]	−1.18	355.99 [325.64-393.77]	262.01 [217.61-317.76]	-0.66 [-1.03 to -0.28]
Kenya	20691.33 [13684.1-34169.42]	60420.87 [40670.1-98744.05]	192.01	390.27 [258.53-636.44]	395.3 [266.45-641.69]	0.29 [0.09 to 0.48]
Kuwait	333.98 [273.01-395.47]	708.5 [531.39-1034.83]	112.14	92.15 [73.96-109.07]	44.34 [33.14-62.29]	-2.11 [-2.42 to -1.79]
Kyrgyzstan	7271.8 [6306.02-7946.6]	8758.07 [6991.89-10460.01]	20.44	411.64 [360.62-449.93]	291.78 [233.19-347.31]	-1.14 [-1.37 to -0.9]
Lao People's Democratic Republic	7698.88 [3872.21-11522.07]	8380.81 [5152.1-12453.61]	8.86	572.86 [300.19-865.17]	289.76 [180.22-437.83]	-2.59 [-2.69 to -2.5]
Latvia	4954.08 [4280.8-5377.65]	2302.7 [1696.24-3159.49]	−53.52	255.6 [225.76-278.21]	134.7 [96.35-188.74]	-2.38 [-2.67 to -2.09]
Lebanon	1560.01 [1100.71-2014.49]	2069.33 [1397.57-2871.62]	32.65	120.71 [87.07-154.25]	71.83 [48.58-99.59]	-1.92 [-2.03 to -1.8]
Lesotho	3522.27 [2279.23-5280.08]	9028.39 [4434.24-15654.94]	156.32	600.41 [390.88-894.47]	1087.77 [542.05-1883.99]	3.44 [2.84 to 4.05]
Liberia	4565.36 [3425.48-6121.53]	8819.49 [5767.63-12532.47]	93.18	765.1 [578.56-1022.04]	620.59 [413.31-875.05]	-0.79 [-0.91 to -0.67]
Libya	1607.13 [1080.98-2096.5]	4055.46 [2525.74-5527.28]	152.34	155.43 [105.53-201.39]	124.02 [82.41-167.15]	-0.67 [-0.95 to -0.39]
Lithuania	7127.37 [6018.69-7700.34]	4090.98 [3184.92-5097.18]	−42.60	293.7 [249.87-317.45]	166.86 [126.83-212.66]	-1.92 [-2.16 to -1.67]
Luxembourg	339.73 [296.94-372.81]	248.59 [208.55-310.92]	−26.83	129.49 [112.17-142.3]	55.34 [46.35-69.27]	-2.89 [-3.04 to -2.73]
Madagascar	31204.65 [21791.28-41736.69]	59781.8 [38570.96-87712.13]	91.58	910.14 [627.39-1208.71]	718.9 [462.52-1047.43]	-0.88 [-0.94 to -0.82]

Nation	DALY No.(95% UI)		change absolute number(%)	Age Standardized DALY Rate No.(95% UI)		1990-2019 EAPC No. (95% CI)
	1990	2019		1990	2019	
Malawi	26461.27 [18815.33-35536.25]	42865.34 [26944.45-64501.38]	61.99	988.47 [712.56-1319.25]	820.33 [531.31-1198.96]	-0.77 [-1.02 to -0.53]
Malaysia	21351.66 [14469.5-24686.25]	35386.16 [25188.9-47107.94]	65.73	376.97 [251.89-432.9]	243.91 [174.02-323.1]	-1.89 [-2.2 to -1.58]
Maldives	219.68 [87.55-315.28]	185.4 [142.23-245.92]	−15.60	410.91 [169.95-589.38]	110.48 [85.88-144.15]	-5.06 [-5.42 to -4.71]
Mali	21089.59 [15771.44-26422.34]	31945.22 [21944.23-44888.25]	51.47	804.34 [604.91-1000.67]	580.52 [400.89-803.01]	-1.36 [-1.47 to -1.25]
Malta	225.33 [196.93-251.74]	186.26 [154.52-225.86]	−17.34	99.08 [86.33-110.42]	50.94 [42.29-61.79]	-1.87 [-2.12 to -1.61]
Marshall Islands	78.17 [52.2-117.04]	164.83 [92.06-260.24]	110.86	725.03 [496.46-1113.53]	696.44 [400.6-1093.95]	-0.2 [-0.37 to -0.02]
Mauritania	5225.68 [3681.7-6947.55]	6565.37 [4430.77-9352.65]	25.64	872.15 [617-1155.34]	523.83 [357.58-733.13]	-1.53 [-1.63 to -1.43]
Mauritius	1258.29 [1121.89-1391.69]	1482.37 [1157.55-1915.77]	17.81	286.06 [255.78-316.62]	162.86 [126.99-211.25]	-2.49 [-2.71 to -2.27]
Mexico	181315.19 [156182.18-190077.05]	188921.06 [150714.84-254978.3]	4.19	676.78 [568.44-710.47]	282.99 [226.19-380.52]	-3.39 [-3.61 to -3.17]
Micronesia (Federated States of)	223.49 [139.92-348.69]	274.52 [156.44-443.92]	22.83	787.61 [508.17-1228.66]	622.06 [363.15-1000.78]	-0.76 [-0.9 to -0.62]
Mongolia	3624.3 [2802.5-4897.52]	6071.98 [4294.73-8721.56]	67.54	583.47 [452.93-774.4]	363.49 [262.7-509.99]	-2.19 [-2.46 to -1.92]
Montenegro	588.4 [483.95-762.18]	702.94 [556.66-879.38]	19.47	174.02 [142.76-225.2]	161.37 [127.8-201.84]	-0.39 [-0.83 to 0.06]
Morocco	22966.62 [14925.21-28486.68]	43102.96 [26250.68-60139.02]	87.68	281.05 [185.51-345.58]	236.51 [150.1-326.2]	-0.55 [-0.66 to -0.44]
Mozambique	38182.81 [24288.52-55110.23]	74055.42 [44421.72-113127.03]	93.95	918.05 [596.76-1303.75]	915.04 [561.97-1372.26]	0.22 [-0.07 to 0.5]
Myanmar	77755.85 [43578.52-120935.7]	72415.12 [48922.65-116911.29]	−6.87	521.01 [297.92-814.26]	247.28 [167.17-404.93]	-2.88 [-3.13 to -2.63]
Namibia	1871.06 [1235.26-2762.36]	4485.69 [2857.73-6736.25]	139.74	437.88 [292.34-650.12]	486.53 [316.49-722.51]	0.58 [0.4 to 0.76]
Nepal	31253.88 [14450.43-43339.04]	35180.59 [21805.38-50994.39]	12.56	508.47 [236.17-695.19]	259.06 [161.28-373.19]	-2.38 [-2.84 to -1.92]

Nation	DALY No.(95% UI)		change absolute number(%)	Age Standardized DALY Rate No.(95% UI)		1990-2019 EAPC No. (95% CI)
	1990	2019		1990	2019	
Netherlands	9225.13 [8422.99-9837.78]	8757.42 [7714.56-9798.6]	−5.07	96.07 [87.96-102.81]	65.34 [57.67-73.69]	-1.4 [-1.53 to -1.27]
New Zealand	3533.24 [2304.61-3845.22]	2301.82 [2045.43-2597.8]	−34.85	188.04 [121.21-205.4]	71.08 [62.75-80.39]	-3.16 [-3.62 to -2.69]
Nicaragua	7641.37 [6064.81-8662.49]	12907.58 [9949.35-17517.59]	68.92	719.61 [569.81-808.62]	465.42 [359.89-626.32]	-1.68 [-1.84 to -1.51]
Niger	15323.31 [11054.87-21243.12]	37636.15 [25148.48-53051.86]	145.61	803.87 [585.5-1111.26]	711.53 [487.57-984.2]	-0.62 [-0.72 to -0.51]
Nigeria	103434.24 [68836.59-155090.83]	225446.08 [147706.78-332051.72]	117.96	436.68 [292.44-646.45]	359.84 [239.42-520.17]	-0.57 [-0.64 to -0.51]
North Macedonia	2373.64 [2051.71-3179.75]	2886.02 [2076.12-3854.42]	21.59	227.25 [196.69-305.26]	190.84 [137.26-255.65]	-1.2 [-1.68 to -0.73]
Northern Mariana Islands	87.69 [62.15-118.87]	129.81 [95.55-169.98]	48.03	657.31 [486.25-871.08]	461.63 [341.68-603.48]	-1.07 [-1.28 to -0.87]
Norway	4310.67 [4039.25-4538.7]	3129.89 [2837.45-3490.31]	−27.39	155.93 [145.18-164.12]	80.43 [73.11-90.05]	-2.34 [-2.44 to -2.24]
Oman	485.87 [330.41-668.75]	763.71 [561.3-965.15]	57.18	131.57 [89.5-180.26]	78.01 [58.73-96.62]	-1.45 [-1.77 to -1.12]
Pakistan	48395.62 [39052.75-60790.3]	110786.03 [78203.39-158090.37]	128.92	151.8 [122.5-191.67]	149.93 [107.17-210.77]	-0.35 [-0.64 to -0.05]
Palestine	534.26 [344.75-730.62]	978.27 [651.92-1209.7]	83.11	101.84 [65.98-137.04]	69.83 [44.73-86.02]	-1.28 [-1.58 to -0.99]
Panama	5742.64 [4334.7-6276.86]	6791.11 [4956.71-8995.95]	18.26	657.79 [502.43-716.55]	321.04 [234.3-425.46]	-2.63 [-2.92 to -2.35]
Papua New Guinea	5531.1 [3192.36-8821.74]	15199.73 [8521.45-22734.64]	174.80	454.62 [269.17-752.37]	451.63 [262.37-687.26]	0.17 [0.07 to 0.26]
Paraguay	8341.75 [6401.24-9786.34]	16858.78 [11824.98-22804.72]	102.10	620.61 [471.98-728.89]	535.09 [376.32-724.12]	-0.79 [-1.05 to -0.52]
Peru	41139.04 [33949.75-50928.22]	63410.11 [43410.65-87805.83]	54.14	566.72 [470.34-700.28]	366.03 [250.6-507.31]	-1.78 [-2.01 to -1.54]
Philippines	59775.52 [46790.54-71062.82]	110566.1 [78846.53-147798.87]	84.97	292.84 [238.34-356.17]	226.75 [164.63-303.9]	-0.82 [-1.01 to -0.64]
Poland	84755.18 [77856.18-87786.08]	55300.13 [43000.52-70365.51]	−34.75	366.94 [339.3-380.21]	178.24 [138.08-229.12]	-2.76 [-2.94 to -2.58]
Portugal	12786.99 [11513.08-13746.8]	9049.83 [7933.19-10324.1]	−29.23	195.42 [173.16-210.85]	96.32 [83.79-110.44]	-2.69 [-2.86 to -2.51]
Puerto Rico	3117.61 [2846.31-3431.78]	3310.12 [2471.83-4357.71]	6.17	162.58 [148.18-178.84]	124.59 [91.04-165.92]	-0.93 [-1.12 to -0.74]

Nation	DALY No.(95% UI)		change absolute number(%)	Age Standardized DALY Rate No.(95% UI)		1990-2019 EAPC No. (95% CI)
	1990	2019		1990	2019	
Qatar	99.09 [72.44-140.01]	361.58 [258.3-514.1]	264.90	158.1 [118.14-218.37]	104.18 [78.22-139.27]	-1.21 [-1.47 to -0.96]
Republic of Korea	41124.16 [34271.83-48091.63]	32673.01 [26790.44-41287.02]	−20.55	201.47 [172.86-243.22]	77.9 [63.89-97.95]	-3.91 [-4.19 to -3.63]
Republic of Moldova	9323.7 [7919.56-10072.36]	6049.45 [4875.68-7436.35]	−35.12	364.28 [308.33-392.38]	218.96 [174.7-270.94]	-1.16 [-1.45 to -0.86]
Romania	63775.17 [59773.72-68058.07]	54531.64 [37620.99-69295.09]	−14.49	462.62 [432.93-492.65]	364.48 [249.97-466.88]	-1.09 [-1.37 to -0.81]
Russian Federation	199873.44 [187705.45-220554.12]	217304.98 [162262.97-270370.08]	8.72	192.57 [180.52-213.93]	199.87 [145.46-250.63]	0.03 [-0.26 to 0.33]
Rwanda	26040.01 [17420.51-35953.59]	28344.2 [18353.77-43501.61]	8.85	1274.62 [872.59-1746.89]	643.91 [423.28-974.37]	-3.25 [-3.63 to -2.87]
Saint Lucia	369.12 [327.37-410.46]	473.05 [380.29-584.3]	28.16	750.89 [666.1-835.56]	426.97 [343.36-525.68]	-2.24 [-2.51 to -1.97]
Saint Vincent and the Grenadines	338.89 [299-382.11]	420.41 [345.9-504.71]	24.06	879.84 [771.26-996.01]	653.61 [538.29-781.93]	-1.36 [-1.57 to -1.15]
Samoa	228.71 [149.72-311.24]	335.23 [197.17-487.65]	46.57	460.95 [307.02-624.18]	409.14 [244.4-590.47]	-0.38 [-0.48 to -0.28]
Sao Tome and Principe	304.1 [206.92-383.19]	566.26 [395.09-788.45]	86.21	847.69 [593.07-1063.39]	806.2 [560.01-1102.19]	-0.53 [-0.83 to -0.22]
Saudi Arabia	2486.19 [1680.77-4337.44]	7724.97 [5493.67-10955.82]	210.72	77.97 [53.93-131.98]	64.92 [48.08-88.08]	-0.46 [-0.67 to -0.24]
Senegal	13947.78 [9951.58-18628.84]	28213.84 [19676.28-37989]	102.28	685.36 [495.91-912.18]	594.14 [423.11-792.84]	-0.29 [-0.5 to -0.08]
Serbia	22154.36 [17936.01-25722.16]	17656.26 [12820.35-23097.61]	−20.30	383.66 [310.16-441.17]	272.05 [196.42-359.46]	-1.58 [-1.84 to -1.32]
Seychelles	202.57 [169.7-242.99]	276.39 [217.66-361.78]	36.44	691.78 [576.6-828.32]	468.49 [371.07-612.83]	-1.17 [-1.26 to -1.08]
Sierra Leone	6707.94 [4645.35-9249.76]	16326.57 [10500.15-23200.82]	143.39	604.71 [422.63-839.9]	691.28 [450.32-974.43]	0.83 [0.62 to 1.04]
Singapore	3197.99 [2826.12-3494.57]	2878.8 [2488.9-3422.59]	−9.98	225.26 [198.11-244.91]	71.95 [62.24-85.34]	-4.31 [-4.56 to -4.06]
Slovakia	7174.45 [6194.25-8276.64]	7048.37 [4564.24-9258.75]	−1.76	233.19 [201.06-269.02]	170.3 [110.45-225.67]	-0.86 [-1.03 to -0.69]
Slovenia	2412.67 [1773.8-3249.99]	1499.77 [1100.56-2054.46]	−37.84	188.17 [137.56-256.08]	87.45 [62.74-121.35]	-2.97 [-3.17 to -2.77]

Nation	DALY No.(95% UI)		change absolute number(%)	Age Standardized DALY Rate No.(95% UI)		1990-2019 EAPC No. (95% CI)
	1990	2019		1990	2019	
Solomon Islands	998.07 [413.34-1683.7]	2383.36 [951.37-3733.7]	138.80	1114.26 [502.39-1911.87]	1018.69 [457.59-1570.95]	-0.16 [-0.23 to -0.08]
Somalia	23619.57 [13584.28-35460.42]	52768.58 [30740.8-82523.31]	123.41	1151.3 [663.13-1709.34]	1013.76 [591.13-1575.14]	-0.28 [-0.34 to -0.22]
South Africa	84711.28 [64877.44-106751.39]	141034.74 [114827.09-167930.73]	66.49	597.49 [458.25-764.65]	500.44 [407.83-596.02]	-0.29 [-0.63 to 0.04]
South Sudan	9758.47 [5914.7-14335.76]	15097.28 [8356.97-25850.76]	54.71	725.75 [446.17-1041.08]	566.64 [328.63-932]	-0.84 [-0.97 to -0.72]
Spain	26015.77 [22087.85-27803.8]	27837.92 [18901.45-31080.72]	7.00	104.73 [87.61-111.95]	71.2 [49.51-79.92]	-1.24 [-1.39 to -1.09]
Sri Lanka	8561.08 [7143.11-11573.95]	14069.87 [9437.73-19608.16]	64.35	128.96 [108.39-178]	101 [67.8-140.67]	-0.71 [-0.99 to -0.43]
Sudan	7620.59 [3432.55-10637.12]	10964.51 [6320.4-16806.81]	43.88	137.69 [61.93-189.52]	93.66 [56.31-137.99]	-1.3 [-1.35 to -1.25]
Suriname	984.6 [783.62-1137.19]	1761.31 [1363.03-2234.33]	78.89	651.05 [527.37-750.78]	543.46 [419.2-691.2]	-0.91 [-1.15 to -0.67]
Sweden	7314.25 [6794.08-8419.93]	5993.93 [5389.32-6650.56]	−18.05	118.69 [109.99-136.88]	75.64 [68.77-84.65]	-1.48 [-1.57 to -1.39]
Switzerland	6063.57 [4439.68-6594.32]	4262.42 [3689-4839.71]	−29.70	124.18 [93.66-135.3]	58.09 [50.73-66.63]	-2.68 [-2.84 to -2.51]
Syrian Arab Republic	2218.35 [1539.71-2962.19]	3189.72 [2236.64-4498.55]	43.79	68.49 [48.28-90.74]	46.56 [33.24-64.92]	-1.54 [-1.78 to -1.29]
Tajikistan	3985.68 [2831.57-4577.81]	4731.54 [3509.27-7633.77]	18.71	243.88 [170.22-281.14]	136.39 [103.44-212.47]	-2.06 [-2.51 to -1.6]
Thailand	110301.48 [80340.07-130587.21]	107651.68 [75549.71-159386.61]	−2.40	468.74 [345.47-551.71]	205.29 [143.64-302.08]	-3.47 [-3.8 to -3.14]
Timor-Leste	845.37 [470.24-1228.3]	1237.87 [729.51-1887.4]	46.43	397.55 [232.69-580.22]	278.94 [170.96-426.31]	-1.45 [-1.78 to -1.12]
Togo	7097.13 [5479.58-9496.76]	16681.32 [11587.45-23482.65]	135.04	755.55 [593.5-1003.88]	609.53 [430.7-842.25]	-0.67 [-0.74 to -0.6]
Tonga	212.15 [161.46-269.51]	219.66 [157.38-304.84]	3.54	657.29 [503.84-827.26]	504.27 [363.43-697.2]	-1 [-1.16 to -0.85]
Trinidad and Tobago	2519.01 [2300.58-2873.86]	3092.88 [2225.51-4188.73]	22.78	534.78 [488.6-592.69]	341.27 [244.6-463.63]	-1.97 [-2.17 to -1.76]

Nation	DALY No.(95% UI)		change absolute number(%)	Age Standardized DALY Rate No.(95% UI)		1990-2019 EAPC No. (95% CI)
	1990	2019		1990	2019	
Tunisia	3063.97 [2168.48-3811.58]	5116.66 [3403.87-7110.67]	66.99	107.08 [77.41-132.47]	75.12 [50.45-104.04]	-1.27 [-1.31 to -1.23]
Turkey	26831.1 [16780.81-33964.13]	29205.21 [20278.93-37011.79]	8.85	126.7 [79.93-158.73]	61.84 [42.93-78.27]	-2.58 [-2.94 to -2.23]
Turkmenistan	3264.7 [2959.67-3591.3]	6282.28 [4416.46-8339.47]	92.43	266.86 [241.84-291.44]	250.4 [176.46-332.23]	0.52 [0.11 to 0.93]
Uganda	30725.63 [20890.53-41970.5]	79671.81 [56135.75-107037.74]	159.30	731.46 [502.42-987.77]	756.66 [534.93-996.21]	-0.26 [-0.54 to 0.02]
Ukraine	125376.07 [85347.54-136242.35]	62407.18 [47423.32-85637.71]	−50.22	322.59 [221.04-350.79]	171.49 [128.53-238.82]	-2.91 [-3.17 to -2.65]
United Arab Emirates	563.36 [398.56-780.99]	2869.56 [1998.1-4185.36]	409.37	298.16 [199.5-420.54]	168.62 [117.32-230.98]	-1.77 [-2.42 to -1.12]
United Kingdom	70679.64 [61766.14-72887.06]	43304.06 [40596.4-55322.84]	−38.73	185.8 [153.79-191.84]	90.26 [85.09-110.18]	-2.43 [-2.72 to -2.15]
United Republic of Tanzania	60181.51 [40810.02-82597.11]	117926.43 [77928.3-169591.85]	95.95	856.57 [584.67-1164.08]	702.42 [469.38-980.09]	-0.62 [-0.77 to -0.46]
United States of America	190831.13 [161824.28-199732.44]	224783.24 [193183.87-237126.25]	17.79	126.54 [106.2-132.28]	98.77 [86.19-104.29]	-0.86 [-1.02 to -0.7]
United States Virgin Islands	192.4 [148.93-236.75]	188.85 [144.24-241.85]	−1.85	358.55 [278.49-440.14]	223.88 [167.92-293.95]	-1.71 [-1.78 to -1.64]
Uruguay	6334.84 [5800.07-6859.64]	6259.61 [5501.47-7045.83]	−1.19	355.49 [324.79-386.44]	275.05 [240.42-311.8]	-1.06 [-1.2 to -0.91]
Uzbekistan	18402.7 [16721.83-20319.32]	43308.08 [34038.28-53196.41]	135.34	266.46 [242.32-292.56]	276.85 [219.9-339.05]	-0.14 [-0.42 to 0.14]
Vanuatu	215.85 [115.76-335.5]	562.31 [298.09-880.18]	160.51	539.24 [300.64-827.06]	544.48 [298.12-842.03]	-0.34 [-0.56 to -0.12]
Venezuela (Bolivarian Republic of)	41044.92 [38094.42-44495.61]	80373.48 [57627.56-109619.05]	95.82	637.03 [591.9-695.22]	505.7 [363.38-688.29]	-1.19 [-1.44 to -0.94]
Viet Nam	77908.98 [56911.21-102264.33]	139882.5 [96361.09-184065.43]	79.55	323.87 [237.7-426.44]	248.94 [173.03-325.09]	-0.91 [-1.04 to -0.78]
Yemen	4483.85 [2018.73-6879.3]	10484.31 [6242.36-15903.99]	133.82	143.74 [68.2-214.03]	117.57 [72.58-172.6]	-0.79 [-0.88 to -0.71]
Zambia	23580.07 [16190.32-31335.79]	43115.8 [28042.89-61237.25]	82.85	1181.58 [819.63-1553.41]	861.7 [568.8-1229.52]	-1.52 [-1.74 to -1.3]

Nation	DALY No.(95% UI)		change absolute number(%)	Age Standardized DALY Rate No.(95% UI)		1990-2019 EAPC No. (95% CI)
	1990	2019		1990	2019	
Zimbabwe	21931.08 [15377.99-28266.35]	48176.18 [31622.14-68387.32]	119.67	859.67 [599.63-1106.71]	957.22 [639-1349.6]	1.67 [1.06 to 2.28]

Supplementary Table S6. Age distribution of incidence (per 100,000) for Cervical cancer in different countries in 2019.

country	<5	5 to 9	10 to 14	15 to 19	20 to 24	25 to 29	30 to 34	35 to 39	40 to 44	45 to 49	50 to 54	55 to 59	60 to 64	65 to 69	70 to 74	75 to 79	80 to 84	85 to 89	90 to 94	95+
Afghanistan	0	0	0	0.3934706	1.2543836	3.5936422	8.638769	15.666743	21.098223	23.600003	26.242967	34.433001	29.574049	31.70772	33.16489	31.90241	32.78396	26.67046	20.63698	18.07804
Albania	0	0	0	0.4821539	2.7111797	5.3127798	10.943451	14.321325	16.166238	16.848599	17.026869	15.060088	16.023446	15.73684	17.79889	19.09160	20.13603	15.34843	13.03509	14.95474
Algeria	0	0	0	0.2556894	1.0884975	3.6527496	9.791328	16.316043	21.005722	20.417661	19.577589	22.079090	20.632197	20.94553	20.47054	22.89117	26.59216	46.39480	42.94375	23.70642
American Samoa	0	0	0	0.4699349	1.4388117	5.2308567	15.444873	28.061578	44.288164	39.229250	43.131968	49.052743	36.616603	47.02358	52.49536	45.18671	43.76159	78.77832	80.75579	86.11392
Andorra	0	0	0	0.3234562	1.6505083	6.9310623	17.292991	24.714806	28.086521	26.023700	25.775762	26.398851	24.282414	25.61628	26.18625	27.31447	28.28518	27.42381	28.39611	33.90602
Angola	0	0	0	0.7811534	2.9900203	9.9575795	25.931076	38.559459	53.638894	61.460549	75.500565	86.443774	83.676056	85.58676	85.45418	81.90981	83.74927	81.50031	71.33972	76.91976
Antigua and Barbuda	0	0	0	0.9078580	2.5649510	7.0473447	15.952980	27.714385	34.585416	41.432231	46.413654	59.372604	62.089403	64.02155	68.53730	70.03467	72.18616	63.11240	64.80076	71.08294
Argentina	0	0	0	1.6713326	6.3822279	20.1667847	47.311377	60.833778	61.968874	56.344602	56.248329	54.471914	51.297595	49.85310	46.70853	46.35849	49.22181	49.17324	48.07166	44.42367
Armenia	0	0	0	0.6259820	1.3744996	4.1702559	13.661545	23.425223	36.578528	39.844977	36.274422	39.650043	36.958818	38.55631	40.26395	35.78804	32.25286	22.30483	25.73802	31.67891
Australia	0	0	0	0.2125328	0.9406160	5.2046862	11.357834	15.543992	16.180806	15.288672	15.280714	18.362214	18.888925	22.85746	23.67735	24.83913	27.35088	18.12177	15.21642	17.59435
Austria	0	0	0	0.1657869	0.5105661	2.9402116	7.589992	12.114608	14.998789	15.812270	14.357658	17.699552	17.427628	18.15490	18.78498	19.32870	21.97880	19.51543	19.80850	21.43855
Azerbaijan	0	0	0	1.4219742	2.3648448	4.5633522	11.310999	17.000019	23.488210	24.958178	28.637441	31.305585	29.024301	30.10680	29.87631	28.50059	27.70376	24.05886	28.17994	28.09692
Bahamas	0	0	0	1.1310852	4.3160453	13.7240085	31.142287	48.258287	42.034188	48.775148	52.268570	54.518690	52.064532	54.72382	50.45411	50.99204	56.08344	45.10086	42.33006	40.12994
Bahrain	0	0	0	0.9743584	1.7477599	1.6211376	3.424752	5.800640	7.583210	7.773504	8.112449	16.768785	15.694317	18.68039	24.25011	24.25640	29.11272	25.89803	21.62462	24.19409
Bangladesh	0	0	0	0.3560024	1.5733852	3.7344021	7.447702	12.954127	19.834989	21.501961	30.813879	28.980178	26.174079	24.48911	23.94313	19.79483	21.38187	24.56404	22.17029	27.04544
Barbados	0	0	0	1.6297087	5.5719626	12.5768734	28.407449	45.310325	50.595694	52.314543	57.197024	62.953456	75.021370	72.29127	76.94418	81.57689	86.10729	82.30630	84.85090	88.99782
Belarus	0	0	0	0.6736708	1.9570439	7.2913226	18.773306	31.895491	36.648027	34.823917	32.969945	37.216976	32.708891	30.94909	29.55449	25.98970	24.22352	20.98665	20.71456	24.67119
Belgium	0	0	0	0.1412320	0.5756811	2.7439234	7.821393	13.605935	16.560180	18.955192	16.431495	16.207238	16.019971	16.15994	16.53715	17.69492	19.75917	21.22235	21.57199	21.50793
Belize	0	0	0	1.7136829	5.4522595	18.8589695	48.978042	56.289917	61.176398	82.889687	84.951299	84.558405	82.097209	76.00374	82.46560	88.21502	81.79935	70.28629	70.95312	80.23968
Benin	0	0	0	0.4704119	3.3321533	12.1934092	21.852328	33.527692	51.576322	56.088448	68.791771	85.978499	93.970528	89.16419	97.64054	96.24780	85.90043	92.50155	69.09549	78.72902
Bermuda	0	0	0	0.4194583	1.5383290	3.9896139	8.717858	13.853159	15.858672	17.166342	17.534195	18.638086	18.288622	19.21219	21.16630	24.04745	27.72652	27.64891	30.42296	38.84424
Bhutan	0	0	0	0.2546999	1.4601933	3.8536920	10.022088	17.204862	26.463137	30.162466	31.417203	35.331464	33.381257	32.35984	35.99449	33.83260	32.60672	36.55446	25.92992	28.60308
Bolivia (Plurinational State of)	0	0	0	0.7516180	2.3304569	6.8030047	20.854652	38.907248	61.138363	82.218687	98.533028	118.668876	125.049774	145.41582	161.94960	165.65099	163.47412	147.94611	104.18854	93.69905

Supplementary Table S6. Age distribution of incidence (per 100,000) for Cervical cancer in different countries in 2019.

Bosnia and Herzegovina	0	0	0	0.3380364	1.1650859	4.0577713	12.695733	23.981488	28.611343	38.972244	34.586907	30.264369	29.459770	27.96975	29.56609	30.50598	25.84366	21.05720	17.39925	18.48615
Botswana	0	0	0	0.9489878	3.6295435	13.3226749	35.661447	66.739771	95.544610	108.136783	116.150539	125.567857	118.726385	126.07706	136.38601	135.28147	136.94878	132.63934	102.74263	113.65596
Brazil	0	0	0	0.8423966	3.0437089	9.7082007	22.293579	30.105183	34.533955	35.470946	36.048493	39.288420	38.308920	41.01168	41.71254	44.08821	44.69286	45.67045	42.46030	49.90052
Brunei Darussalam	0	0	0	0.7817030	2.2146246	8.1739726	24.049428	39.809713	45.299819	45.427912	55.080205	60.460620	65.965874	77.81557	77.51969	71.24955	66.34614	79.14844	58.68701	54.18787
Bulgaria	0	0	0	0.5575678	2.7072097	9.0631560	28.805282	44.943957	59.366722	62.476567	60.011703	54.768262	44.668684	38.45065	31.02546	26.79864	25.47355	21.80418	17.32350	18.68968
Burkina Faso	0	0	0	0.5065806	3.7921198	14.4598161	27.284397	40.546109	62.791123	65.659671	76.355762	94.716607	105.636714	98.62027	108.14204	108.04481	93.88819	96.64366	70.65736	76.82331
Burundi	0	0	0	0.4838322	3.6615594	11.2995303	26.525253	47.389085	73.126200	88.206816	103.870953	106.790112	114.743957	107.95268	104.44677	69.54834	85.80448	87.52773	55.35440	59.13287
Côte d'Ivoire	0	0	0	0.4300484	3.1494473	11.3093357	21.188819	32.057600	50.079853	51.842949	60.802268	77.189656	86.796813	84.01080	91.77985	94.63920	86.57451	92.60723	66.58167	76.87426
Cabo Verde	0	0	0	0.7691727	3.2775906	8.7352941	14.379677	22.383977	38.385968	39.714651	41.538064	59.570920	67.767441	68.80370	85.41989	92.33906	98.10853	136.47794	106.99860	111.93659
Cambodia	0	0	0	0.8086320	1.9003692	4.4125082	11.728069	21.592438	35.486741	40.471703	50.687207	54.538946	49.161969	47.54150	46.84629	41.28437	36.73691	35.35586	25.78133	21.02371
Cameroon	0	0	0	0.4778717	3.6345736	13.3494099	25.548882	39.505954	60.058544	62.216839	70.641495	88.594784	98.781923	93.15497	102.52229	100.80492	91.92022	96.77238	70.21066	75.92610
Canada	0	0	0	0.3040116	2.5161340	10.0655646	20.877508	24.681252	25.613050	22.480002	19.196759	19.936014	19.051622	19.48424	19.37981	19.55789	21.17514	15.55179	16.98347	23.63870
Central African Republic	0	0	0	0.8390242	3.0409072	11.3906806	29.350660	50.004763	71.927562	95.079378	110.521709	128.300434	114.144817	111.67296	105.51552	97.23002	90.66093	87.70303	70.80667	62.59342
Chad	0	0	0	0.4245872	3.1825816	11.6617580	22.701706	34.505304	55.402387	67.596940	87.301601	110.087749	119.005099	110.78706	119.87862	109.50396	96.20499	97.43857	69.66226	73.78883
Chile	0	0	0	0.6317186	2.3866719	10.6536441	27.118848	35.836388	36.438125	36.394452	32.986046	33.534511	33.699211	40.20027	41.07337	47.37327	52.37931	57.13958	61.59350	56.74454
China	0	0	0	0.5186462	1.4827348	3.7544066	9.992365	16.676284	23.241892	24.944473	28.526790	29.828638	27.068781	25.97847	25.74414	24.50593	22.87823	21.12547	17.70942	17.90128
Colombia	0	0	0	0.6672815	2.7569675	10.5623188	24.400095	34.948906	38.505213	36.269607	38.413131	40.397758	42.619067	42.12363	44.49666	45.76449	44.83430	44.53054	37.59590	39.13355
Comoros	0	0	0	0.3795912	3.7604717	11.3264659	24.590999	45.162590	67.741050	78.966876	89.590601	92.839119	103.382212	98.95260	97.81602	66.95909	81.85938	86.37911	53.54328	60.61554
Congo	0	0	0	0.6716477	2.8127763	10.5530157	28.644187	49.790514	70.331999	84.265565	95.669628	108.798675	100.275430	100.29752	96.63384	93.80033	89.91351	96.82317	84.98328	82.25970
Costa Rica	0	0	0	0.3778112	2.0891182	9.1201190	20.842561	34.903671	35.964765	33.510994	32.022154	31.168563	33.927290	36.54900	37.27922	41.11105	47.06598	50.83382	46.57111	56.84680
Croatia	0	0	0	0.1623519	0.7923618	4.4033171	11.360338	17.622355	24.245225	28.277412	30.128872	30.545453	31.307526	32.95120	29.70843	30.54864	33.45800	28.24862	28.20665	30.68673
Cuba	0	0	0	0.5724797	2.9020879	10.5272744	23.364472	34.336078	40.030225	39.741867	41.914969	45.250184	44.509443	45.10085	43.64220	43.27252	41.66065	32.71559	33.48096	30.31849
Cyprus	0	0	0	0.2664479	0.4854924	2.0422782	4.713168	7.128121	15.855199	10.822332	14.949397	14.232721	14.240407	18.02963	17.80629	24.94483	30.48862	31.83063	31.46049	42.19727
Czechia	0	0	0	0.2390582	1.4703808	5.1998323	16.550165	20.566870	24.455998	23.813611	22.480199	24.433115	24.254363	25.44217	25.17693	22.68533	23.42628	25.99612	21.05023	17.97884
Democratic People's Republic of Korea	0	0	0	0.8178410	2.2296640	6.5308355	15.817990	26.064095	37.150344	49.320903	52.791054	56.912358	48.128834	41.78978	33.91462	29.86443	24.34999	20.52201	14.30823	14.58478

Supplementary Table S6. Age distribution of incidence (per 100,000) for Cervical cancer in different countries in 2019.

Democratic Republic of the Congo	0	0	0	0.7036274	2.6903043	9.0724866	24.508885	39.595676	52.578865	67.784439	85.183589	99.850448	94.702735	93.93275	91.62577	83.48302	81.96694	80.44122	70.83008	75.25159
Denmark	0	0	0	0.1965386	1.7881566	5.5657885	13.130017	16.667551	20.207500	17.000220	16.875792	17.843345	17.750707	17.19679	20.33693	23.35361	26.25006	24.01329	21.45985	23.19410
Djibouti	0	0	0	0.3019034	2.4528945	8.1365287	21.187492	38.906778	62.640037	79.382643	90.738407	94.563892	104.072022	102.09981	100.02039	71.76756	90.03345	91.75525	57.24574	63.64926
Dominica	0	0	0	2.0428623	7.4296611	18.0410876	41.614069	56.178466	64.131280	74.307430	52.279066	62.247065	79.936868	85.55979	86.46791	100.40207	105.53549	92.16023	96.53436	101.25991
Dominican Republic	0	0	0	1.3871328	4.4172572	11.8117425	26.129775	45.374552	46.538575	48.745347	57.561231	55.672311	61.750030	55.72079	68.90576	60.27869	77.02472	98.91129	88.48387	96.54671
Ecuador	0	0	0	0.8138694	2.1834166	5.9121863	21.703871	37.569634	48.601211	55.298751	61.059957	67.864202	72.563244	77.18243	80.77604	87.47699	93.94120	112.43367	119.86685	142.98860
Egypt	0	0	0	0.1563091	0.2913932	0.6754487	1.667771	2.828654	3.496065	3.903242	4.996936	7.561642	8.567680	11.13565	12.29514	13.52297	14.72275	13.63250	11.76443	11.39264
El Salvador	0	0	0	0.8762115	2.9230256	8.8224889	27.565159	44.483585	57.041594	63.544650	68.536071	76.861548	71.862676	72.53988	75.29060	82.60329	86.45949	88.73948	70.46610	67.40573
Equatorial Guinea	0	0	0	0.5885758	2.3638204	8.5561076	22.869624	35.810050	47.706216	57.588367	64.470550	74.925834	73.164972	79.95597	83.20161	86.48505	90.79120	92.92226	78.77224	79.71953
Eritrea	0	0	0	0.3436426	2.8600530	9.7561993	24.266225	52.760341	87.288370	115.362440	128.737686	132.469993	135.189787	125.25027	122.66071	81.41730	97.85185	108.17651	67.27978	65.71773
Estonia	0	0	0	0.7949591	2.1727544	6.9371379	18.383682	29.581069	34.270745	39.580835	37.186356	41.641483	44.377520	35.44205	34.45863	32.29476	30.13916	23.73683	22.70402	27.39205
Eswatini	0	0	0	0.8415106	2.8847747	9.4673078	26.002262	48.428847	73.946464	91.275135	101.899435	110.164129	104.031710	118.45783	130.17079	136.30041	144.57502	147.86127	115.73285	116.82610
Ethiopia	0	0	0	0.2572552	2.0900059	6.4921178	14.518294	25.025885	35.743032	48.898742	62.636108	69.059344	81.778884	83.16823	91.39536	70.43192	77.76514	74.32720	44.11026	48.64999
Fiji	0	0	0	1.3714053	3.3405692	11.8977413	33.406538	58.004952	89.232175	84.890992	94.731733	114.706981	92.462358	108.58447	111.86501	88.87913	80.45970	153.89758	146.33513	149.10048
Finland	0	0	0	0.1456921	0.6192546	2.6239368	5.507112	7.079600	8.671830	9.033128	9.220794	11.656309	12.190887	16.31219	17.37479	19.52139	22.06750	16.40247	14.92684	14.77266
France	0	0	0	0.1894361	0.7200484	3.1331976	9.704965	14.963574	19.465743	19.901041	18.694923	17.487285	15.066045	15.80362	16.17189	17.08661	19.62515	20.17602	21.11260	22.97689
Gabon	0	0	0	0.5905617	2.1787690	7.4697878	20.894755	34.495343	47.387680	57.543591	64.819790	73.691731	71.468258	76.93037	78.41454	80.92127	84.38485	90.59287	81.23960	85.13780
Gambia	0	0	0	0.2392757	1.8045866	7.8275430	18.273895	32.207133	50.891588	56.369074	68.346145	77.152147	77.064267	66.18029	73.61854	69.61115	61.88012	69.95301	43.16110	41.90395
Germany	0	0	0	0.1997138	0.9164134	4.5560129	11.173356	16.217660	19.537409	19.063715	20.044809	22.284460	21.198636	20.70521	21.52337	23.32548	25.81819	23.56201	21.13137	21.56886
Ghana	0	0	0	0.4432706	3.5672497	12.8084923	24.342956	35.486232	55.178258	51.970411	57.811378	70.259137	78.211078	76.41840	83.42877	83.68180	78.23826	86.13599	59.89717	64.92982
Greece	0	0	0	0.3288513	1.1103999	4.1690087	10.350380	13.125638	17.959942	18.963129	19.352128	18.839873	16.241800	16.55746	17.25994	18.76150	22.12243	24.39882	28.08578	27.02260
Greenland	0	0	0	0.5962974	3.3342772	18.2266327	38.252342	53.343646	55.294910	53.930024	47.717370	49.991832	45.611144	45.29716	43.24757	42.45193	46.13324	43.91563	35.67144	30.80079
Grenada	0	0	0	1.5594757	5.4284540	14.9866828	40.308711	63.419768	58.407911	59.834412	69.372024	82.698755	89.933536	93.01220	93.70706	87.96300	90.06854	82.79276	79.78913	81.82654
Guam	0	0	0	0.5566885	1.0991778	3.6417858	11.591231	24.285318	35.686735	31.128326	34.532640	32.970719	27.115448	32.42955	26.77490	28.90925	24.51847	42.33748	37.46577	34.62066
Guatemala	0	0	0	1.5686678	3.5161056	9.5482038	24.835415	41.471142	56.022085	67.801171	75.146565	85.495228	83.622208	85.23659	84.00297	77.58373	85.54495	115.30200	114.84085	97.94663
Guinea	0	0	0	0.6063938	4.4989109	17.4395663	37.584876	66.227173	105.865572	116.015114	136.947862	145.021958	150.110196	151.33268	161.46050	149.16025	129.34966	132.73995	90.45773	98.24110

Supplementary Table S6. Age distribution of incidence (per 100,000) for Cervical cancer in different countries in 2019.

Guinea-Bissau	0	0	0	0.4925800	3.9057979	16.3243554	33.085549	56.120648	91.403178	95.932336	103.852008	131.272204	133.612606	118.98637	125.52235	115.81274	98.66696	110.44179	77.22231	73.06704
Guyana	0	0	0	1.7207354	7.1697332	19.8095561	42.660223	64.520372	66.554011	80.185450	87.447941	102.133247	99.393463	95.83349	99.11100	89.76559	84.22875	69.30951	81.59234	93.25718
Haiti	0	0	0	1.9106732	7.1322609	20.6199261	43.019602	68.727711	83.029899	91.390600	100.761002	114.276129	111.525471	111.63669	118.17945	110.89392	110.58739	88.13602	78.48079	71.67307
Honduras	0	0	0	0.6099246	1.7846149	5.4511616	15.226667	26.125299	38.103000	41.346471	45.773340	52.168564	49.162566	50.86569	52.22490	49.67766	50.43289	47.65429	50.87604	62.64354
Hungary	0	0	0	0.1735106	1.2843792	5.1656714	15.431064	27.669715	30.401522	38.626756	35.759818	37.066707	31.993820	28.86802	23.61116	21.84150	23.17520	22.12564	18.20107	15.39674
Iceland	0	0	0	0.1482818	0.7955958	3.1051973	7.598621	11.279293	12.552073	11.596529	11.333434	11.447779	11.273704	12.63949	13.59550	15.33665	16.96062	14.44008	13.33516	16.88595
India	0	0	0	0.4398094	2.4193722	4.9978248	11.231025	17.660931	26.250926	27.998712	37.644381	36.726894	34.793395	31.15984	30.66453	25.89362	26.90970	26.46722	23.60075	26.34863
Indonesia	0	0	0	0.5995660	1.3056750	3.0478342	8.063894	14.914029	24.696333	28.546780	34.538441	38.183880	35.063955	35.14770	36.58331	34.18177	32.14482	33.30476	25.71449	21.77149
Iran (Islamic Republic of)	0	0	0	0.4748013	0.7079676	1.5549947	3.152665	4.714537	5.810027	6.213807	7.117905	10.136164	10.502572	13.01720	14.63068	16.92041	18.92655	15.31934	13.16432	15.54897
Iraq	0	0	0	0.2523631	0.6421101	1.3472674	2.952684	5.773783	8.149692	10.016877	11.951221	13.696218	10.498842	12.44072	13.67107	14.28807	16.18927	12.35937	10.18212	9.46646
Ireland	0	0	0	0.2013039	1.0978526	5.9483523	13.988866	21.725731	24.545766	18.702846	20.412835	20.947163	17.468059	17.27137	16.73733	16.11379	16.58743	15.70728	13.18002	14.25635
Israel	0	0	0	0.2180925	0.6332634	2.9882504	7.283856	13.876202	13.727667	12.894528	12.907284	14.992374	14.309295	15.32567	16.37175	18.11619	18.79797	17.19418	19.20942	25.49105
Italy	0	0	0	0.2875092	0.8084890	2.9243131	7.616596	12.094320	14.822435	14.960343	16.221873	18.215461	17.652074	19.57493	20.92287	23.43846	26.52399	18.37790	15.73551	20.28987
Jamaica	0	0	0	1.0718658	4.6469938	12.9144775	36.414667	60.103490	67.998850	78.345857	72.339937	81.071780	90.905551	90.51363	78.55023	74.05525	71.63565	58.76131	51.56064	67.95188
Japan	0	0	0	0.1984403	0.8709362	4.7786516	15.172202	23.586603	26.656511	27.396511	26.742447	22.706431	19.333077	19.55309	20.40096	21.61785	24.55816	18.98169	16.63840	18.89707
Jordan	0	0	0	0.1519769	0.4145204	1.0632559	2.869786	5.459297	6.585988	7.033801	8.387379	10.253094	11.512151	12.90136	13.49529	15.02406	16.02870	14.12344	12.61594	14.75773
Kazakhstan	0	0	0	0.8576604	2.0217439	7.6891261	23.252283	38.417779	48.650928	45.658760	41.668413	38.678442	36.155304	35.09902	32.46860	29.45356	27.96756	20.80080	22.32766	32.10579
Kenya	0	0	0	0.1162769	1.2098208	4.1570498	10.856536	21.288888	34.314225	44.330307	50.466552	50.137479	52.287737	52.01733	55.45484	41.36651	51.71559	54.33905	31.25025	33.28838
Kuwait	0	0	0	0.3133105	0.3417418	0.8668917	2.305168	3.775333	4.877144	5.927285	6.125675	10.491925	9.628752	14.73899	14.27962	15.72967	17.71086	12.98575	10.74008	13.50122
Kyrgyzstan	0	0	0	0.7422066	1.5502173	4.3873671	16.447463	29.513871	40.885414	48.719207	47.324723	44.745875	45.594747	44.78023	39.45906	36.28845	33.18078	21.85937	22.50048	20.34847
Lao People's Democratic Republic	0	0	0	0.7719435	1.7696553	4.2686429	11.583397	20.072248	32.640624	35.103169	43.045876	46.879623	42.301749	40.06241	40.11371	35.12311	32.38718	30.47332	22.24435	18.43512
Latvia	0	0	0	0.1943615	0.7587615	2.8600505	8.369547	11.793068	17.197459	19.319127	20.849727	22.706365	22.468907	23.16731	23.44818	21.33227	22.94996	18.39366	20.34801	23.93224
Lebanon	0	0	0	0.1885994	0.7792100	2.6016583	6.377824	10.110666	12.759578	11.906091	12.496258	15.138279	14.276746	15.67393	16.13109	15.73125	17.00795	14.72878	12.28114	13.65527
Lesotho	0	0	0	0.8487862	3.0272719	10.4355713	29.616231	58.187921	91.550269	121.910311	139.435700	155.092853	146.182220	154.28528	163.79029	163.28455	163.78940	164.72130	127.07538	121.69622
Liberia	0	0	0	0.4300526	3.5279744	12.1729495	22.105980	34.515529	52.928200	59.245682	71.356843	90.415798	96.567930	90.54028	97.44726	95.78774	88.79182	95.58028	68.66858	73.69156
Libya	0	0	0	0.1629703	0.7547310	2.5995669	7.343203	11.912141	16.522803	18.351985	18.872005	22.582972	23.464685	24.64609	21.02675	20.05531	17.44231	15.36087	13.29340	13.71557

Supplementary Table S6. Age distribution of incidence (per 100,000) for Cervical cancer in different countries in 2019.

Lithuania	0	0	0	0.2681036	0.8000580	4.3413669	13.088044	17.961235	23.431815	27.639585	29.745075	32.924844	34.807175	35.12456	28.54283	27.81057	28.56425	25.98050	23.11479	21.04914
Luxembourg	0	0	0	0.1676520	0.7000568	2.8008558	7.321473	10.280620	10.956447	10.705095	10.088591	10.651766	10.578723	12.08053	13.04962	14.07491	15.48462	13.46206	13.88428	16.57288
Madagascar	0	0	0	0.3770754	3.2723288	10.9257120	25.636988	44.978213	66.845646	79.405406	90.485882	93.540451	97.810169	88.66041	86.46966	56.63742	68.15634	72.41648	45.37737	48.03979
Malawi	0	0	0	0.5068866	4.1667165	14.1670945	33.632006	54.352941	74.555914	86.862720	101.995752	104.773981	115.706445	108.14796	110.85755	80.34441	98.69091	101.40365	64.38540	76.02007
Malaysia	0	0	0	0.3723293	1.2220463	3.2748704	9.988639	18.021632	28.436236	35.067486	43.612586	52.054421	53.474326	56.52458	63.89591	64.57577	68.72828	70.16503	47.26380	40.87335
Maldives	0	0	0	0.3836475	0.7489765	1.6913311	5.167048	9.660566	15.108509	17.080460	20.629702	23.899206	24.749966	26.59665	29.74887	31.90728	35.47747	36.41458	27.51575	24.15506
Mali	0	0	0	0.2926574	2.0576060	8.4138373	18.985110	32.881195	50.639294	60.592626	72.527037	84.564256	89.204298	80.17719	83.28340	73.69984	63.22042	57.28864	33.79846	35.33145
Malta	0	0	0	0.2045528	0.6871809	2.6706458	6.677124	8.944915	10.088606	9.197195	8.939987	10.340147	10.384994	12.48274	13.37700	13.86920	14.71723	11.95565	10.21000	11.15649
Marshall Islands	0	0	0	0.8120810	2.9735158	11.8949417	32.055831	60.646118	95.039887	86.036444	92.710626	104.511244	71.551459	88.89932	93.16985	77.80378	71.66108	117.83062	105.19846	105.84071
Mauritania	0	0	0	0.3284830	2.5820563	9.1000186	17.876857	27.209783	45.013464	51.331531	64.587892	82.054749	92.394483	88.43562	96.73572	95.32187	88.11912	97.20926	68.03161	72.34808
Mauritius	0	0	0	0.3215613	0.7772960	2.5031210	8.017408	13.150392	25.615802	22.311346	26.307880	33.997839	36.962608	36.26029	41.72895	38.26163	40.74026	33.43451	31.04322	16.92911
Mexico	0	0	0	0.4888907	1.8343051	7.2691436	18.339013	27.241676	35.118224	37.490896	39.866940	44.002962	45.604746	48.76838	48.02280	53.00262	57.25754	63.07844	60.51390	63.62025
Micronesia (Federated States of)	0	0	0	0.7815156	3.1895864	12.4605131	32.904606	51.822646	70.271924	78.707027	94.664308	107.344674	75.540550	91.07556	97.40862	81.60154	76.53563	124.97553	113.03803	113.63693
Mongolia	0	0	0	0.1922476	0.6452132	4.2973357	15.585842	28.516213	43.067380	49.688188	51.616996	49.971298	56.840411	59.26227	54.29480	55.74982	64.41047	65.40775	80.58803	94.56578
Montenegro	0	0	0	0.2899883	2.2731036	6.9900785	18.910427	26.609334	32.005519	30.727414	32.099537	30.815810	26.743257	26.08327	23.78783	21.75659	22.13645	19.13740	15.73151	15.53529
Morocco	0	0	0	0.2626361	0.9471790	2.2870219	5.957232	13.832802	23.111334	33.810377	41.947387	51.667602	41.214535	41.18510	37.88647	34.79952	33.82347	28.38041	22.15574	19.40909
Mozambique	0	0	0	0.5065235	3.8804087	13.5685261	31.693082	54.800538	80.037023	100.097725	113.834476	116.915208	126.376611	118.82879	121.33013	89.61255	111.78294	123.83436	76.58478	78.37616
Myanmar	0	0	0	0.8836760	1.9401998	4.2961831	11.512945	19.533765	29.762997	31.493095	37.104343	40.107138	36.653485	35.50142	37.07773	33.85987	31.46146	29.20999	21.53467	18.45454
Namibia	0	0	0	0.3841562	2.0087605	6.7753686	18.361907	32.771715	47.903412	56.893459	66.403840	69.889873	76.060606	81.85110	91.59368	89.63910	78.72009	74.99840	47.77395	48.28952
Nepal	0	0	0	0.2871109	1.5882758	4.1766381	10.736076	18.627030	29.110755	33.951841	36.959968	36.892235	36.382990	33.37723	35.78589	32.70887	29.59083	34.59576	28.29504	29.34165
Netherlands	0	0	0	0.1700760	0.8287713	4.3513781	11.769899	14.148261	14.748160	13.469687	13.195791	12.651751	12.018148	14.52028	15.58115	16.48174	18.86614	18.78032	21.01119	19.02119
New Zealand	0	0	0	0.1270534	0.6361211	3.7586709	7.470915	10.229170	12.330519	12.864200	11.733346	13.644421	13.586100	17.16735	18.87307	19.59764	21.14867	16.05911	13.10336	14.06914
Nicaragua	0	0	0	0.5965892	2.2563791	7.2615525	20.795099	37.517821	52.706008	62.979792	66.742090	70.593589	79.280952	89.39432	93.88153	110.20523	116.58116	120.88872	92.39341	88.24256
Niger	0	0	0	0.4642527	3.2341237	12.1007423	22.622810	34.292244	53.614392	62.131114	81.755963	101.825603	111.766572	105.65969	116.23782	110.70808	95.78808	99.03441	70.32253	75.92326
Nigeria	0	0	0	0.1307869	1.3126931	6.2860242	13.453732	20.833600	29.802411	35.324223	41.812070	49.394313	56.338153	56.89581	61.00062	62.26149	54.49291	57.84563	38.26744	41.26829
North Macedonia	0	0	0	0.3946615	1.8918053	6.0596663	13.408818	24.350925	30.145962	36.196601	33.551330	37.073955	33.873409	34.03259	30.21088	36.12408	31.08262	28.26567	21.34948	18.03199

Supplementary Table S6. Age distribution of incidence (per 100,000) for Cervical cancer in different countries in 2019.

Northern Mariana Islands	0	0	0	0.8597064	2.5735027	9.0178464	28.495166	55.255536	87.602756	77.312731	86.395151	96.649383	74.837544	93.67732	97.79136	81.38896	78.53789	136.48294	135.24559	156.46703
Norway	0	0	0	0.2116420	1.3754947	4.3267251	10.439677	16.166377	16.831889	16.506603	16.069220	16.026420	16.490925	17.81621	17.83392	19.98183	19.21940	22.41620	19.90533	24.07170
Oman	0	0	0	0.1800784	0.6105178	1.6815340	3.713269	6.290648	7.822142	8.316456	10.566263	16.406729	16.221700	22.32555	27.02081	29.55978	29.94575	21.41477	16.72513	16.15763
Pakistan	0	0	0	0.1395410	1.1041355	3.3625573	7.828706	11.996305	15.698266	16.951723	18.431939	19.726574	19.659497	18.40778	19.98307	16.74459	15.46766	14.91643	10.39262	11.27878
Palestine	0	0	0	0.3173322	0.8893950	1.4885514	2.566573	4.185540	4.300918	5.318077	7.103335	12.106554	13.323560	20.24077	24.47989	26.51597	26.44917	20.14643	16.55999	13.15932
Panama	0	0	0	0.7484508	3.2785188	13.7907409	33.906007	40.033175	48.526153	44.796358	43.161980	48.595662	47.732109	50.03547	49.31955	50.05811	50.42564	57.55645	46.24244	54.89471
Papua New Guinea	0	0	0	0.6418616	1.7980430	6.8001962	17.948310	33.933950	55.599684	48.875956	58.135603	66.390663	49.162928	64.33462	65.86035	54.62770	48.87087	75.15884	66.14307	62.33202
Paraguay	0	0	0	1.0054944	3.8998057	14.0706028	36.924858	60.545089	75.717066	79.595659	76.883995	77.585599	79.151001	75.28848	72.65036	79.48342	84.13045	84.17308	65.95639	82.99082
Peru	0	0	0	1.2746331	2.8374397	6.9862357	19.665098	32.165843	47.339427	59.280573	66.597928	77.299257	75.795057	83.03298	86.90998	89.76733	84.63500	78.15105	65.53556	76.09756
Philippines	0	0	0	0.8253320	2.1581352	4.8229967	12.694571	20.384468	30.175645	32.849187	37.455897	40.548542	34.039787	31.14245	28.67600	25.06465	22.96864	23.72510	20.32000	16.88987
Poland	0	0	0	0.1744126	0.7240170	2.6289535	6.790399	12.982979	19.196160	24.633507	27.297237	32.423970	31.008843	29.05064	27.04207	25.31027	27.24720	28.71041	23.41183	19.81070
Portugal	0	0	0	0.2974997	0.8021104	2.9320612	9.859851	16.651418	22.152144	26.419624	24.397161	24.151170	23.377843	25.10882	25.95643	25.87985	27.67134	22.65266	22.16331	26.77623
Puerto Rico	0	0	0	0.3012525	1.9016730	7.8877659	16.988299	23.306078	24.416257	22.285102	20.120254	20.639835	20.078278	20.98283	21.99651	21.85863	21.34644	18.66361	18.50326	20.99720
Qatar	0	0	0	0.3848555	0.7404724	1.8512782	4.683440	7.249625	7.093374	7.699324	9.665241	18.948695	24.710965	32.47316	39.53269	45.29969	82.03766	84.22028	50.67827	57.89921
Republic of Korea	0	0	0	0.2431755	0.8541143	3.9931865	12.232120	18.284840	18.706088	17.526186	18.126606	16.858361	16.571335	19.90577	22.74606	26.42676	33.24107	32.15938	27.95807	28.48167
Republic of Moldova	0	0	0	0.7117212	2.0161015	7.4597217	18.182168	26.947942	36.686005	38.347966	31.432151	33.299295	32.402301	30.65485	27.70192	23.44512	19.64120	13.91274	11.70092	9.62434
Romania	0	0	0	0.7242564	3.2279808	10.9864430	28.909800	47.039468	60.231271	66.408837	73.950066	72.451380	66.654335	58.12641	51.03072	46.28236	37.37412	28.86485	18.61142	15.37757
Russian Federation	0	0	0	0.2324644	1.5832221	8.6887900	24.772817	36.932562	41.069161	37.409796	32.908373	30.335570	27.808665	25.70134	23.00825	20.48901	19.41051	15.39146	14.58814	16.34438
Rwanda	0	0	0	0.4122534	3.3570664	10.3962532	24.299201	40.988894	57.720908	70.199648	85.100368	86.901408	96.027519	94.58255	96.39060	67.77163	85.06797	91.46737	58.49981	63.55985
Saint Lucia	0	0	0	1.0596553	4.1165756	13.8131743	31.860631	48.483718	59.639990	66.607406	59.680000	64.182380	63.091670	67.49650	65.57258	61.13964	68.67347	76.78918	91.28975	102.43470
Saint Vincent and the Grenadines	0	0	0	1.6719963	7.1457429	22.1920782	48.325832	69.306459	80.365799	93.020043	88.694660	88.455706	86.891396	88.09063	99.74231	102.48268	119.37064	107.65772	106.29420	104.48535
Samoa	0	0	0	0.2840594	1.6063470	7.6085549	21.995509	42.658217	64.417945	59.743427	65.398325	73.293270	55.457352	62.20200	58.28979	45.34655	36.98127	49.34604	37.00066	35.98000
Sao Tome and Principe	0	0	0	0.7128591	4.9071925	16.8588985	33.574168	54.701744	83.889891	92.467922	100.904211	120.575859	125.665055	120.94438	130.24947	126.33847	112.48923	135.57022	96.85157	100.38532
Saudi Arabia	0	0	0	0.1132120	0.4841261	1.5335909	4.489611	6.957238	9.047485	9.084612	9.702207	12.934902	12.009226	14.60130	16.28253	17.56825	19.56291	16.04835	12.72907	13.36086
Senegal	0	0	0	0.4139370	2.9646925	10.7262361	19.670055	30.121959	50.487529	54.693200	67.770768	86.899650	95.810791	92.58787	101.39791	99.56972	92.33231	100.30698	70.69633	72.35239

Supplementary Table S6. Age distribution of incidence (per 100,000) for Cervical cancer in different countries in 2019.

Serbia	0	0	0	0.2457383	1.4890865	7.2574639	22.561823	39.441052	51.877474	58.318481	57.573687	58.265375	51.255763	46.28009	36.77405	35.43679	42.37765	37.96328	30.94032	30.04467
Seychelles	0	0	0	1.6455778	3.8195655	10.3124722	26.287996	45.560376	65.552123	73.026094	83.878678	82.450196	82.214810	87.71543	92.78068	102.02697	100.10246	107.38192	97.98625	100.30679
Sierra Leone	0	0	0	0.4802637	3.6762471	13.7320980	25.572297	39.073654	61.267394	65.739375	77.939448	96.989302	106.361463	98.28543	103.84797	101.03312	90.81520	94.62515	67.51723	75.24734
Singapore	0	0	0	0.3066155	0.5715226	1.6861996	5.342708	8.518510	12.487917	15.032696	19.613034	21.872145	21.626877	23.79894	23.95664	26.60733	30.07605	22.10458	19.93106	22.39291
Slovakia	0	0	0	0.2013021	1.5215176	6.6135249	17.924749	33.605341	40.149543	41.719764	39.712574	39.512853	36.868268	33.53951	30.16550	29.52332	29.48643	21.61687	17.96089	18.55393
Slovenia	0	0	0	0.1782145	1.1075685	4.2464609	11.664961	18.368833	23.211096	23.424257	22.732361	21.895238	22.097910	21.42048	21.23751	21.75197	23.37572	17.45911	17.64791	22.84951
Solomon Islands	0	0	0	1.4770507	5.9096048	24.4791960	57.621250	103.696833	149.933632	133.015480	138.928989	157.100758	98.122819	117.73617	116.90487	93.12741	73.84249	109.41649	89.59924	79.53087
Somalia	0	0	0	0.3920910	3.2309392	10.5056126	24.129609	47.527656	76.202353	100.650230	121.725440	131.606218	132.317469	122.86677	115.69496	76.73063	89.00255	93.69388	56.55758	54.59115
South Africa	0	0	0	0.4217213	2.1594693	11.9045908	35.649506	46.940708	52.650882	55.645200	61.236347	69.522928	69.194227	78.87815	76.01656	74.23406	98.00353	108.74144	100.30381	118.04144
South Sudan	0	0	0	0.2651146	2.0817971	6.8446125	16.341148	29.111470	41.806045	53.849031	67.894246	72.143831	81.640779	81.34401	84.94663	58.93799	71.42435	69.93208	45.11569	51.33914
Spain	0	0	0	0.2035175	0.6776155	2.5369531	7.323268	12.101762	18.487150	21.451236	21.201085	22.317925	20.280387	22.32986	22.50247	23.58655	25.03050	17.29763	16.71692	18.10910
Sri Lanka	0	0	0	0.4317148	0.9205281	1.6228620	4.755552	9.093299	14.266292	15.698486	19.714428	21.269891	22.063147	24.24516	26.91632	22.26301	22.71903	25.64034	25.99381	28.26822
Sudan	0	0	0	0.2394505	0.6295485	1.6451767	4.116401	7.125276	9.106311	9.955614	11.639634	15.132718	14.742420	17.32045	19.19716	19.53836	20.58280	15.60718	11.43882	11.25474
Suriname	0	0	0	2.0454714	5.1687173	15.2401708	37.649220	66.308380	61.684279	63.736603	63.718990	77.191797	76.799580	71.62613	85.22745	85.66227	81.00642	67.90049	61.95881	65.88912
Sweden	0	0	0	0.2081289	1.2859883	5.0657485	12.384452	12.715694	14.374211	12.470669	11.903485	12.304935	11.814281	14.53589	15.03713	18.76338	19.86184	22.33172	25.17057	30.85406
Switzerland	0	0	0	0.1987983	0.6227483	2.4521683	6.349199	8.996781	10.737299	10.798351	10.876020	12.923275	12.960710	16.77380	16.72329	18.51570	20.15976	18.68438	16.55019	18.43617
Syrian Arab Republic	0	0	0	0.2672805	0.6611664	1.3028663	2.762731	4.128184	4.678481	5.157421	5.861651	7.809064	8.246547	10.06629	11.23339	12.37107	15.52709	18.10532	16.00442	12.12218
Tajikistan	0	0	0	0.2973968	0.7742242	2.3817403	6.455674	9.890232	12.809621	13.858368	15.461263	18.925712	21.375342	25.52751	28.64560	28.32534	26.53925	16.54440	14.18283	15.39846
Thailand	0	0	0	0.9618240	2.2558193	6.3223286	15.949766	26.383803	33.587693	33.387714	38.224736	40.198705	39.893378	40.60050	40.71409	40.15886	38.72601	38.12968	36.09425	36.50936
Timor-Leste	0	0	0	0.6497479	1.4422538	3.3101732	9.084439	16.207168	27.263047	33.722727	45.257303	50.846431	46.168325	44.62686	44.21661	39.44865	36.21909	34.33842	24.99156	20.22349
Togo	0	0	0	0.4353735	3.3844053	12.0234047	23.994734	35.276938	57.134359	58.365554	69.362866	85.112483	94.520365	90.63301	96.91273	96.03984	88.61951	94.18096	66.78742	72.60103
Tonga	0	0	0	0.6102512	1.9820180	6.9737598	22.961845	42.859936	68.077619	59.107996	69.637616	88.842805	71.193262	97.87881	95.96584	86.00373	83.12508	141.59824	145.49490	139.76968
Trinidad and Tobago	0	0	0	0.9312660	3.8431288	9.3809712	18.386202	33.441356	38.744672	40.907087	46.622016	55.737654	58.924888	67.77341	64.84683	69.95757	75.87729	59.50283	59.51639	72.42110
Tunisia	0	0	0	0.1249391	0.6144890	2.0067687	5.591357	9.132160	11.409003	11.094854	11.257598	15.002179	14.230952	16.31801	16.12285	15.57151	17.99298	14.74519	11.55331	12.37460
Turkey	0	0	0	0.1962694	0.4780403	1.0909499	3.111411	5.511158	7.353737	7.357793	8.454808	10.324511	11.343162	14.21953	18.09437	24.35151	31.57985	25.02160	17.93768	16.38477
Turkmenistan	0	0	0	0.9672167	2.2543331	6.4693976	16.747330	24.212606	39.693063	40.792243	43.035221	38.104700	36.691532	28.99072	27.01620	21.66441	19.56336	11.61714	12.47726	14.85808

Supplementary Table S6. Age distribution of incidence (per 100,000) for Cervical cancer in different countries in 2019.

Uganda	0	0	0	0.4644997	3.8060629	12.2969101	28.683726	50.636091	68.136020	78.582226	96.565387	96.399847	113.879432	115.05643	119.97398	80.71133	101.85417	102.19676	64.86843	74.01156
Ukraine	0	0	0	0.6259922	1.9504982	6.2062474	12.230311	17.712885	21.349545	24.266939	27.399626	30.617873	32.093849	34.77753	32.55542	26.57470	23.85132	16.27197	14.41775	15.48941
United Arab Emirates	0	0	0	0.1524942	0.9177012	1.3491425	3.055433	6.783116	11.166922	17.625463	23.290926	31.190986	35.051518	56.33607	47.92021	34.15028	45.66694	70.45715	66.76990	75.31908
United Kingdom	0	0	0	0.2815282	1.7891111	8.4520195	15.261485	17.368358	16.952715	14.836076	12.411435	13.674556	13.487719	15.23265	16.43514	17.98292	19.97936	18.26894	18.09551	22.83623
United Republic of Tanzania	0	0	0	0.4215514	3.3055720	10.4685704	25.474188	42.703999	61.090727	74.705473	93.795366	99.544640	109.120435	105.16851	105.36196	74.24436	89.42027	90.24745	57.74774	63.90353
United States of America	0	0	0	0.2190827	1.2046075	5.4121931	11.796607	15.901467	18.536052	17.651128	17.786435	19.378042	18.936476	19.30745	19.44004	18.25483	20.41863	14.65470	12.81856	12.47123
United States Virgin Islands	0	0	0	0.5376725	1.5707544	4.2067185	11.182888	21.115284	27.855185	33.088894	35.872208	40.614792	39.208149	42.19972	45.75455	49.86791	55.21821	51.24888	51.27656	54.38878
Uruguay	0	0	0	0.8077233	3.5792977	11.3971584	41.341557	47.892916	46.256031	43.569933	42.669727	44.196935	37.841251	36.22216	39.95757	41.73764	47.81900	48.40783	48.35506	44.29903
Uzbekistan	0	0	0	0.9780730	1.9526191	4.8640668	15.192081	26.635245	40.423160	42.138957	45.449727	45.327010	47.057263	36.30896	34.36566	31.49581	35.48386	25.14791	19.41417	22.60767
Vanuatu	0	0	0	0.6522188	2.0464749	8.1329301	21.803071	43.193046	68.657763	63.873337	70.548063	80.815507	57.202408	72.68430	76.73025	64.74644	59.03995	96.72105	88.01779	91.68577
Venezuela (Bolivarian Republic of)	0	0	0	1.7628325	7.2368796	26.8770027	52.785612	66.377683	71.250448	66.955539	66.651430	69.677973	69.634063	69.06881	73.94872	73.24294	71.42289	59.69538	52.38488	63.71816
Viet Nam	0	0	0	0.3364658	1.3003977	3.6588746	10.571244	18.482873	30.084498	35.007467	43.721611	53.249599	55.290957	57.74610	60.48500	56.09147	52.91659	52.76226	40.62605	39.73449
Yemen	0	0	0	0.1657748	0.5126204	1.4918090	3.816856	7.238450	11.389573	12.807460	14.458018	18.818414	17.966587	20.59067	21.94814	21.10518	22.29632	17.23151	12.90972	12.89153
Zambia	0	0	0	0.4926926	4.5079381	14.1959505	34.382825	62.369661	89.172937	102.824364	114.612273	116.001882	121.095411	111.35928	108.83735	74.16097	91.11821	100.91403	66.24866	72.40776
Zimbabwe	0	0	0	0.6863654	3.4216542	12.6949906	34.842764	59.355508	87.602391	106.769143	118.440873	132.317357	137.815489	148.44060	157.13248	149.15759	143.68973	136.48837	92.57814	87.16330

Supplementary Table S7. Age distribution of death rate (per 100,000) for Cervical cancer in different countries in 2019.

country	15 to 19	20 to 24	25 to 29	30 to 34	35 to 39	40 to 44	45 to 49	50 to 54	55 to 59	60 to 64	65 to 69	70 to 74	75 to 79	80 to 84	85 to 89	90 to 94	95+
Afghanistan	0.09569453	0.33718897	1.05800605	2.6930792	5.9003385	9.8825758	13.093944	16.795040	24.063849	22.582401	26.952385	31.788236	34.29250	39.11615	35.71659	31.48202	33.47882
Albania	0.05593641	0.35027699	0.74729650	1.5754632	2.5289629	3.6954686	5.080216	6.510859	6.720460	8.321702	9.851002	13.730162	17.39366	21.92168	19.77644	19.23585	26.79699
Algeria	0.03260421	0.15462489	0.56604732	1.5985968	3.3350701	5.6525447	7.177138	8.610669	11.186345	11.867410	14.139595	16.467202	21.35323	28.59169	60.66294	66.08663	38.06678
American Samoa	0.08150000	0.27906005	1.10666770	3.2611320	7.0492925	13.7818763	15.042473	19.852299	25.277266	21.194775	32.130308	43.403831	43.21333	48.21410	100.78338	118.73701	149.40928
Andorra	0.02224030	0.12653934	0.57878450	1.5370703	2.8413610	4.4641285	5.874092	7.904983	9.961311	10.836488	14.207946	18.336220	23.08995	28.83267	35.80841	42.80984	62.28327
Angola	0.18102900	0.77492476	2.80550699	7.7771436	13.9998049	24.3061588	33.197787	47.134406	59.136697	62.752709	71.631990	80.799636	86.85399	98.61063	107.32569	106.18746	135.04947
Antigua and Barbuda	0.12561212	0.39565495	1.17021497	2.8316072	6.1303066	10.0370231	15.112068	20.088806	28.229846	33.656803	41.248248	53.337089	63.39368	75.77076	80.66749	96.19397	124.74702
Argentina	0.19571492	0.82642767	2.84240821	7.1700270	11.7119815	15.9978738	19.298829	24.345081	27.321379	29.226148	33.224920	36.982862	42.53524	51.81058	62.71843	71.06674	78.03817
Armenia	0.08377574	0.20504926	0.67902068	2.3003893	4.8239153	9.7076585	13.653816	15.525015	19.547361	20.933707	26.025028	32.689943	34.03654	35.85720	28.98769	38.56276	56.11129
Australia	0.01379644	0.06799149	0.40843301	0.9562658	1.6437372	2.2682818	2.739467	3.415640	4.584040	5.322515	7.764612	10.273772	13.15148	18.17278	18.68618	19.81547	25.36295
Austria	0.01316264	0.04550063	0.28607652	0.7736896	1.5382565	2.5607101	3.859035	4.736072	6.889448	7.760225	9.613652	12.229363	14.84732	20.30173	24.00566	28.70131	36.78953
Azerbaijan	0.22777616	0.42331967	0.89021237	2.2708828	4.1681548	7.3196441	9.861167	13.809863	17.092424	18.040834	21.884329	25.665783	28.29090	31.65179	31.88336	42.81389	46.77186
Bahamas	0.17755997	0.75979231	2.42445846	5.8894312	11.3522701	13.2140459	19.156237	24.169814	27.635096	29.866067	36.734040	40.752515	47.40968	60.15701	57.89352	62.68809	75.57551
Bahrain	0.10690881	0.21380077	0.21595289	0.4748878	1.0154680	1.7646697	2.405723	3.205637	7.739478	8.316748	11.734118	18.463814	21.71467	30.40899	32.90383	31.90591	39.63599
Bangladesh	0.06491365	0.32101966	0.82511450	1.7362492	3.7233240	7.2698674	9.781749	16.775530	17.686358	17.726209	18.923207	21.341925	20.02881	24.37067	32.16508	33.26547	48.30807
Barbados	0.21961405	0.84202537	2.09641861	4.8464871	9.8205185	14.2775944	18.660138	24.218362	29.445678	40.119108	45.857256	59.199849	73.20117	89.66709	104.64147	125.67297	156.55684
Belarus	0.07724353	0.25058261	1.01801107	2.7326126	5.8397571	8.6075440	10.532324	12.839912	16.802958	17.199859	20.075503	23.266149	24.36193	26.07873	26.93146	30.64640	43.48353
Belgium	0.01023569	0.04647952	0.24097084	0.7328184	1.6615361	2.7912073	4.480667	5.242622	6.332045	7.389032	9.144167	11.697987	14.95976	19.93925	27.07238	31.90616	36.94911
Belize	0.28585654	1.08332285	4.09088712	10.4704611	14.5337893	21.2985077	34.662582	41.573238	45.195325	49.432949	53.228682	68.501444	84.12177	89.12063	90.51578	105.60364	144.73607
Benin	0.10812726	0.86076424	3.40920668	6.5326789	12.0899743	23.2692770	30.048777	42.620270	58.329745	70.070227	74.261363	91.963587	101.85219	100.87533	120.72438	102.30480	138.38381
Bermuda	0.04178541	0.16937957	0.48200778	1.0987838	2.1911771	3.3908970	4.758280	5.903830	7.058509	8.016644	10.270217	14.267729	19.15904	26.32043	33.63226	43.37072	65.97006
Bhutan	0.04368452	0.27991000	0.80251486	2.2056639	4.6565499	9.2188814	13.128644	16.534913	20.916897	22.083228	24.477116	31.696492	33.99725	37.27561	48.40748	38.92543	50.11136
Bolivia (Plurinational State of)	0.15916571	0.55192788	1.75373991	5.2886596	11.9619995	22.9488280	35.224086	48.808386	62.774708	71.896004	95.340017	122.400128	147.69962	171.45169	190.89801	154.88639	166.61780
Bosnia and Herzegovina	0.04165216	0.16011856	0.60698447	1.9450949	4.5139149	6.9559196	12.435489	13.881748	14.112644	15.907112	18.091444	23.329831	28.33198	28.41874	27.43122	26.01658	33.37135
Botswana	0.15858157	0.67948095	2.71513410	7.6868640	17.6990490	32.7057998	46.458445	60.469038	73.646158	77.865507	94.919512	119.140803	134.84110	154.67443	173.66187	153.39594	188.36834
Brazil	0.12913968	0.52139683	1.80566206	4.4170795	7.4454790	10.8431472	14.094266	17.342218	21.000144	22.866005	28.432290	34.469819	41.53323	48.34649	58.93942	63.47900	89.56959

Supplementary Table S7. Age distribution of death rate (per 100,000) for Cervical cancer in different countries in 2019.

Brunei Darussalam	0.08418109	0.26103648	1.04187727	3.2101785	6.7402583	10.2033778	13.548184	20.850383	26.346077	32.190696	43.925202	52.253603	55.17150	59.67220	95.86283	85.71586	84.91001
Bulgaria	0.07944525	0.43002091	1.56606856	4.8423539	8.7336971	14.1966112	19.052511	22.933569	24.313825	22.741462	23.545931	23.760568	24.12025	27.06041	27.92248	25.68934	32.19040
Burkina Faso	0.11791463	0.98351588	4.09816296	8.2261547	14.6186151	28.4451483	35.330263	47.583840	64.703633	79.014531	82.390943	102.162944	114.60309	110.47518	125.76634	104.59824	135.40726
Burundi	0.12014236	1.00821585	3.41153418	8.4819446	18.2656658	34.9521228	49.744711	67.330958	75.502561	88.408699	92.468916	100.372472	74.75382	101.97167	115.75229	82.72898	104.30670
Côte d'Ivoire	0.09988955	0.82187673	3.20508499	6.3691626	11.5998804	22.5269779	27.863406	37.723045	52.654699	64.968570	70.165662	86.669802	100.19389	101.58645	120.64785	98.35049	131.44960
Cabo Verde	0.11769184	0.55947954	1.62838242	2.8003771	5.4189171	12.0735050	15.882141	20.413137	33.168651	42.475896	49.827298	72.654134	90.20865	109.52059	178.88741	160.12793	200.78330
Cambodia	0.16081907	0.42131037	1.06597694	2.8406664	6.1707870	12.4914513	17.306979	25.484493	30.508184	30.651679	34.500284	40.489750	41.05269	41.81179	46.19218	38.52516	36.94981
Cameroon	0.10284834	0.87707896	3.49704783	7.1360517	13.3859319	25.7009148	31.914618	42.324453	58.523410	71.963471	76.128997	95.271382	105.47764	107.19572	126.29553	104.05208	133.52404
Canada	0.01306040	0.11959352	0.52185209	1.2015454	1.9139765	2.8557857	3.664424	4.438185	5.574832	6.331520	8.196186	10.696178	13.26800	17.84733	18.19293	23.77339	38.06702
Central African Republic	0.24615464	0.99770333	4.07464664	11.2219373	22.4804400	39.5103608	59.649565	77.524745	96.591079	93.044269	99.755428	104.835119	107.30845	109.85483	117.31301	107.48114	112.02555
Chad	0.10842457	0.90640804	3.62319959	7.4604591	13.7288828	27.2450523	38.802598	57.240653	78.493280	92.456072	95.383835	115.673112	118.02454	114.19635	127.22184	103.28675	128.88302
Chile	0.06110358	0.25863164	1.25647870	3.3919704	5.7354666	7.9107719	10.713890	12.529534	15.126092	17.406547	24.649110	30.520584	41.13314	53.10162	72.33550	90.78199	95.11147
China	0.06424715	0.20122847	0.55008817	1.5163606	3.1314158	5.7653538	8.355874	12.312962	15.223589	16.309262	19.224344	22.878737	25.26906	26.40532	27.72358	26.58459	32.43389
Colombia	0.08883551	0.40804882	1.70592953	4.2579315	7.6408223	10.8041830	13.177232	17.403403	20.439793	24.104910	27.787942	35.775289	41.73188	47.15956	56.64509	55.61016	70.82791
Comoros	0.08620772	0.95215250	3.13145212	7.2285746	15.9812353	30.0229623	42.052854	55.490919	63.153970	77.034042	82.463099	92.161738	70.78055	96.27927	113.65808	79.60297	106.50505
Congo	0.15100946	0.70678285	2.88057539	8.2675735	17.4907048	30.9589153	44.428619	58.696745	73.401828	74.144090	83.147420	90.839506	99.11819	105.75128	128.61878	128.94943	148.63126
Costa Rica	0.04676964	0.28547116	1.36050800	3.3696264	7.0437492	9.4611088	11.426118	13.786709	15.052962	18.370323	23.264995	29.105004	36.50236	48.58895	64.39618	68.98462	102.04916
Croatia	0.01223862	0.06692956	0.40467947	1.0476645	2.0124988	3.5675201	5.615003	7.783445	9.290083	10.756952	13.805345	15.562781	19.56362	26.58147	32.50054	39.12271	48.87628
Cuba	0.06752238	0.38128904	1.49998011	3.5514377	6.4359175	10.0530494	12.715891	16.095401	19.316789	21.927933	26.628706	31.742155	37.10337	41.79956	40.72321	48.04265	52.19311
Cyprus	0.01984307	0.04032321	0.18498864	0.4524170	0.8911878	2.7489659	2.628316	4.899777	5.691474	6.694937	10.398095	12.807807	21.41649	31.16654	41.32915	48.14859	71.80915
Czechia	0.02100901	0.14402421	0.55465270	1.8182389	2.9832120	5.0355409	6.853503	8.520898	11.309137	13.103506	16.491857	19.790358	21.00421	25.61931	33.49909	31.36333	31.16879
Democratic People's Republic of Korea	0.13859708	0.42069974	1.34489796	3.2274137	6.2822629	11.0689315	18.154041	23.247835	28.102399	26.762171	27.746877	27.552187	28.15973	26.66248	26.20661	21.08848	25.32330
Democratic Republic of the Congo	0.17360184	0.74250026	2.70906028	7.7606600	15.0955868	24.9519733	37.808352	54.810754	70.073514	72.507004	79.992289	87.726149	89.44251	97.15477	106.16743	105.78643	132.43708
Denmark	0.01389652	0.13984990	0.47356076	1.1676796	1.9276678	3.4027782	4.129387	5.857695	7.653835	9.010337	10.599284	15.531032	21.32595	28.05510	30.97245	31.92019	41.18501
Djibouti	0.06475995	0.58830699	2.11948325	5.8312039	13.0465043	26.6838586	41.047962	54.880269	63.195924	76.557666	84.026158	93.480981	75.25909	105.50044	121.33465	85.79091	111.99373
Dominica	0.34917333	1.41930560	3.77884068	8.9835332	15.0061972	22.5150010	31.778687	26.061543	33.640248	48.757516	60.474072	72.487370	96.57273	115.92166	119.50838	144.73234	176.99647

Supplementary Table S7. Age distribution of death rate (per 100,000) for Cervical cancer in different countries in 2019.

Dominican Republic	0.23242041	0.82505748	2.40507294	5.6174264	11.9798898	16.0852144	20.566210	28.191564	29.792372	37.201153	39.094621	57.235924	57.49328	85.16938	132.47993	137.05818	168.26297
Ecuador	0.13989733	0.41767794	1.21289064	4.2924489	9.3690816	14.9111343	19.979378	25.986526	30.993393	36.463259	44.692734	54.661787	70.72965	90.94909	139.47220	174.44830	239.54667
Egypt	0.02363203	0.04911968	0.12413199	0.3212732	0.6781418	1.0840864	1.548404	2.435532	4.185554	5.332241	8.028375	10.409735	13.13311	16.29823	17.78634	17.75611	18.97597
El Salvador	0.13297444	0.49460008	1.62514268	5.5137730	11.0643376	18.0950990	25.399749	33.767923	41.878369	43.639434	50.747722	63.085950	78.08588	93.97515	115.22739	106.37184	123.81031
Equatorial Guinea	0.11340296	0.50992587	2.01876721	5.6752083	10.8908268	18.4420650	27.372025	36.362045	47.065024	50.961018	63.149291	75.508873	88.90204	105.05274	122.93553	118.54579	143.37183
Eritrea	0.08585506	0.79941457	2.95649706	7.8840752	20.4723728	41.9650010	64.926625	83.425049	93.570305	104.151546	107.146970	117.899449	87.66364	116.64869	144.19773	102.25465	119.10339
Estonia	0.06007209	0.18505667	0.66208885	1.7505334	3.5588991	5.8015466	9.123291	11.453920	14.129026	16.884530	16.807194	21.191938	24.24768	27.58964	28.71613	32.51911	45.73581
Eswatini	0.17809751	0.68274573	2.43725804	7.1333660	16.0821737	30.9939122	46.231671	60.652161	72.310337	75.454871	96.415101	120.675848	142.46408	169.02812	196.08988	175.30226	211.52430
Ethiopia	0.05877072	0.53148330	1.79102481	4.2901440	8.8999662	15.8994662	26.089794	38.952863	46.959419	61.096652	69.383798	86.263870	74.62958	91.74852	97.97559	65.62509	85.24603
Fiji	0.27204414	0.74053981	2.87417151	8.0530015	16.4626694	31.2080400	36.039482	47.454066	63.749026	57.379335	78.398990	96.430756	87.89248	90.95905	200.20165	219.39444	261.69245
Finland	0.01011064	0.04792014	0.22078487	0.4807503	0.8043572	1.2746495	1.822594	2.434815	3.769901	4.675243	7.524550	9.847518	13.17215	17.47260	17.97110	20.22251	23.33328
France	0.01348465	0.05725473	0.27150481	0.8967934	1.7969781	3.2496846	4.626909	5.887253	6.813291	6.866069	8.864457	11.336768	14.38668	19.66100	25.74414	31.33369	39.41501
Gabon	0.11654557	0.47921579	1.79514726	5.2975181	10.6808836	18.6783673	27.736098	37.034085	46.820777	50.184117	61.249646	71.417871	83.42451	97.53616	119.39623	121.73581	149.64318
Gambia	0.05323837	0.45273930	2.11623773	5.1995224	11.1506414	22.1611039	29.431126	41.651288	51.682073	56.870542	54.667791	68.891633	73.29954	72.51071	92.59597	65.14380	74.91881
Germany	0.01337087	0.06852686	0.37226502	1.0407695	2.0372704	3.3644717	4.399498	5.697362	7.201168	7.750731	8.982441	11.687778	15.36312	21.10746	27.08901	30.46167	37.48843
Ghana	0.08945373	0.80100829	3.13438828	6.3147462	11.1992989	22.1589516	25.505718	33.488981	45.151978	55.500607	61.263789	76.487736	86.59037	90.54360	112.89511	89.14590	114.48266
Greece	0.02432844	0.09144076	0.37263331	0.9856413	1.6305159	3.0775848	4.533581	6.229030	7.407543	7.527116	9.388045	12.184526	15.82028	22.18165	30.81410	41.13768	47.82179
Greenland	0.05678576	0.35302911	2.10070017	4.7518849	8.5942642	12.3086158	16.574601	19.412558	24.115730	25.515704	30.127410	34.396423	38.96483	48.35842	55.32574	51.92153	51.00723
Grenada	0.24741096	0.95913418	2.86026818	8.0381430	15.9070447	19.2659215	24.012680	32.599063	42.585664	52.373093	63.486920	76.500064	82.62532	97.08647	106.28893	118.55918	146.69980
Guam	0.08183321	0.18016451	0.64923163	2.0543311	5.1430272	9.5020022	10.455077	14.134264	15.290495	14.226299	20.409712	20.924361	26.47947	26.32156	53.24407	53.86137	68.39218
Guatemala	0.29332248	0.73456974	2.16056253	6.1412002	12.5207468	21.2324091	31.535455	41.945133	52.030463	56.020095	64.776912	74.680324	77.20291	96.15196	151.27856	174.90654	172.23970
Guinea	0.15054796	1.25317534	5.30915077	12.1089673	25.7578521	51.0929823	65.529298	88.659558	102.186733	115.359174	129.360427	154.892149	159.79343	153.02365	173.43073	134.22849	171.20901
Guinea-Bissau	0.12417487	1.09708612	4.96381586	10.6996783	21.6598771	43.2656608	52.865296	65.394620	89.771548	100.401073	99.242418	118.073330	121.84687	115.27937	143.19732	113.91314	128.58323
Guyana	0.33032710	1.54132990	4.71034353	10.6676480	19.5708143	25.9748624	37.222431	46.546651	58.896184	63.921443	70.884867	85.886289	88.61594	93.99547	89.98384	122.45679	166.35231
Haiti	0.48254378	2.00181719	6.30582326	13.9842954	26.4885307	40.2756998	50.823123	62.587862	75.580886	81.030884	91.268190	109.961153	115.99664	128.74480	115.49744	117.12721	129.26167
Honduras	0.11488849	0.37608398	1.25062483	3.7771296	7.9184858	14.5114722	19.369518	25.634779	31.838500	33.078689	38.805367	46.690395	49.89545	58.16436	64.89749	78.91212	121.38849
Hungary	0.01844129	0.15221100	0.66639919	2.0361800	4.4745886	6.4577659	10.847822	12.892435	15.665930	15.833231	17.326605	17.631557	19.43412	24.81261	28.43903	26.98268	27.25227

Supplementary Table S7. Age distribution of death rate (per 100,000) for Cervical cancer in different countries in 2019.

Iceland	0.01017511	0.05967401	0.25384191	0.6586694	1.2781386	1.9390830	2.537434	3.357287	4.154269	4.780323	6.631246	9.045854	12.24169	16.31035	17.68935	19.25175	29.06193
India	0.08587742	0.52530137	1.17716132	2.7806618	5.3343596	10.0527682	13.071190	20.860674	22.677391	23.760323	24.189464	27.398238	26.20775	30.61269	34.51540	35.40404	49.03929
Indonesia	0.11813406	0.28866292	0.73490927	1.9442088	4.2315864	8.6277339	12.052040	17.174777	21.096974	21.588743	25.220224	31.392930	33.70352	36.37237	43.47012	38.47917	38.37510
Iran (Islamic Republic of)	0.05570440	0.09221303	0.22117455	0.4677908	0.8837127	1.4402947	2.026902	2.933031	4.846173	5.764264	8.416516	11.413107	15.34103	19.91994	19.58359	19.57069	27.65304
Iraq	0.03502495	0.09925413	0.22697535	0.5198034	1.2777614	2.3507096	3.726056	5.532296	7.246393	6.274383	8.645641	11.248371	13.54964	17.68494	15.86588	15.30355	17.06591
Ireland	0.01359465	0.08237842	0.48615914	1.2162998	2.4694256	3.8553140	4.149361	6.165144	7.774816	7.640048	9.346035	11.423926	13.20076	16.29152	19.74873	19.27483	24.33829
Israel	0.01732170	0.05600672	0.28674527	0.7435395	1.8390792	2.4997834	3.263251	4.400772	6.194741	6.912176	8.990108	11.907279	15.67390	19.18966	21.83158	28.33501	44.77903
Italy	0.01878814	0.05960138	0.23325767	0.6436974	1.3105447	2.1906116	3.032535	4.288026	5.553708	5.896648	8.058202	10.564441	13.30700	17.87404	20.48216	21.50153	31.41779
Jamaica	0.16066671	0.79072621	2.49844416	7.2252171	13.7190839	21.4195192	30.238028	32.788623	40.238850	51.293721	60.014928	62.737598	68.44585	76.48477	75.55988	77.57844	124.23877
Japan	0.01257112	0.06140070	0.36703145	1.2234430	2.4460539	3.7764938	5.348800	6.915338	6.912240	6.690613	7.967757	10.212662	12.82592	17.74160	19.71435	22.67515	28.53434
Jordan	0.01789221	0.05419638	0.15170451	0.4270819	1.0247214	1.6375869	2.297781	3.474774	4.922145	6.303475	8.358199	10.547274	13.64999	16.91421	18.07405	18.78210	26.33454
Kazakhstan	0.11993355	0.31591858	1.30991611	4.0488291	8.1862938	13.3360670	16.163407	18.322289	19.566326	20.911869	24.071202	26.717646	28.28811	31.25384	27.17557	33.25743	54.63045
Kenya	0.02911263	0.32914659	1.14019036	3.0716246	7.3184700	15.1777545	23.966480	33.096894	37.558036	44.342703	48.962560	58.128585	46.83731	64.54837	79.85860	53.78889	66.77868
Kuwait	0.02945661	0.03583301	0.09902958	0.2760728	0.5722943	0.9855468	1.619123	2.166064	4.377328	4.646460	8.617113	10.314880	13.42509	18.07560	16.67450	15.92897	24.63791
Kyrgyzstan	0.11877898	0.27874236	0.85501183	3.2726945	7.1425871	12.6819901	19.156303	22.822748	24.483259	28.326173	32.500447	33.870514	36.00257	38.15995	29.90135	35.55148	44.81417
Lao People's Democratic Republic	0.17238527	0.43888959	1.15797292	3.1605485	6.4459121	12.8030249	16.444326	23.375703	28.063431	28.077360	30.513106	35.904060	35.83352	37.40358	39.84198	33.08750	32.22849
Latvia	0.02858685	0.12575042	0.52016014	1.5360114	2.7375591	4.9794076	7.167991	9.748045	12.203650	13.920937	17.300597	20.429249	21.77734	26.24403	24.04176	30.24725	44.20989
Lebanon	0.01819557	0.08375730	0.30476606	0.7812067	1.5756846	2.6500411	3.322832	4.514712	6.452929	7.013928	9.275527	11.722684	13.43712	17.11856	18.44859	17.91416	23.19817
Lesotho	0.19832067	0.78798784	2.95502992	8.9102428	21.2023545	41.6666885	65.912006	87.459208	106.397834	109.934604	129.554882	155.378190	174.06117	194.34848	220.24539	194.24385	221.35604
Liberia	0.09768984	0.89469988	3.34999783	6.4720447	12.2567741	23.4086007	31.360409	43.830273	61.008080	71.505994	75.066400	91.540106	100.99318	104.06633	125.00804	101.88576	129.21217
Libya	0.02207358	0.11416247	0.42739623	1.2698689	2.5892525	4.6579564	6.707968	8.584007	11.800154	13.846550	17.035887	17.264575	18.97012	18.77190	19.59156	19.49179	25.82305
Lithuania	0.03515111	0.11680142	0.69252345	2.1809664	3.9047684	6.0280046	8.570639	11.442938	14.860000	17.919140	21.668908	21.750198	25.22168	30.28087	33.19610	34.18580	36.67901
Luxembourg	0.01234234	0.05686498	0.24816829	0.6849709	1.2553080	1.8685238	2.563062	3.300875	4.275602	5.004042	7.031006	9.483948	12.29657	16.14362	17.87780	21.24111	29.47743
Madagascar	0.09224540	0.89102014	3.26672729	8.1446404	17.2082042	31.8227387	44.506772	58.459260	65.803099	75.190331	75.675810	83.016159	60.85650	81.00337	96.12157	68.38111	85.40027
Malawi	0.11808422	1.08488348	4.02242302	10.1835321	19.8195212	33.8211613	47.161554	64.087841	71.936684	87.010817	90.816352	105.070549	85.52551	116.89892	134.11540	96.33842	130.81652
Malaysia	0.05182674	0.19025195	0.55529504	1.6742901	3.6095849	7.1437139	11.155793	16.995325	23.154820	27.155704	34.524423	48.736618	58.07460	73.19132	89.41676	69.78981	73.27853
Maldives	0.04734923	0.10334202	0.25461615	0.7678120	1.7219257	3.4253954	4.949029	7.376466	9.778302	11.628263	15.207497	21.717499	27.74897	37.28120	46.71302	40.47466	43.81933

Supplementary Table S7. Age distribution of death rate (per 100,000) for Cervical cancer in different countries in 2019.

Mali	0.06850475	0.53577053	2.39146066	5.7069621	12.0022013	22.9388772	32.664341	45.442942	58.029497	67.007841	67.188380	78.819272	78.27724	74.41466	74.68806	50.05057	61.56239
Malta	0.01539549	0.05583950	0.23965010	0.6318251	1.1062836	1.7023612	2.174681	2.857343	4.048029	4.796637	7.097370	9.403981	11.58977	14.60645	14.76854	14.73540	19.85239
Marshall Islands	0.18282043	0.74414890	3.24843823	8.8095940	19.5472713	37.3956816	40.350970	50.557863	62.613048	47.640140	67.933986	83.580410	79.52642	83.06950	155.23379	158.38175	185.50852
Mauritania	0.06506098	0.57138717	2.18139556	4.5711488	8.5120840	17.9732857	24.821068	37.116793	52.339636	65.159793	70.469464	88.235148	98.28191	101.74004	126.97127	100.74752	126.89321
Mauritius	0.04436384	0.11888045	0.41732251	1.3613886	2.6371772	6.3868641	7.068926	10.227013	15.031163	18.640401	22.063185	31.756070	34.28278	43.07447	42.35476	45.50843	30.47899
Mexico	0.07291527	0.30387402	1.31052829	3.5731700	6.6281888	10.8755848	14.771497	19.365172	23.668220	27.283190	33.713706	39.857704	49.64982	61.51617	80.91641	89.82614	110.97678
Micronesia (Federated States of)	0.15919460	0.72341869	3.08392998	8.1526640	15.1176110	25.1459277	34.081020	48.308478	60.783199	47.547486	66.696529	84.843478	81.60191	87.64034	164.42923	170.53773	196.44129
Mongolia	0.03528452	0.13222421	0.95866344	3.5960604	7.9543057	15.2009594	21.715003	27.043651	29.413894	37.586165	45.318171	48.394328	56.89273	74.89993	86.38734	123.18071	171.06757
Montenegro	0.03165654	0.27655280	0.92697238	2.5563505	4.4051955	6.9608023	8.840137	11.814282	13.261446	13.416216	15.862829	17.932086	19.44927	23.76566	24.59905	22.98948	25.45483
Morocco	0.04111478	0.16490614	0.43510255	1.1879149	3.4283491	7.4231617	13.794910	20.992843	29.281001	26.198144	30.159730	32.489272	34.18898	38.34113	38.14846	34.22944	39.46799
Mozambique	0.12301381	1.05217821	4.00091591	9.9774043	20.8120926	37.8657945	55.792727	72.995623	81.926772	96.773865	101.116256	116.198894	96.06890	132.90380	164.31046	114.83728	139.38371
Myanmar	0.18085595	0.44146605	1.06428266	2.8668625	5.7265747	10.7468342	13.771894	19.040735	22.718409	23.195910	26.007839	32.326506	33.87468	36.00267	38.16699	32.03703	32.26933
Namibia	0.07109691	0.41507427	1.52107979	4.3837416	9.6136632	17.9146130	26.305862	36.591729	43.086847	52.105955	63.658669	82.149960	91.50028	90.69064	99.22359	71.85891	84.72206
Nepal	0.05588170	0.34607080	0.98664232	2.6754972	5.6675765	11.3289598	16.234726	20.976861	23.305746	25.463072	26.456860	32.552913	33.68087	34.18254	45.65150	42.62208	52.53833
Netherlands	0.01135227	0.06142405	0.34991901	1.0092039	1.5940039	2.2850556	2.950050	3.958279	4.645682	5.225679	7.822378	10.544198	13.45634	18.48669	23.54336	30.71940	32.73320
New Zealand	0.01514782	0.08165274	0.51902278	1.0764977	1.7792753	2.6094421	3.584524	4.037373	5.328784	6.009645	8.327944	10.852159	13.60221	18.00222	18.89643	18.40842	22.93018
Nicaragua	0.08808091	0.37109047	1.30258112	4.0268414	9.0667171	16.2110026	24.728271	32.333854	37.912563	47.417052	61.877781	78.464987	104.67021	130.96505	167.20627	153.78412	185.20867
Niger	0.11337238	0.88761181	3.63785828	7.1840104	13.2012002	25.5845277	34.705533	52.265531	71.197350	85.377198	89.778540	111.103503	118.56381	113.33993	129.29654	104.23189	133.30211
Nigeria	0.02991457	0.32627687	1.68018912	3.7854394	7.1598604	12.9344890	18.491832	25.549839	33.077952	41.307149	46.681365	56.899405	65.58505	64.01366	76.09579	57.18619	72.84898
North Macedonia	0.04776340	0.25540474	0.89138518	2.0164128	4.4637489	7.2441810	11.363980	13.312515	17.118211	18.055724	21.761690	23.654933	33.43502	34.20909	36.70980	32.18421	30.97177
Northern Mariana Islands	0.11313333	0.37848515	1.44545230	4.4909739	10.4022606	20.8778215	23.515795	32.314178	41.305863	36.459058	55.398849	72.886211	71.74504	81.82234	171.49072	196.78885	257.17921
Norway	0.01460400	0.10603262	0.36345065	0.9183884	1.8607410	2.7101041	3.780071	5.008869	6.119650	7.513976	10.170853	12.891672	17.64762	20.51548	29.32292	29.75458	43.05096
Oman	0.01901386	0.07178932	0.21550973	0.4972252	1.0731718	1.7702615	2.507348	4.083092	7.391833	8.438695	13.809248	20.332700	26.03175	30.78106	27.21035	24.94113	28.32015
Pakistan	0.02983850	0.26172611	0.86531790	2.1299662	3.9652412	6.5846508	8.612631	11.013153	13.005437	14.276129	15.009228	18.570444	17.48828	17.98283	19.48778	15.44998	19.74450
Palestine	0.04260752	0.13314695	0.24253690	0.4413097	0.8995264	1.2098522	1.945799	3.223993	6.303287	7.852121	13.954323	20.033770	25.06150	28.80269	26.12118	24.99601	23.20204
Panama	0.10005276	0.49540683	2.35285990	6.0235724	8.8783765	13.8917488	16.296225	20.051113	24.784623	27.249617	33.183715	39.749622	45.63562	53.19131	73.30234	68.40603	99.34099
Papua New Guinea	0.15491347	0.48645050	2.00079365	5.3121282	11.7519624	23.3177224	24.203106	33.107015	41.381644	33.754268	50.448063	60.095947	56.54566	56.91536	98.43277	98.84899	114.64226

Supplementary Table S7. Age distribution of death rate (per 100,000) for Cervical cancer in different countries in 2019.

Paraguay	0.15662137	0.67510505	2.65394356	7.4780480	15.1020859	24.0290957	32.060637	37.352437	41.788522	47.666133	52.563924	60.303275	75.12665	90.96397	107.85358	97.47792	149.49461
Peru	0.20760265	0.51616389	1.38371830	3.7418782	7.6261800	13.7174521	19.817847	26.111024	32.372924	34.759531	43.707100	53.715076	67.13959	76.36419	93.64857	92.30859	123.46294
Philippines	0.15446672	0.44495333	1.08730167	2.8421079	5.4398718	9.9548285	13.328566	18.044962	21.750950	20.490242	22.010811	24.410780	24.59698	25.90860	30.75094	30.37031	30.65314
Poland	0.02580235	0.11923119	0.47223956	1.3453045	3.2610264	6.2467566	10.248578	13.956888	18.598518	20.078055	22.610003	24.960043	27.08494	32.80781	38.16820	35.49719	36.22171
Portugal	0.02395804	0.07221533	0.29624851	1.0188387	2.0905521	3.6617953	5.616505	6.722554	7.998918	8.217772	10.458955	13.603318	16.71481	21.86514	26.31685	31.16437	43.49437
Puerto Rico	0.03194365	0.22452786	1.03012782	2.3409076	3.9582532	5.6768964	6.559729	7.186508	8.228809	9.254743	11.773172	15.381096	18.07291	20.96392	23.17930	26.95849	37.07389
Qatar	0.03681115	0.07899496	0.21539105	0.5705667	1.1236992	1.4652207	2.138197	3.469231	8.042014	12.072805	19.094468	28.580447	38.65455	84.04197	108.50703	75.86320	94.81398
Republic of Korea	0.01651817	0.06449619	0.32790994	1.0516675	1.9987726	2.7868906	3.601071	4.904531	5.363176	5.971677	8.511901	12.019084	16.35454	24.80608	34.92808	38.20536	45.06962
Republic of Moldova	0.10311938	0.33547220	1.32993206	3.3358005	6.3600562	10.4801117	13.987123	14.216067	17.309627	19.233890	21.860643	23.413778	23.17459	21.95529	18.20709	17.96904	21.10911
Romania	0.08504980	0.42406354	1.58422626	4.2431400	8.6010478	14.5138483	21.264913	30.033745	35.127213	36.461705	37.632873	40.049206	42.53982	40.66753	37.28986	27.57002	26.89040
Russian Federation	0.03036832	0.23454306	1.41217786	4.0771233	7.2389944	10.0844336	11.710870	12.803128	13.749348	14.580508	16.292359	18.121512	19.58296	21.17762	19.79395	21.62598	28.99184
Rwanda	0.09033684	0.81820997	2.75508393	6.8353184	14.0783960	24.8970610	36.454992	51.619608	58.065679	70.525996	77.795719	90.093170	71.26313	99.81333	120.65161	87.34354	111.93937
Saint Lucia	0.16026537	0.70039532	2.54559041	6.2424437	11.6967360	18.7892005	25.919812	27.284813	32.142951	35.858576	45.129856	52.388299	56.41939	72.68572	97.59900	134.57812	177.33183
Saint Vincent and the Grenadines	0.27680209	1.33545338	4.41778534	9.9140051	18.3332446	27.9889102	39.172996	43.581694	47.399637	52.383430	61.768227	83.058065	97.91841	130.39397	138.91114	157.46372	182.21249
Samoa	0.05074343	0.32124734	1.65934515	4.7860658	11.0356852	20.6108196	23.597071	30.780492	38.587310	32.711105	43.133613	48.837475	43.86905	41.14115	63.59013	54.64437	61.35541
Sao Tome and Principe	0.13697186	1.05414592	3.96161646	8.3055271	16.5326751	32.2152788	43.691649	56.859948	75.517903	87.426557	95.197098	118.027448	129.59144	129.41726	178.78518	146.21640	179.13704
Saudi Arabia	0.01245205	0.05944555	0.20522843	0.6298471	1.2269261	2.1136254	2.836837	3.860887	5.975085	6.368152	9.228132	12.479210	15.67939	20.29606	20.31613	18.55099	22.30152
Senegal	0.09290908	0.73693836	2.91602867	5.6771826	10.4998898	22.1217293	28.848801	41.434822	58.453266	70.917194	76.684488	95.183008	105.03690	108.33928	131.69199	104.95650	129.19542
Serbia	0.02466124	0.16624204	0.88373494	2.8036152	6.2277309	11.0865367	16.678964	21.390776	25.424370	25.818112	28.900809	29.878186	35.52685	47.68720	49.99341	47.27517	51.82450
Seychelles	0.24379244	0.63231749	1.85842696	4.7043410	9.7894328	17.6907154	24.687299	34.553770	38.302714	43.366992	55.547118	72.518502	93.72376	107.89995	136.88954	143.08333	167.82190
Sierra Leone	0.11148176	0.94796584	3.85490849	7.6274733	14.1073279	27.6559967	35.265619	48.638320	66.337263	79.617600	82.211374	98.055842	106.91741	106.45004	123.10072	99.83816	130.86721
Singapore	0.02101198	0.04359685	0.13984793	0.4628301	0.9410792	1.8953833	3.130980	5.356100	7.020098	7.862268	10.257910	12.744202	16.55881	22.54481	24.00054	26.90743	36.70989
Slovakia	0.01752768	0.14753613	0.69994941	1.9494431	4.5736206	7.2243429	10.056422	12.168537	14.258067	15.918253	17.412763	19.340530	22.17323	26.61567	26.08200	25.72506	32.41312
Slovenia	0.01140004	0.07875206	0.32997348	0.9292593	1.8313332	3.2433964	4.697188	6.116583	6.830062	7.984550	9.855616	12.858943	16.20614	21.67744	21.09902	25.23740	38.92269
Solomon Islands	0.31770678	1.40714617	6.34592397	14.9313148	31.6279717	56.4616893	60.387568	73.425788	91.592904	63.822442	88.295308	103.556352	94.83102	85.64765	146.28068	136.38942	141.71555
Somalia	0.11529221	1.06958828	3.86962820	9.6546548	22.6055000	43.5463307	65.186617	86.530397	99.603781	107.893597	109.862082	114.997161	84.79014	107.91614	125.74731	85.83398	97.55885
South Africa	0.07482219	0.42765717	2.55517827	8.0699716	13.1539701	18.9369850	24.869359	32.937245	41.865278	46.442401	60.417609	67.403590	74.80735	112.00406	143.27636	151.65925	214.31052

Supplementary Table S7. Age distribution of death rate (per 100,000) for Cervical cancer in different countries in 2019.

South Sudan	0.07205160	0.63434095	2.27284585	5.7132281	12.1713727	21.5190060	32.016598	45.461547	52.314041	64.305736	70.745795	82.487096	63.83091	85.50619	92.32436	67.62464	90.55112
Spain	0.01410874	0.05231168	0.21322558	0.6474394	1.3290400	2.6504651	3.963248	4.902180	5.936558	6.098660	8.136934	10.677845	13.78205	18.81216	19.75381	23.18390	28.72619
Sri Lanka	0.05534424	0.13162799	0.25251721	0.7319075	1.6831106	3.3407422	4.705166	7.294948	8.979593	10.655203	14.250941	19.904287	19.56443	23.69997	32.33422	38.15739	49.05673
Sudan	0.04191395	0.12124887	0.33124428	0.8522820	1.8321395	3.0937922	4.302889	6.140532	9.078317	9.914801	13.328663	17.082193	19.77149	23.52782	20.46044	17.02124	19.73836
Suriname	0.36528897	1.07479140	4.07370241	8.5273651	18.2107876	22.4448647	27.964728	32.583943	42.755376	47.850062	51.602926	72.261420	83.11416	89.39723	87.89478	91.08337	114.51002
Sweden	0.01468429	0.10108515	0.43457426	1.1435842	1.5579171	2.5116674	3.219872	4.345566	5.707602	6.624406	10.059867	12.547853	18.17904	22.08938	29.18923	37.78786	55.51897
Switzerland	0.01345393	0.04669840	0.19990772	0.5545793	1.0266560	1.6874245	2.393376	3.282197	4.780623	5.669673	9.099008	11.376534	15.13736	19.78680	23.43446	24.22516	32.95329
Syrian Arab Republic	0.03457547	0.09536084	0.20467744	0.4581312	0.8553511	1.2711732	1.826840	2.598769	3.987792	4.778160	6.837835	9.080585	11.56772	16.72015	23.47326	24.20154	20.17212
Tajikistan	0.05777228	0.16758576	0.56201825	1.5814973	2.9250823	4.7601256	6.302437	8.421029	11.494235	14.541047	19.978641	25.939629	29.27801	31.12686	22.08283	21.53632	25.59729
Thailand	0.12435637	0.32522490	0.99394821	2.4847314	4.9118239	7.9104923	10.050693	14.161964	17.085788	19.390974	23.963557	30.261439	35.40706	40.62770	48.42946	52.33408	66.42012
Timor-Leste	0.13918761	0.34297855	0.86300803	2.3898719	4.9742358	10.2407751	15.203238	23.766991	29.595082	29.863245	33.397939	39.056310	39.83764	41.69593	45.04422	37.44715	35.73911
Togo	0.09546875	0.82574774	3.21157534	6.8281901	12.0905787	24.6745262	30.388326	42.016760	56.782748	69.293624	74.623482	90.535547	100.96000	103.62389	123.34298	99.09439	127.77896
Tonga	0.11194564	0.40852240	1.56308636	5.1724853	11.3982262	22.3122114	23.832244	33.256138	47.358554	42.683363	68.688449	80.781447	83.30696	92.23573	180.90257	214.05576	263.98643
Trinidad and Tobago	0.14408672	0.66682663	1.77520028	3.6982212	8.2092203	12.5615569	16.384363	21.676139	28.389992	33.897662	45.610519	52.264550	65.19956	81.13278	76.14355	88.23910	128.51813
Tunisia	0.01404708	0.07689572	0.27322834	0.7998989	1.6389826	2.7208674	3.518388	4.531886	7.014982	7.613223	10.393240	12.396468	13.95775	18.76183	18.61457	16.89543	21.71645
Turkey	0.02198873	0.05968842	0.14844132	0.4398707	0.9829305	1.7414980	2.315360	3.373806	4.808017	6.044130	8.983989	13.831312	21.72237	32.84024	31.91764	26.04376	26.55638
Turkmenistan	0.15804076	0.41223953	1.28753418	3.4242025	5.9638637	12.5193818	16.287575	20.925579	21.084438	22.960784	21.212458	23.350577	21.57919	22.33590	14.99886	18.38904	26.80735
Uganda	0.10272818	0.94581848	3.32531721	8.1987986	17.5585527	29.7467768	40.984601	58.941606	64.699597	83.789343	95.097977	112.428660	85.20832	119.79756	134.45837	96.57080	130.75227
Ukraine	0.11054628	0.38075948	1.27886539	2.5861246	4.4250009	6.6061084	8.744689	11.499853	14.356783	16.678865	20.425414	22.500760	21.46037	22.72240	19.85242	20.78822	26.22678
United Arab Emirates	0.02127718	0.14226812	0.22846105	0.5458927	1.5141472	3.2356203	6.594919	10.849682	16.549926	21.058959	39.342746	39.329529	32.19524	49.36389	89.69834	97.30756	120.57795
United Kingdom	0.02243701	0.15902564	0.81652810	1.6395403	2.5556124	3.5626915	4.429476	4.856502	6.299165	7.129776	9.542469	12.484610	15.66208	20.23466	22.65516	26.33925	39.10329
United Republic of Tanzania	0.09382702	0.81805190	2.82969210	7.1873490	14.7150828	26.3361754	38.916647	56.861714	66.552826	80.221088	86.592690	98.581069	78.10959	104.92047	118.48659	85.70300	112.99621
United States of America	0.02293917	0.14023643	0.68585424	1.6027803	2.6678049	4.0517269	5.029442	6.380217	7.990477	8.632956	10.080956	12.296905	13.68939	18.62048	17.53586	18.22923	20.99115
United States Virgin Islands	0.07584176	0.25559688	0.79557301	2.0485233	4.7458866	8.1717227	12.148038	15.511586	19.331827	21.308857	27.051852	35.500345	44.98789	57.52726	64.96354	75.08593	93.71397
Uruguay	0.08879509	0.43788766	1.51233060	5.9651207	8.7122947	11.2471838	14.196897	17.768323	21.551413	21.010642	23.593551	31.165802	37.80751	49.88106	61.91021	71.81979	78.31451
Uzbekistan	0.15778296	0.35062033	0.95176550	3.0829586	6.5038524	12.6332468	16.674986	21.998112	24.887400	29.281788	26.431616	29.605117	31.30857	40.64890	33.15065	29.32111	38.33719
Vanuatu	0.15404524	0.53837840	2.32986613	6.3242187	14.6023570	28.3369468	31.135887	39.833065	49.804972	38.921848	56.703408	69.668741	66.84442	68.76287	127.33989	132.35735	160.59847

Supplementary Table S7. Age distribution of death rate (per 100,000) for Cervical cancer in different countries in 2019.

Venezuela (Bolivarian Republic of)	0.25387432	1.16264839	4.71947365	9.9353281	15.6302520	21.4597610	25.824336	31.678267	36.852105	41.153201	47.230785	60.803210	68.19629	76.56853	75.96026	77.55330	113.97760
Viet Nam	0.04908663	0.21142261	0.64843459	1.8503842	3.8741199	7.9008417	11.595425	17.659707	24.394362	28.734543	36.113475	46.925592	51.03759	56.74282	67.40500	59.79763	68.78407
Yemen	0.03224633	0.11124184	0.35356374	0.9517441	2.2079999	4.3911937	6.075547	8.101161	11.770141	12.452015	16.186744	19.814953	21.58575	25.59636	22.55147	19.20425	22.57401
Zambia	0.10684984	1.08475777	3.73758479	9.5936611	21.0634543	37.9204545	53.113805	68.966266	77.089757	88.491967	91.348321	101.414281	77.72348	106.43072	133.19129	99.28529	127.28158
Zimbabwe	0.15394747	0.85578198	3.44146899	9.9431005	20.3706685	37.7504192	54.755552	70.381047	86.295505	98.936873	119.759136	144.104742	154.04694	166.04699	177.31658	136.91766	154.00940

Supplementary Table S8. Age distribution of DALYs rate (per 100,000) for Cervical cancer in different countries in 2019.

country	<5	5 to 9	10 to 14	15 to 19	20 to 24	25 to 29	30 to 34	35 to 39	40 to 44	45 to 49	50 to 54	55 to 59	60 to 64	65 to 69	70 to 74	75 to 79	80 to 84	85 to 89	90 to 94	95+
Afghanistan	0	0	0	7.0351344	23.037943	66.860853	156.52328	311.91908	471.21532	558.38954	634.09856	793.4607	639.3883	640.4550	617.8360	528.6846	465.0642	324.4622	223.3139	183.04329
Albania	0	0	0	4.2586478	24.732148	48.838898	94.99253	138.29229	181.23622	221.38134	250.62512	224.6498	238.2593	235.2774	267.3232	266.9758	259.2944	178.5570	134.3477	140.61599
Algeria	0	0	0	2.4696572	10.873968	36.782150	95.58215	180.76467	274.98285	310.83268	329.26301	372.5661	338.6336	338.0426	319.8741	328.5979	333.8855	547.6942	482.1448	208.17680
American Samoa	0	0	0	6.0684456	19.298208	70.764363	192.41115	378.26230	664.93599	647.97158	756.15697	840.5551	604.4876	766.4052	844.1672	664.2909	569.0968	909.1714	831.2461	799.45342
Andorra	0	0	0	1.7664078	9.303120	39.336794	96.33478	160.18706	223.42897	259.89398	307.32142	336.7394	313.0576	342.5059	358.3476	355.1205	339.2049	319.9858	296.4028	320.15524
Angola	0	0	0	13.3363289	53.012116	177.625286	453.21119	741.94078	1160.25510	1416.72008	1780.26968	1949.8499	1775.0149	1699.2615	1567.2847	1333.7784	1166.5035	970.6027	743.1218	719.52029
Antigua and Barbuda	0	0	0	9.4719769	27.660243	75.696345	168.59030	330.77324	485.59547	651.73343	766.24602	940.5173	961.2692	986.1581	1039.4132	975.4349	894.9199	726.4129	672.2062	667.15509
Argentina	0	0	0	14.8825786	58.345055	185.589743	430.36188	636.08377	778.27929	835.04552	929.66196	909.1116	833.3639	793.4296	719.6623	652.5363	609.8433	563.4654	495.8062	415.86389
Armenia	0	0	0	6.3219325	14.372884	43.981558	137.18783	261.15768	471.82858	591.28114	592.67918	650.5762	596.4288	619.4784	635.3644	521.5156	422.7086	262.3339	269.9645	303.93928
Australia	0	0	0	1.1041546	5.037476	27.893727	60.28114	93.42583	114.86284	123.16845	135.45535	159.0455	158.4500	193.1742	207.6678	208.0157	219.1208	168.9436	138.5215	133.97879
Austria	0	0	0	1.0327686	3.306201	19.190016	47.88946	85.95419	127.77113	170.03627	183.31734	232.2816	224.1626	232.1754	240.8343	229.4876	239.9807	215.6181	200.2877	195.06853
Azerbaijan	0	0	0	17.0337469	29.400037	57.148931	134.47261	223.96520	353.55743	424.57256	525.02796	566.3195	511.8427	520.5604	497.8909	434.0534	374.6492	290.0762	307.0294	260.43203
Bahamas	0	0	0	13.2872725	52.747454	156.186184	349.21265	611.40240	638.81560	825.31496	922.23764	920.6314	851.4512	878.6250	793.8637	727.2406	710.1610	521.1660	436.0725	384.86819
Bahrain	0	0	0	8.1608249	15.161799	14.163757	28.73561	55.54063	86.32162	104.70771	122.80549	257.9913	237.7842	280.5693	359.1494	334.3136	359.9118	297.5939	225.9874	219.80016
Bangladesh	0	0	0	4.8336601	22.173581	52.739551	102.14741	198.47502	349.53818	417.95114	635.70569	584.9222	502.6563	449.9257	414.5115	307.2563	287.8321	289.7247	233.0268	256.72746
Barbados	0	0	0	16.5544660	58.931672	135.721819	288.71481	530.55027	692.32508	806.03049	925.96752	981.9719	1146.7613	1097.0800	1152.4300	1127.3270	1058.2963	941.9052	878.6892	836.83126
Belarus	0	0	0	5.8901077	17.692193	66.432768	164.56796	318.11902	421.25496	458.96843	492.38878	562.0079	492.2194	479.1633	452.5365	373.6439	307.9129	243.5682	214.9692	231.76328
Belgium	0	0	0	0.8095036	3.405064	16.274180	45.68491	93.18428	139.23953	197.62950	203.37253	213.5073	213.2959	220.3359	229.0756	230.3769	234.8613	242.8731	222.5322	196.08351
Belize	0	0	0	21.3223313	74.797693	261.088464	618.12159	779.84328	1023.24036	1487.38349	1581.22641	1502.1230	1408.0878	1269.7163	1329.8982	1292.5525	1052.1195	812.1249	733.0296	744.47760
Benin	0	0	0	7.9713646	58.917906	215.880680	380.62888	640.69508	1110.58610	1281.22730	1609.09165	1922.1192	1980.7940	1761.3691	1784.0295	1564.6524	1194.6597	1091.8958	714.2345	736.00830
Bermuda	0	0	0	3.2136813	12.086267	31.813657	66.83925	120.54111	166.80307	208.15979	228.44218	238.5594	231.7592	247.9109	279.6906	294.7745	310.0191	301.0130	300.8478	338.94427
Bhutan	0	0	0	3.2591570	19.366981	51.368282	129.97217	249.20954	443.48309	563.52525	626.76717	691.7036	626.0005	581.2625	614.2730	521.5086	440.8350	438.1650	272.8910	262.51604
Bolivia (Plurinational State of)	0	0	0	11.7643327	37.866983	111.325898	309.70918	636.41695	1100.39293	1511.53737	1854.65280	2085.2945	2048.9641	2276.6533	2386.3247	2276.4028	2033.2464	1727.3833	1088.0966	892.40784

Supplementary Table S8. Age distribution of DALYs rate (per 100,000) for Cervical cancer in different countries in 2019.

Bosnia and Herzegovina	0	0	0	3.1625234	11.280522	39.443902	116.66418	245.81481	339.18790	539.89626	531.31295	471.6912	454.4163	431.2062	453.0456	434.7024	335.5620	248.3689	183.0448	180.09445
Botswana	0	0	0	11.8353397	47.048850	174.024000	453.13548	947.76574	1575.41614	1997.21033	2298.69947	2442.4831	2212.2536	2258.9754	2314.5177	2073.7785	1829.5824	1569.6345	1077.1540	1008.47703
Brazil	0	0	0	9.6796542	36.263743	116.166673	261.40778	399.74399	523.53202	606.85440	659.96129	697.6115	651.0315	677.8674	669.6873	637.5342	569.1882	528.7642	440.8650	457.16660
Brunei Darussalam	0	0	0	6.4440698	18.548115	68.444042	194.37782	368.94315	500.79092	589.42425	800.12920	882.0306	921.5810	1055.2466	1024.6815	857.8008	708.0942	865.3687	606.3031	461.37907
Bulgaria	0	0	0	5.9793670	30.028440	101.109138	288.83637	474.02634	692.82020	828.61075	880.52431	813.8302	651.8982	564.5489	462.3327	370.5038	319.5522	253.2093	181.7465	178.97319
Burkina Faso	0	0	0	8.6904111	67.304228	259.430124	479.08762	774.40101	1357.28511	1506.16876	1796.10204	2132.0498	2234.3158	1954.5891	1982.7288	1762.5776	1308.8636	1137.9342	730.6457	722.43264
Burundi	0	0	0	8.8316689	68.856547	215.518812	492.90605	964.81320	1665.09184	2119.09893	2539.06634	2486.5403	2500.0064	2193.7529	1948.0958	1148.9841	1207.1681	1048.3257	579.3848	557.72291
Côte d'Ivoire	0	0	0	7.3615739	56.228934	202.829231	371.01514	614.71607	1075.35097	1189.15805	1424.91896	1735.5883	1836.9534	1663.7638	1681.2360	1538.7618	1202.4846	1090.5939	686.7361	698.08949
Cabo Verde	0	0	0	8.8273767	38.917295	104.761463	165.86863	291.26547	582.94769	683.31749	776.07383	1099.5239	1205.3758	1184.1522	1408.5497	1381.3622	1290.5568	1612.6772	1122.1697	1052.60224
Cambodia	0	0	0	11.9157419	28.992565	67.832646	166.67615	329.40448	600.68131	742.92435	968.15845	1011.6562	871.3163	821.0821	786.1618	630.7462	494.1903	417.4810	270.4887	199.44329
Cameroon	0	0	0	7.5984440	60.153590	221.981353	416.90610	710.88496	1229.25245	1364.23411	1600.83367	1931.3482	2036.7982	1807.1852	1849.7728	1621.3114	1268.9950	1142.4690	726.6712	710.06381
Canada	0	0	0	1.1027952	9.291942	37.587490	79.49618	112.59310	147.38299	165.94716	175.47815	191.8125	186.1864	200.7832	212.5052	207.1442	212.6435	163.6157	165.5914	197.51060
Central African Republic	0	0	0	18.0097617	67.737153	256.136401	649.01949	1183.06642	1876.18613	2537.12568	2921.27488	3181.5020	2630.8674	2368.5632	2036.0789	1653.5442	1305.4410	1066.4905	757.8474	605.14374
Chad	0	0	0	7.9648004	61.794411	228.669316	433.46058	725.49526	1297.29191	1652.13590	2157.59825	2584.6989	2613.7743	2261.6037	2244.1270	1814.9516	1353.4142	1152.1760	722.6035	690.61785
Chile	0	0	0	4.7142620	18.470575	82.989560	206.09369	314.92300	388.45296	467.10667	481.29949	505.7859	498.0284	590.5473	595.4649	632.1093	625.0945	647.1977	630.8464	504.79962
China	0	0	0	4.8707860	14.175667	35.855417	91.09027	170.43451	281.63668	362.29181	470.22756	506.3274	464.0106	456.2371	443.2740	386.0889	310.6499	249.6005	185.9294	172.85860
Colombia	0	0	0	6.7106797	28.596405	110.570184	253.87764	412.81171	524.42437	569.56784	663.44024	679.8614	687.5883	663.5932	695.3177	640.7335	555.3535	507.6602	385.2476	350.27427
Comoros	0	0	0	6.3547402	65.200408	198.397824	421.30396	846.59675	1433.08542	1792.66576	2094.02421	2080.8002	2178.1345	1955.6446	1788.0998	1087.0916	1139.1863	1027.9901	556.7892	566.68986
Congo	0	0	0	11.1405058	48.355625	182.466086	482.14455	926.86325	1478.32675	1896.98637	2217.96153	2422.0804	2099.0176	1975.1866	1763.6457	1524.3459	1252.0095	1164.5325	907.6726	804.68755
Costa Rica	0	0	0	3.5442799	20.083390	88.561053	201.59096	382.00285	460.61518	494.90419	527.48780	501.3310	524.8390	556.2263	565.9614	560.5957	572.0298	576.5890	478.1405	516.75798
Croatia	0	0	0	0.9650388	4.882778	27.227967	65.39610	113.61019	179.90077	250.27726	305.23073	317.4814	314.9883	336.8643	309.2156	304.9830	317.3205	293.0650	273.5070	265.29915
Cuba	0	0	0	5.1388478	26.920282	97.891673	213.23697	350.30090	490.26648	552.01587	617.33375	646.6111	628.7298	639.4124	619.9436	571.1901	494.0558	365.6907	333.8286	269.55594
Cyprus	0	0	0	1.5676050	2.946808	12.507096	28.16935	49.78113	137.05576	115.70297	189.83172	191.7585	192.5041	250.2665	249.9416	327.7393	365.3481	370.7253	337.9614	399.51813
Czechia	0	0	0	1.6320484	10.367230	36.880851	111.70618	165.15739	248.02360	299.17548	327.41154	377.9921	374.5064	394.3469	384.8159	322.1680	300.6105	300.5553	219.5354	166.55638
Democratic People's Republic of Korea	0	0	0	10.3358287	29.154087	86.128550	190.84171	337.69632	535.45909	783.35633	887.36082	935.9985	764.3455	663.0065	536.1920	432.8138	314.8217	236.9029	147.9355	134.85763

Supplementary Table S8. Age distribution of DALYs rate (per 100,000) for Cervical cancer in different countries in 2019.

Democratic Republic of the Congo	0	0	0	12.7634019	50.706313	171.179844	451.20954	797.97913	1188.73723	1611.06310	2066.52480	2307.5165	2049.8429	1897.1054	1702.1707	1374.7301	1150.3712	961.1900	741.1155	708.89989
Denmark	0	0	0	1.1010399	10.237656	32.090532	73.10406	108.54560	169.73180	181.68325	226.01268	256.5809	258.5795	254.1887	302.9613	327.5913	329.9209	277.3967	222.4676	216.33874
Djibouti	0	0	0	4.7860780	40.360605	134.517729	340.67513	692.93282	1276.44366	1754.69793	2075.95831	2085.7972	2166.6665	1993.8758	1812.3578	1155.7984	1248.6912	1097.2911	600.1324	593.58338
Dominica	0	0	0	26.0025579	98.264003	241.875288	529.67887	803.17211	1080.21989	1366.92547	992.76671	1113.0481	1384.1271	1442.1025	1406.3764	1481.8169	1371.8996	1077.4202	1011.2892	951.94993
Dominican Republic	0	0	0	17.2991648	57.086057	154.144149	331.35648	641.98100	774.27811	882.41518	1072.89164	988.8057	1059.8671	931.1097	1113.8140	883.5914	998.6861	1189.6170	958.2342	892.88798
Ecuador	0	0	0	10.4339267	28.915169	77.661464	254.27016	503.07558	720.63693	862.59148	993.03748	1035.1719	1045.6081	1073.7188	1070.7251	1090.8739	1072.0420	1249.6749	1218.1317	1327.61600
Egypt	0	0	0	1.7738740	3.420197	7.997700	19.04798	36.47652	52.38329	66.52509	92.56099	138.7539	151.5804	191.6123	203.0258	202.9312	194.3478	162.6341	127.5542	106.86628
El Salvador	0	0	0	9.9735871	34.457451	104.572678	326.30250	594.05282	872.77825	1092.26742	1282.82417	1389.6470	1241.3746	1209.7854	1225.0292	1198.5381	1107.1144	1034.3331	738.5941	621.38891
Equatorial Guinea	0	0	0	8.4131074	35.084867	128.568131	333.10887	581.04173	885.33899	1173.39933	1378.67882	1556.9692	1444.6628	1499.6496	1464.9285	1364.8453	1241.6126	1111.9175	831.4718	762.37476
Eritrea	0	0	0	6.3086539	54.573464	186.715238	457.85374	1080.85024	1997.42448	2765.48493	3146.07904	3082.3557	2944.6405	2542.3720	2288.0621	1348.3910	1382.2134	1307.0742	720.0097	644.33093
Estonia	0	0	0	4.7288941	13.467850	44.442960	109.08399	199.79471	289.27068	403.06137	444.22904	478.9871	490.9021	407.8580	416.8710	374.8248	326.6673	257.9390	227.4431	244.77117
Eswatini	0	0	0	13.1655524	46.872464	154.791384	416.93765	854.15336	1483.77391	1978.06184	2296.05948	2390.5177	2137.0019	2288.8850	2339.7537	2187.2495	1999.1435	1773.3116	1232.6211	1144.69591
Ethiopia	0	0	0	4.3335907	36.391696	113.461296	250.03721	471.82033	759.38455	1112.91033	1470.57187	1547.2376	1726.6876	1644.2655	1671.1972	1145.3257	1085.3642	885.7541	458.4821	448.67144
Fiji	0	0	0	20.1680092	50.947841	182.942460	472.35688	878.07763	1499.67772	1545.60041	1801.65365	2112.0743	1632.0294	1869.2606	1877.2600	1353.9000	1075.5890	1808.9840	1540.9029	1394.54578
Finland	0	0	0	0.8025541	3.527842	14.983111	30.24502	45.46140	64.19385	81.22163	95.49800	128.5003	136.1433	183.2994	195.0972	205.2288	208.0152	162.2895	141.3639	122.86648
France	0	0	0	1.0679994	4.201069	18.411491	55.96028	100.93531	162.25271	204.48091	228.54303	230.0381	198.3538	213.7254	222.1995	221.3562	231.2588	230.1109	217.7740	207.32726
Gabon	0	0	0	8.6395397	32.962688	114.211247	310.37899	568.78121	894.93102	1187.17962	1402.69697	1547.3308	1421.4528	1453.8591	1384.8843	1280.7799	1152.4235	1078.4184	854.5004	800.24879
Gambia	0	0	0	3.9271496	31.025621	134.115278	303.32364	591.66526	1058.47603	1256.39676	1573.48022	1704.4289	1609.0721	1297.4682	1336.5912	1126.2471	857.8468	837.5661	457.7074	403.06764
Germany	0	0	0	1.0664868	5.061182	25.332771	64.88551	113.96126	167.63193	194.31886	222.34401	245.4612	226.6116	219.4609	232.4266	239.9845	250.3930	243.2793	213.0106	200.15060
Ghana	0	0	0	6.6241929	55.060885	199.328264	369.78300	595.90248	1061.83166	1091.34053	1267.05230	1490.8587	1571.3679	1454.6741	1484.3802	1329.6649	1071.0705	1020.6278	623.4896	609.72224
Greece	0	0	0	1.9184681	6.690017	25.149675	61.30504	91.25061	153.37503	199.85136	241.44530	249.8568	216.8302	225.9135	238.0062	243.0679	261.3786	275.8311	286.7633	260.19880
Greenland	0	0	0	4.3812398	25.219827	138.885042	289.17287	471.66617	603.27345	720.40294	743.43473	804.5471	728.5195	719.5262	669.6337	599.3953	570.6150	498.0859	362.5234	269.64696
Grenada	0	0	0	18.5133386	66.659519	183.573020	474.85735	853.69210	929.20886	1032.28783	1239.95321	1414.5816	1490.7278	1515.0879	1489.6807	1271.3263	1147.3419	958.0275	828.4472	782.16256
Guam	0	0	0	6.1547281	12.585859	41.828968	122.17722	278.34998	462.24629	452.69642	541.69110	510.9623	407.5163	490.7039	406.3324	406.5894	310.4789	478.3335	374.1860	329.22272
Guatemala	0	0	0	21.7963596	50.716458	137.826404	360.14913	667.02347	1017.55904	1349.31512	1587.19567	1720.2337	1588.3031	1541.4633	1452.0819	1184.5291	1126.9086	1356.1231	1224.2085	949.24246
Guinea	0	0	0	11.0651735	85.554351	335.350510	703.66957	1360.71928	2434.60653	2791.52086	3343.67037	3365.8445	3261.1364	3067.5554	3005.3811	2456.8666	1812.9281	1570.6973	938.8937	917.99357

Supplementary Table S8. Age distribution of DALYs rate (per 100,000) for Cervical cancer in different countries in 2019.

Guinea-Bissau	0	0	0	9.1223333	74.860451	313.420148	621.64446	1144.13029	2061.13734	2253.54142	2467.30913	2959.5069	2841.2949	2356.5201	2292.8263	1875.7625	1366.4120	1298.4835	800.9080	691.04959
Guyana	0	0	0	24.4564952	106.101877	299.898747	625.04474	1042.32076	1243.63352	1592.48632	1763.82473	1951.1795	1816.3823	1687.9544	1671.6099	1364.7857	1112.6598	810.5079	855.6935	915.16413
Haiti	0	0	0	35.3988671	136.574531	398.072979	811.99329	1399.11161	1918.34952	2166.06665	2363.27119	2494.1380	2294.8275	2167.8587	2136.7182	1785.0472	1526.9764	1046.9766	821.3322	694.03564
Honduras	0	0	0	8.5273359	25.918114	79.717082	221.37912	421.52958	695.24691	828.17102	969.52989	1052.8712	938.5336	923.0508	906.7365	767.0079	687.3797	585.3120	552.8426	651.76573
Hungary	0	0	0	1.4122828	10.817602	43.736024	123.37380	245.58975	317.14597	473.21610	495.83224	525.4268	454.7454	416.0686	344.6424	299.1756	292.2684	256.6572	189.1702	145.43171
Iceland	0	0	0	0.8083136	4.404004	17.309108	41.38575	72.14930	97.44622	112.54038	130.74405	140.7237	138.4186	160.1054	177.3673	188.7125	192.3050	158.3494	133.6983	149.39836
India	0	0	0	6.3643438	36.176673	75.054289	163.17660	284.19151	482.67977	558.40360	789.55871	749.5163	673.1079	574.5345	532.1145	401.8742	362.2669	312.5934	247.2835	262.07934
Indonesia	0	0	0	8.7565542	19.872644	46.761873	114.06357	225.83295	414.84954	517.78393	652.83242	699.9687	613.8004	600.3810	609.6086	517.8580	429.9944	392.9330	270.4934	208.74206
Iran (Islamic Republic of)	0	0	0	4.2433168	6.518569	14.438979	28.16005	48.15841	70.31821	87.97221	112.29222	161.5964	164.6157	201.3111	221.9311	236.0085	235.8488	176.8044	137.2019	148.43519
Iraq	0	0	0	2.6383390	6.944458	14.693700	30.94347	68.96255	113.79265	160.79123	210.95102	241.0242	178.9060	206.4831	219.2353	208.7885	209.6284	143.4894	107.3071	91.33766
Ireland	0	0	0	1.0831378	6.080539	33.112036	76.45710	139.44102	193.34738	183.81822	239.55425	263.0117	220.8300	225.3671	223.8672	203.6529	192.4219	177.4177	134.5261	128.90877
Israel	0	0	0	1.3589013	4.070443	19.244399	45.93550	102.44883	124.24899	143.41741	169.94597	208.3032	198.6517	215.9590	232.1959	240.9608	226.0037	196.0726	197.7165	234.19872
Italy	0	0	0	1.5016475	4.407492	15.938889	40.54865	74.24173	110.38156	135.11128	168.12137	190.0529	173.3185	197.5618	211.0299	209.7008	215.2371	184.5736	150.1439	166.39808
Jamaica	0	0	0	12.0404039	54.922044	160.233013	427.56573	738.70303	1033.49208	1303.23296	1251.06087	1340.2190	1462.8991	1433.5366	1221.5584	1052.9841	903.1691	678.8702	538.7059	631.56417
Japan	0	0	0	1.0073881	4.558915	25.188262	77.55501	139.24478	190.98625	238.89324	271.61692	236.7684	196.3721	195.6499	204.1970	201.6857	212.2581	177.6874	157.5988	146.27443
Jordan	0	0	0	1.3628645	3.828311	9.906981	25.66786	55.76457	79.93772	99.68062	132.99364	164.0476	179.8198	200.0287	205.5574	210.2808	200.1930	163.2344	131.8622	141.03915
Kazakhstan	0	0	0	9.0314059	22.057548	84.588428	241.27887	442.74610	647.52837	699.28802	698.82476	651.2009	595.5375	574.2697	519.3972	433.6406	369.1967	246.2906	234.2421	299.13639
Kenya	0	0	0	2.1412148	22.506549	72.258391	179.33642	388.57729	725.56937	1023.81380	1250.70636	1238.1819	1252.5445	1159.2243	1124.7639	717.9634	762.1699	720.5975	375.7839	353.15513
Kuwait	0	0	0	2.2811512	2.570717	6.567885	16.86560	31.60899	48.57763	70.89595	83.44750	146.9522	133.3770	206.8153	201.8699	206.6430	213.5411	149.9798	110.7700	123.25945
Kyrgyzstan	0	0	0	8.8833727	19.347022	54.858210	193.53453	383.71980	612.96723	825.10301	866.80210	812.0809	804.3005	773.5644	657.9994	551.6708	449.8186	270.1591	249.3588	240.90517
Lao People's Democratic Republic	0	0	0	12.7144164	30.084888	73.364224	184.67272	342.63412	613.59534	704.04994	885.54324	928.2474	796.5886	725.4295	696.8273	550.2512	442.4088	360.5281	233.0391	172.97307
Latvia	0	0	0	2.1451689	8.763604	33.433991	91.23237	147.45074	241.29025	309.70830	371.70563	405.6594	396.2898	411.9083	396.7092	333.2817	309.5673	216.6298	211.6200	238.75234
Lebanon	0	0	0	1.4036489	5.996201	20.110155	47.60928	86.77215	130.52557	145.28339	173.98584	216.6107	201.5216	222.8087	229.4082	207.5088	202.8960	166.5450	125.5230	123.70962
Lesotho	0	0	0	14.6093400	53.890082	187.048740	519.38088	1123.35331	1990.10485	2816.79564	3308.79126	3514.5144	3115.7293	3078.7084	3017.8170	2679.8943	2305.1432	1999.3253	1369.6916	1209.76517
Liberia	0	0	0	7.1992063	61.259447	212.234519	377.25058	649.48658	1117.66585	1338.06592	1654.86920	2010.6209	2022.7615	1781.6175	1775.9044	1552.0901	1232.3982	1130.6731	712.6285	689.56102
Libya	0	0	0	1.6652376	7.995906	27.646584	75.68682	139.92984	225.93489	289.96810	327.76927	392.2532	394.1846	407.7132	336.2629	292.9250	222.5882	176.1757	136.0852	135.27032

Supplementary Table S8. Age distribution of DALYs rate (per 100,000) for Cervical cancer in different countries in 2019.

Lithuania	0	0	0	2.6590372	8.193072	44.840559	130.21201	211.02715	293.73799	373.24231	439.98592	497.2848	513.4421	519.1617	423.8020	386.5699	355.6881	297.7976	239.5686	198.61219
Luxembourg	0	0	0	0.9746248	4.167190	16.768080	42.70257	70.45566	93.17643	113.03636	127.91885	144.1268	144.2158	169.0563	185.4939	189.1886	190.3280	160.2466	147.6638	153.45508
Madagascar	0	0	0	6.7809837	60.875387	206.428492	473.47798	909.20239	1515.81889	1895.61910	2204.18105	2166.8790	2126.1539	1795.5819	1610.9660	935.7290	959.5478	870.5587	479.6505	458.59971
Malawi	0	0	0	8.7019624	74.231418	254.645275	592.85978	1049.07388	1614.54839	2012.36416	2420.48562	2371.8119	2460.2673	2153.4046	2037.0830	1312.4710	1382.8048	1212.1858	673.0399	693.85859
Malaysia	0	0	0	3.9048317	13.306136	35.904787	99.97930	195.91091	348.03641	484.40319	651.10886	774.1077	777.8290	825.3498	949.8582	894.7079	866.5124	806.2334	487.0234	390.73788
Maldives	0	0	0	3.5946446	7.277929	16.552879	46.16134	94.04956	167.99658	216.07610	284.36426	328.7200	334.5470	365.2571	423.1130	426.0734	439.8050	420.9058	282.5046	222.53137
Mali	0	0	0	5.0466157	36.634658	151.369679	332.45388	636.08538	1094.88596	1393.13714	1714.97717	1912.0712	1895.3119	1593.5813	1529.3369	1204.6033	882.3275	676.4924	350.1139	329.42810
Malta	0	0	0	1.2139611	4.090742	16.175720	39.40174	61.96969	84.93760	95.97577	110.73220	136.3811	138.0664	170.6059	183.8687	178.2943	172.4140	132.7221	102.6259	102.32054
Marshall Islands	0	0	0	13.4585813	50.943266	205.767521	514.10699	1037.91854	1789.37729	1727.16859	1914.95227	2072.4338	1353.2485	1617.1031	1625.1911	1225.0202	986.3016	1410.6184	1119.1648	1016.97755
Mauritania	0	0	0	4.8194005	39.309382	138.877528	267.72379	453.06275	860.44588	1061.04382	1403.74012	1726.8315	1845.5616	1673.5302	1712.6913	1509.3441	1204.1703	1148.3890	705.1843	674.48770
Mauritius	0	0	0	3.3388656	8.316518	26.983536	81.28302	143.02405	311.41323	307.47718	393.01082	502.4095	534.3857	528.9204	619.1367	528.0230	507.8778	380.5723	316.9662	157.73035
Mexico	0	0	0	5.4717244	21.162959	84.466159	211.77715	356.11036	525.34073	635.66038	735.73426	785.5703	776.3210	804.4948	774.8030	762.0538	724.2627	725.7085	625.2423	591.11884
Micronesia (Federated States of)	0	0	0	11.7600418	49.675071	196.043461	477.63214	805.61424	1207.59413	1462.74767	1834.34779	2016.1956	1353.7542	1590.2346	1651.3426	1258.1710	1041.4929	1494.4994	1207.3581	1081.70951
Mongolia	0	0	0	2.6187116	9.122714	61.204811	211.38191	424.75694	730.29692	930.94071	1026.14139	973.9924	1066.0826	1078.8850	942.3255	874.7893	882.9718	779.0094	867.2511	963.60129
Montenegro	0	0	0	2.4153362	19.611623	60.768243	154.70278	241.20787	341.86368	385.52406	454.37228	445.1845	385.2297	380.1425	349.5153	299.3332	281.3418	222.9115	161.9172	138.21919
Morocco	0	0	0	3.0794495	11.472619	27.972514	70.27466	184.00108	357.99907	593.25054	796.68577	969.6493	744.2591	717.7138	631.2756	525.2378	453.2340	344.3639	240.8888	213.74259
Mozambique	0	0	0	9.0515260	71.912657	252.980887	580.76086	1101.49964	1806.75623	2380.98057	2757.72409	2701.8655	2737.4875	2399.1947	2254.0165	1476.6024	1572.5719	1485.1537	804.8718	743.94489
Myanmar	0	0	0	13.3874896	30.347435	67.641108	168.08968	305.55471	516.73228	591.30951	723.04836	752.9576	659.1015	618.5294	627.4118	520.2011	425.5759	344.9687	225.3229	172.10984
Namibia	0	0	0	5.2867736	28.639020	97.053939	257.53071	512.95099	860.14094	1127.93461	1388.11035	1426.3679	1477.3930	1513.0308	1593.0370	1404.5752	1071.6544	896.7320	504.0967	448.78752
Nepal	0	0	0	4.1450513	23.830848	62.842815	156.84001	301.71006	542.76734	694.67328	793.79073	769.5977	720.6989	627.5636	630.7601	516.9467	404.0433	412.7130	299.2714	282.27835
Netherlands	0	0	0	0.9059393	4.538050	23.813305	63.49620	89.99094	114.83654	130.66482	153.95365	157.1506	151.1947	188.7793	206.8655	207.6242	218.2344	211.5356	214.5210	174.36211
New Zealand	0	0	0	1.1512966	5.775479	33.898433	64.89538	97.28447	128.34363	156.72515	155.96370	179.7924	173.5717	202.0287	214.7413	211.7058	214.1628	170.1000	128.4730	120.89871
Nicaragua	0	0	0	6.6160136	25.895450	83.962509	238.73033	487.00728	782.28415	1063.68506	1229.03019	1257.7477	1348.4503	1473.3272	1523.4609	1605.6184	1543.5870	1504.7675	1073.0344	976.76376
Niger	0	0	0	8.3397463	60.625205	229.842315	417.71719	698.24293	1219.07556	1478.09262	1971.12788	2344.6015	2413.1133	2128.7577	2155.0100	1822.9912	1343.5063	1171.0983	728.4830	712.37334
Nigeria	0	0	0	2.2055353	22.354826	106.507711	220.98348	380.15731	618.53443	789.64057	965.47894	1090.6337	1168.5438	1106.6357	1103.2829	1007.8864	757.9349	687.8756	399.3061	385.14514
North Macedonia	0	0	0	3.6290863	17.952443	58.078126	121.02963	243.43156	353.46940	493.77170	509.60198	571.3603	515.1483	519.9147	458.8676	513.6065	405.4699	333.1211	229.6816	174.77269

Supplementary Table S8. Age distribution of DALYs rate (per 100,000) for Cervical cancer in different countries in 2019.

Northern Mariana Islands	0	0	0	8.5424946	26.495116	93.648933	269.01077	566.20085	1019.61916	1022.89258	1241.82287	1384.6601	1047.1623	1329.0827	1422.5459	1105.9919	966.9301	1543.8919	1374.1844	1367.13621
Norway	0	0	0	1.1614162	7.797436	24.696209	57.61654	104.92624	135.73875	167.09128	194.61414	206.4390	216.6555	244.7663	251.7955	271.3946	241.3775	262.4755	207.2925	225.06106
Oman	0	0	0	1.4574724	5.109661	14.148340	30.15091	58.77197	86.79561	109.07212	156.37938	247.1547	241.4296	330.5247	396.7962	402.2335	366.6572	247.5963	175.6929	156.25194
Pakistan	0	0	0	2.2038181	17.967422	54.943198	124.47121	210.59908	315.04997	368.07495	416.46824	429.4074	404.6776	356.9334	361.2734	269.5402	213.7289	177.0771	108.5299	106.09690
Palestine	0	0	0	3.2165746	9.321076	15.726779	26.28419	48.63282	58.71716	83.92154	122.74852	209.5493	223.3667	332.6420	389.0490	386.2397	341.4475	236.8152	175.8411	123.77897
Panama	0	0	0	7.5597271	34.673447	152.013558	358.84706	479.32372	673.44324	704.13100	763.45217	824.2951	775.9870	792.1532	772.9626	700.3221	625.0954	656.0567	473.5262	496.12616
Papua New Guinea	0	0	0	11.3950346	33.220925	126.440077	309.50788	623.28034	1114.97782	1034.88966	1253.01042	1367.6523	957.4659	1199.3820	1166.7214	869.3967	673.6806	891.2104	693.0556	613.98694
Paraguay	0	0	0	11.7329668	46.941095	170.577858	441.87870	809.60732	1159.03961	1378.24046	1420.40726	1386.9756	1355.7716	1252.9987	1171.4863	1152.7091	1071.5902	968.1225	676.9932	760.02049
Peru	0	0	0	15.5253290	35.806679	88.817009	222.12729	410.12717	664.19309	858.80884	1001.29703	1086.3551	1000.2142	1054.1852	1056.1238	1040.1355	904.4598	840.0733	640.6409	630.37349
Philippines	0	0	0	11.4736996	30.691881	69.383395	167.31555	291.27216	479.86949	573.56468	686.65443	722.6306	583.3492	524.5033	474.3120	377.8193	305.7135	277.2620	213.3830	168.87809
Poland	0	0	0	1.9383761	8.321978	30.432387	79.55191	174.83542	300.96352	440.24143	529.73992	616.0723	569.9950	537.1225	483.2881	413.5588	384.1975	341.7608	247.4095	192.34893
Portugal	0	0	0	1.8758055	5.243376	19.784606	62.89072	116.92647	183.09488	249.31604	263.15127	272.4415	240.7607	256.1005	269.8410	260.1378	260.0129	237.0117	217.7803	230.64603
Puerto Rico	0	0	0	2.4442190	15.935784	67.572268	141.56227	216.79299	278.37184	286.28009	277.18982	277.2514	267.0146	283.5712	301.2821	278.9582	247.9810	208.4364	187.5512	186.13075
Qatar	0	0	0	2.8434320	5.663684	14.235683	34.77124	61.99434	72.15493	93.46408	133.53917	268.7557	345.8512	456.5119	557.3718	593.6094	1003.0931	1015.6469	537.2028	539.20509
Republic of Korea	0	0	0	1.3146696	4.756673	22.277258	66.18328	113.30408	140.63389	160.54800	192.22532	183.0961	174.5959	207.7438	238.6385	255.2602	296.3945	313.9898	265.5196	236.47000
Republic of Moldova	0	0	0	7.7606322	23.363277	85.676775	198.08568	342.14195	507.59530	604.44496	541.48288	575.2876	547.0932	520.3135	455.4095	355.2770	259.5836	164.5730	125.6852	108.64952
Romania	0	0	0	6.4785287	29.904224	103.269241	255.23853	468.56944	708.26635	922.28257	1149.48576	1171.5349	1041.7346	898.6492	778.7374	652.7970	479.3969	335.7432	193.4593	144.49614
Russian Federation	0	0	0	2.2952731	16.423130	91.340145	243.62782	393.43320	492.87327	509.60231	491.70827	460.1697	417.7892	389.8886	352.8417	300.9026	250.5243	178.7532	151.5862	156.30050
Rwanda	0	0	0	6.6696484	56.096085	174.693599	399.01778	746.64918	1189.42842	1556.22448	1950.31462	1914.7929	1994.0003	1844.8772	1746.5996	1093.5262	1180.7705	1091.4622	611.1314	593.63402
Saint Lucia	0	0	0	12.0175335	48.675063	163.745423	369.65115	628.23728	906.13635	1116.79932	1040.56095	1070.1516	1021.7918	1076.9941	1020.0948	866.8000	853.7003	874.7616	938.8376	960.16874
Saint Vincent and the Grenadines	0	0	0	20.6338451	92.369039	283.148726	585.54605	981.22133	1345.15774	1681.93298	1659.72245	1574.4208	1490.1555	1470.2231	1613.5500	1502.2369	1538.9735	1254.9955	1102.9831	980.32426
Samoa	0	0	0	3.7703973	22.155684	106.028353	282.08946	591.21830	994.03816	1015.82469	1171.98661	1282.8445	932.2654	1029.4754	950.5892	675.4196	486.7703	574.9409	383.7825	331.26261
Sao Tome and Principe	0	0	0	10.1633841	72.628968	252.354019	486.53697	879.70005	1542.64360	1868.48395	2151.50885	2494.5085	2476.0138	2260.0664	2287.5350	1989.5118	1530.3950	1615.3931	1028.1419	974.30954
Saudi Arabia	0	0	0	0.9498418	4.211877	13.423546	38.02562	67.05372	103.39961	123.44424	148.18839	200.0354	182.5112	221.3057	243.8254	242.1909	240.6893	183.8219	130.3637	119.54317
Senegal	0	0	0	6.8517377	50.501495	184.833409	331.09916	556.87080	1056.27753	1230.48760	1564.69469	1926.2211	2005.4160	1819.0029	1845.6398	1612.2149	1281.6939	1190.2178	733.6615	685.85653

Supplementary Table S8. Age distribution of DALYs rate (per 100,000) for Cervical cancer in different countries in 2019.

Serbia	0	0	0	1.8972854	11.837710	58.157808	170.69593	342.01698	544.31310	726.66869	821.32828	851.6845	738.7442	691.9391	580.4749	542.0500	561.8165	452.8114	335.3783	288.99472
Seychelles	0	0	0	18.2890701	43.996322	119.666146	280.04501	529.03047	859.24562	1068.40189	1324.02003	1280.4529	1239.2928	1327.9145	1407.6884	1439.2181	1275.1981	1233.1869	1000.3577	897.99161
Sierra Leone	0	0	0	8.2127857	64.858413	244.090582	444.48908	747.43042	1320.42081	1504.40877	1836.24300	2186.2937	2252.5531	1950.8285	1904.0462	1645.2465	1262.1770	1115.4564	697.9507	700.28934
Singapore	0	0	0	1.6738735	3.216046	9.534756	29.12664	53.24864	95.19591	139.33713	209.46129	239.4539	229.7141	250.9274	253.4181	258.7416	270.3589	216.5369	187.0938	186.09633
Slovakia	0	0	0	1.3632047	10.638982	46.606897	119.87635	253.97992	358.51825	442.66039	472.27137	481.6813	459.4378	420.3065	379.9220	342.5249	315.3842	236.0225	180.5349	173.47103
Slovenia	0	0	0	0.9136241	5.851464	22.598019	58.99458	104.64592	164.20982	209.36185	239.72286	233.5808	233.4694	239.8171	253.6300	250.6702	256.4133	189.8955	175.7560	202.86461
Solomon Islands	0	0	0	23.3925736	96.395119	402.692050	873.18141	1682.88138	2706.91537	2588.62634	2786.73453	3039.1161	1817.6988	2107.5207	2019.8114	1467.7909	1022.7566	1336.0053	968.6206	774.66043
Somalia	0	0	0	8.4342116	72.628752	243.135799	557.72889	1188.02017	2066.16987	2769.89000	3256.96180	3277.7015	3049.7626	2606.6490	2231.9266	1304.8901	1279.7054	1140.5209	603.7614	525.28535
South Africa	0	0	0	5.5691184	29.524693	163.297969	475.27134	703.82219	911.68070	1068.42315	1250.31961	1386.8471	1317.3533	1437.0617	1311.1609	1144.3681	1317.0797	1289.9275	1059.6388	1157.02748
South Sudan	0	0	0	5.2851402	43.200916	143.228909	331.34320	642.71402	1024.18437	1362.19983	1713.21725	1721.1926	1815.4398	1675.0382	1596.8004	979.7019	1011.0036	834.1821	471.7364	478.26618
Spain	0	0	0	1.1211314	3.850484	14.498952	40.61522	75.18220	133.88220	177.71943	193.85928	205.2076	180.7971	201.4673	214.0363	216.7374	224.2566	177.6827	161.7810	152.13997
Sri Lanka	0	0	0	4.1880525	9.254372	16.425937	43.96700	91.85366	163.63407	204.99950	280.89719	301.4578	306.0933	341.3747	388.2416	301.5972	280.2298	290.8088	265.9696	260.06474
Sudan	0	0	0	3.1215200	8.388764	21.221355	50.31018	98.21741	149.00629	184.74230	233.06940	300.4552	281.3540	317.0523	331.9412	304.3736	279.2266	185.7071	119.6612	104.60221
Suriname	0	0	0	27.1973451	74.110729	258.373622	501.49395	973.84094	1078.52724	1199.92190	1235.70465	1417.0244	1361.2736	1229.3041	1403.2269	1276.4266	1056.4208	791.1677	633.4624	580.60972
Sweden	0	0	0	1.1641084	7.402792	29.452019	71.50024	87.39469	124.92067	141.37685	167.26618	190.6911	189.0438	239.9702	243.9013	278.2619	259.3494	261.4220	262.0434	291.80480
Switzerland	0	0	0	1.0722059	3.448316	13.619307	34.80815	57.91155	84.64271	106.00015	127.65667	161.6793	163.8067	219.5061	223.0299	233.0524	232.9327	209.9925	168.6674	172.26614
Syrian Arab Republic	0	0	0	2.6175392	6.698107	13.297483	27.39940	46.37296	61.73395	78.97267	99.33096	132.6989	136.2687	163.4519	177.0318	178.1544	197.9536	214.0319	175.6624	113.07365
Tajikistan	0	0	0	4.2807341	11.537555	35.782353	92.72614	156.04075	228.58979	270.29783	318.84640	379.7627	411.5862	474.1780	503.7572	450.7377	369.8870	201.5983	152.0555	138.37164
Thailand	0	0	0	9.4158304	22.817780	64.464429	149.05808	267.73019	387.40842	438.21191	545.09648	573.0006	556.2090	574.8842	590.3020	544.0652	479.3894	434.1317	362.3241	334.76682
Timor-Leste	0	0	0	10.2826118	23.551611	54.767836	139.78338	264.85471	491.43355	651.97556	901.17472	979.7261	847.7521	793.9680	757.9917	611.9157	493.2172	407.5584	262.7924	193.04873
Togo	0	0	0	7.0490691	56.636651	203.677021	398.50666	641.40224	1179.12843	1297.25798	1587.88797	1872.5651	1960.6103	1770.7902	1757.0096	1550.8464	1226.9886	1115.5882	692.6591	679.60302
Tonga	0	0	0	8.3141397	28.187207	99.653721	304.25455	609.86752	1075.03673	1024.73607	1264.11307	1573.2908	1214.6970	1638.0712	1571.3334	1278.8694	1087.6825	1625.3952	1490.3863	1385.77987
Trinidad and Tobago	0	0	0	10.7860522	46.315062	114.112818	218.85759	441.12653	605.84540	704.81892	825.52000	943.7134	966.1590	1089.6558	1017.5048	1002.1022	958.4893	684.2530	613.2713	655.89322
Tunisia	0	0	0	1.0711229	5.444003	17.857520	48.22145	89.48633	133.07000	153.08216	173.64867	234.2507	217.7667	248.5359	241.8547	214.6846	222.2722	168.1655	118.2062	114.98413
Turkey	0	0	0	1.6798997	4.231554	9.728923	26.54252	53.59769	85.18671	100.63605	129.35519	160.6374	172.7805	214.6469	269.4336	333.3411	387.3711	287.3862	182.5964	137.45722
Turkmenistan	0	0	0	11.8037724	28.594757	82.583118	202.66349	320.57908	604.74404	700.70532	795.42749	698.4216	653.1689	504.4610	454.2135	332.6850	265.2069	135.8200	128.2447	140.70757

Supplementary Table S8. Age distribution of DALYs rate (per 100,000) for Cervical cancer in different countries in 2019.

Uganda	0	0	0	7.5843912	64.793962	210.759966	478.42963	930.97301	1421.05519	1749.58490	2226.44858	2133.1903	2368.5770	2253.9398	2179.6287	1307.2971	1417.4990	1214.9615	674.7590	691.56845
Ukraine	0	0	0	8.2308116	26.343145	81.810016	152.49533	237.54701	319.58667	378.61900	440.51227	479.7928	477.4755	489.9383	440.1978	331.9107	269.6924	179.9542	146.6129	143.22085
United Arab Emirates	0	0	0	1.6006682	9.948785	14.779388	32.45223	81.68757	156.65939	284.39238	412.92444	549.9919	598.0677	937.5052	766.4165	493.0848	583.0581	812.3480	681.6042	645.64640
United Kingdom	0	0	0	1.7586282	11.532191	54.720086	100.89226	141.21204	175.37152	193.05017	186.39774	210.6272	204.1861	228.4667	243.5184	241.0084	238.7544	203.5792	183.8951	208.23268
United Republic of Tanzania	0	0	0	6.9241499	56.036257	179.424571	419.66331	780.87588	1258.88059	1661.51056	2148.85040	2194.5735	2268.7880	2053.3980	1911.1775	1199.4000	1241.1955	1070.9067	598.5548	597.70837
United States of America	0	0	0	1.7577991	9.963748	45.029391	97.01041	146.23905	199.10158	219.74216	246.04093	268.8116	249.3362	243.7635	242.4143	212.5400	221.2687	158.0117	127.0615	109.27651
United States Virgin Islands	0	0	0	5.7104717	17.812841	51.133793	121.68833	255.83237	395.50124	524.24716	592.17764	645.0000	609.2282	646.6855	691.2669	691.1018	678.4545	584.7054	524.9004	498.87757
Uruguay	0	0	0	6.7806159	31.029133	99.028809	358.72525	474.95544	549.15968	615.73610	679.02101	718.1943	600.6196	563.6331	606.8149	580.8318	586.9855	555.1866	499.4829	412.63992
Uzbekistan	0	0	0	11.7871819	24.299661	61.115686	182.42485	349.74471	610.57440	717.20065	836.17777	823.3781	832.7901	628.8763	575.1335	481.6021	484.1019	306.2708	209.0392	218.13099
Vanuatu	0	0	0	11.3314821	36.815473	147.379153	368.60442	774.53175	1353.85286	1331.29223	1507.22229	1647.1753	1104.5476	1348.6195	1353.6205	1029.1241	815.1078	1155.4055	933.5926	879.79760
Venezuela (Bolivarian Republic of)	0	0	0	19.0647089	81.144763	304.867089	590.19260	841.27968	1037.63720	1112.60097	1204.73612	1223.7372	1171.3809	1125.4102	1181.9910	1046.5820	904.9507	683.2132	537.1299	575.63437
Viet Nam	0	0	0	3.6889933	14.761374	41.855026	110.27186	209.68880	384.36550	502.84647	676.52385	815.1770	822.0351	862.9579	913.1870	783.6474	668.9992	607.2612	418.2258	363.25485
Yemen	0	0	0	2.3928116	7.665704	22.517903	55.77783	117.57516	210.59136	260.15741	306.91733	389.1543	353.2569	385.0664	385.3295	332.5098	303.8756	204.6771	135.0718	121.00129
Zambia	0	0	0	7.8920578	74.442826	237.181450	560.14860	1117.92056	1813.66982	2270.05675	2608.16929	2545.4097	2505.4885	2168.6981	1967.8031	1194.5777	1259.4268	1205.2070	696.4558	681.74371
Zimbabwe	0	0	0	11.3564572	58.616679	218.095491	579.80664	1079.80885	1803.83710	2339.23397	2662.21570	2850.4811	2802.8759	2843.8628	2796.9212	2368.3282	1965.9366	1605.9620	963.2828	830.77571

measure	location	Risk	year	val
Deaths	Global	Smoking	1990	1.28 [0.65-2.06]
Deaths	Global	Unsafe sex	1990	8.48 [7.59-10.07]
Deaths	High SDI	Smoking	1990	1.43 [0.68-2.22]
Deaths	Low-middle SDI	Smoking	1990	1.28 [0.64-2.13]
Deaths	Low-middle SDI	Unsafe sex	1990	11.71 [9.73-15.05]
Deaths	Low SDI	Smoking	1990	1.43 [0.67-2.47]
Deaths	Low SDI	Unsafe sex	1990	19.18 [15-23.66]
Deaths	High SDI	Unsafe sex	1990	4.56 [4.22-4.71]
Deaths	Middle SDI	Smoking	1990	1.19 [0.58-1.98]
Deaths	Middle SDI	Unsafe sex	1990	9.32 [8.31-11.54]
Deaths	High-middle SDI	Smoking	1990	1.27 [0.65-2.04]
Deaths	High-middle SDI	Unsafe sex	1990	6.95 [6.5-8.13]
DALYs	Global	Smoking	1990	39.31 [21.03-62.13]
DALYs	Global	Unsafe sex	1990	275.05 [242.75-326.15]
DALYs	Middle SDI	Smoking	1990	33.63 [17.3-55.6]
DALYs	Middle SDI	Unsafe sex	1990	287.82 [255.02-356.33]
DALYs	Low-middle SDI	Smoking	1990	37.08 [18.61-61.43]
DALYs	Low-middle SDI	Unsafe sex	1990	381.9 [315.26-485.47]
DALYs	High SDI	Smoking	1990	46.79 [24.1-70.67]
DALYs	High SDI	Unsafe sex	1990	143.23 [130.3-148.45]
DALYs	High-middle SDI	Smoking	1990	41.44 [22.5-64.4]
DALYs	High-middle SDI	Unsafe sex	1990	215.21 [201.46-252.85]
DALYs	Low SDI	Smoking	1990	43.51 [19.69-76.28]
DALYs	Low SDI	Unsafe sex	1990	630.59 [487.61-777.41]
Deaths	Global	Smoking	2019	0.69 [0.35-1.14]
Deaths	Global	Unsafe sex	2019	6.51 [5.55-7.29]
Deaths	High SDI	Unsafe sex	2019	2.9 [2.6-3.1]
Deaths	Low-middle SDI	Smoking	2019	0.69 [0.34-1.25]

measure	location	Risk	year	val
Deaths	High SDI	Smoking	2019	0.75 [0.34-1.19]
Deaths	Low-middle SDI	Unsafe sex	2019	8.85 [7.62-10.83]
Deaths	High-middle SDI	Smoking	2019	0.8 [0.4-1.29]
Deaths	High-middle SDI	Unsafe sex	2019	4.89 [3.92-5.47]
Deaths	Low SDI	Smoking	2019	0.95 [0.44-1.64]
Deaths	Low SDI	Unsafe sex	2019	15.05 [11.92-18.46]
Deaths	Middle SDI	Smoking	2019	0.57 [0.27-1]
Deaths	Middle SDI	Unsafe sex	2019	6.78 [5.4-7.76]
DALYs	Global	Smoking	2019	20.75 [10.85-33.51]
DALYs	Global	Unsafe sex	2019	210.64 [177.67-234.85]
DALYs	Low-middle SDI	Smoking	2019	19.7 [9.37-35.44]
DALYs	Low-middle SDI	Unsafe sex	2019	285.64 [244.64-342.16]
DALYs	Low SDI	Smoking	2019	27.53 [11.87-48.17]
DALYs	Low SDI	Unsafe sex	2019	477.53 [374.33-591.38]
DALYs	Middle SDI	Smoking	2019	15.65 [7.73-26.95]
DALYs	Middle SDI	Unsafe sex	2019	204.6 [161.92-233.49]
DALYs	High SDI	Smoking	2019	23.88 [12.15-36.84]
DALYs	High SDI	Unsafe sex	2019	89.72 [81.88-95.85]
DALYs	High-middle SDI	Smoking	2019	25.66 [13.93-40.27]
DALYs	High-middle SDI	Unsafe sex	2019	154.69 [124.02-173.51]

Supplementary Figure1. The incident cases (A), age standardized incidence (B), death (C) and DALY (D) rates of cervical cancer from 1990 to 2019.

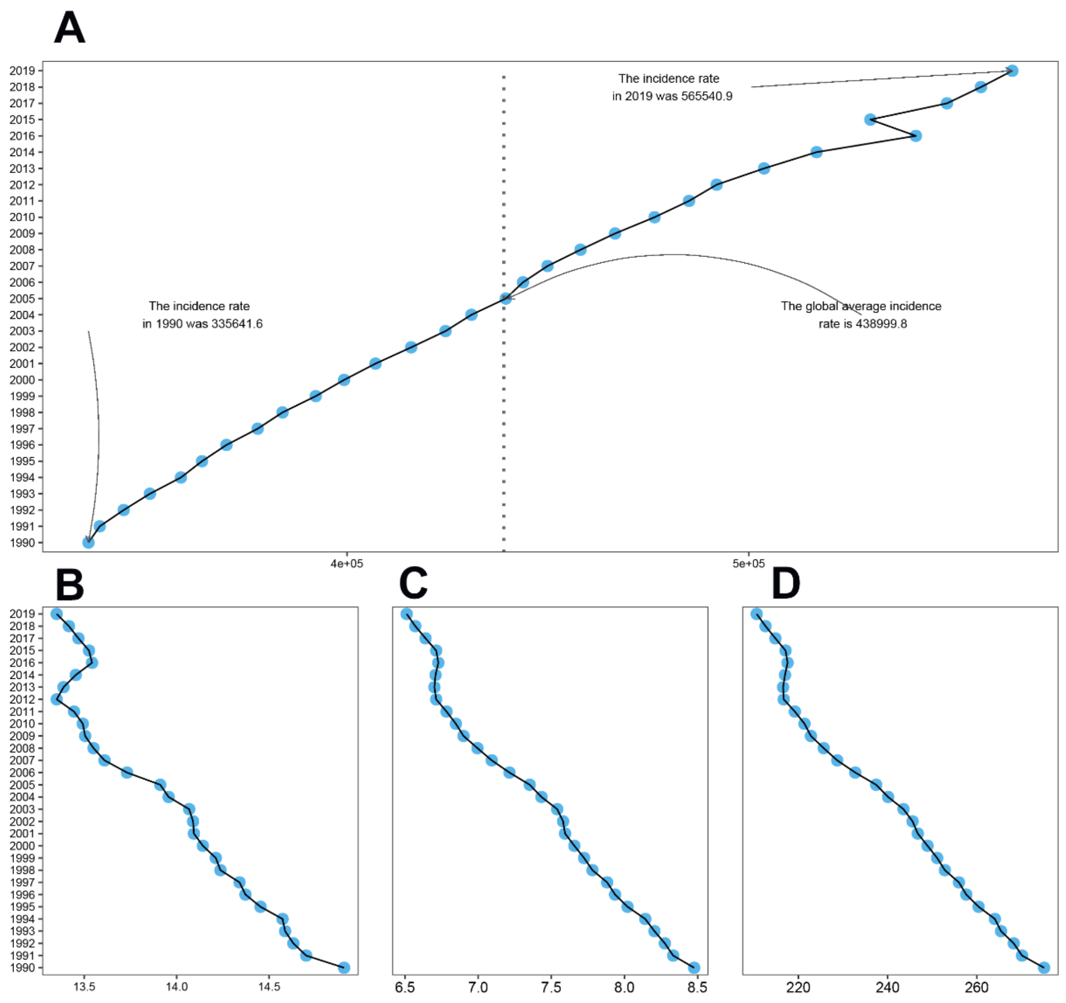

Supplementary Figure2. The EAPC of cervical cancer ASR from 1990 to 2019, by SDI and region. (A) The EAPC of ASIR; (B) The EAPC of ASDR; (C) The EAPC of age-standardized DALY rate;(D) The ratio of incidence among different age groups in 2019.

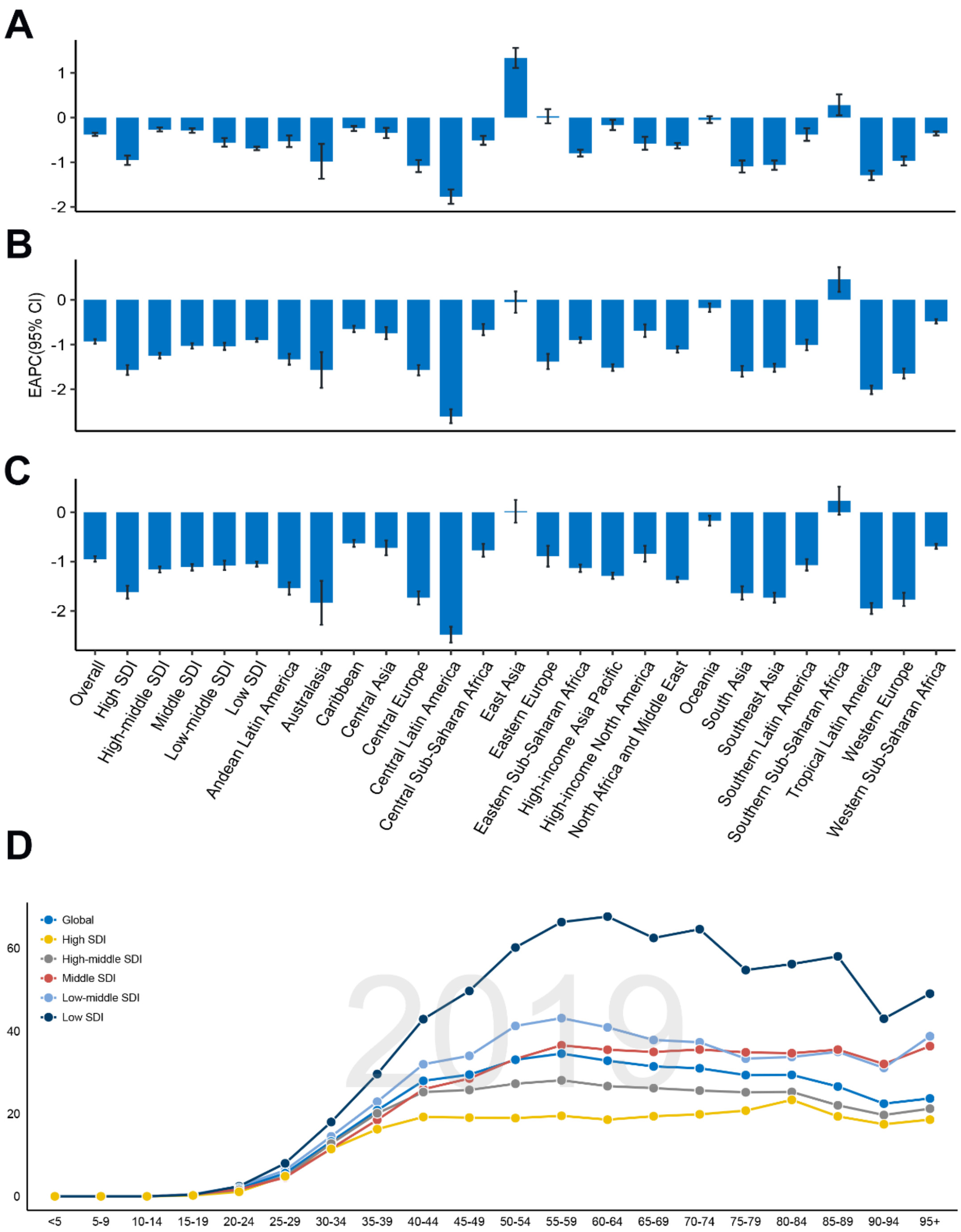

Supplementary Figure3. The age standardized incidence (A), death (B) and DALY (C) rates of cervical cancer per 100,000 population among regions based on SDI in 2019.

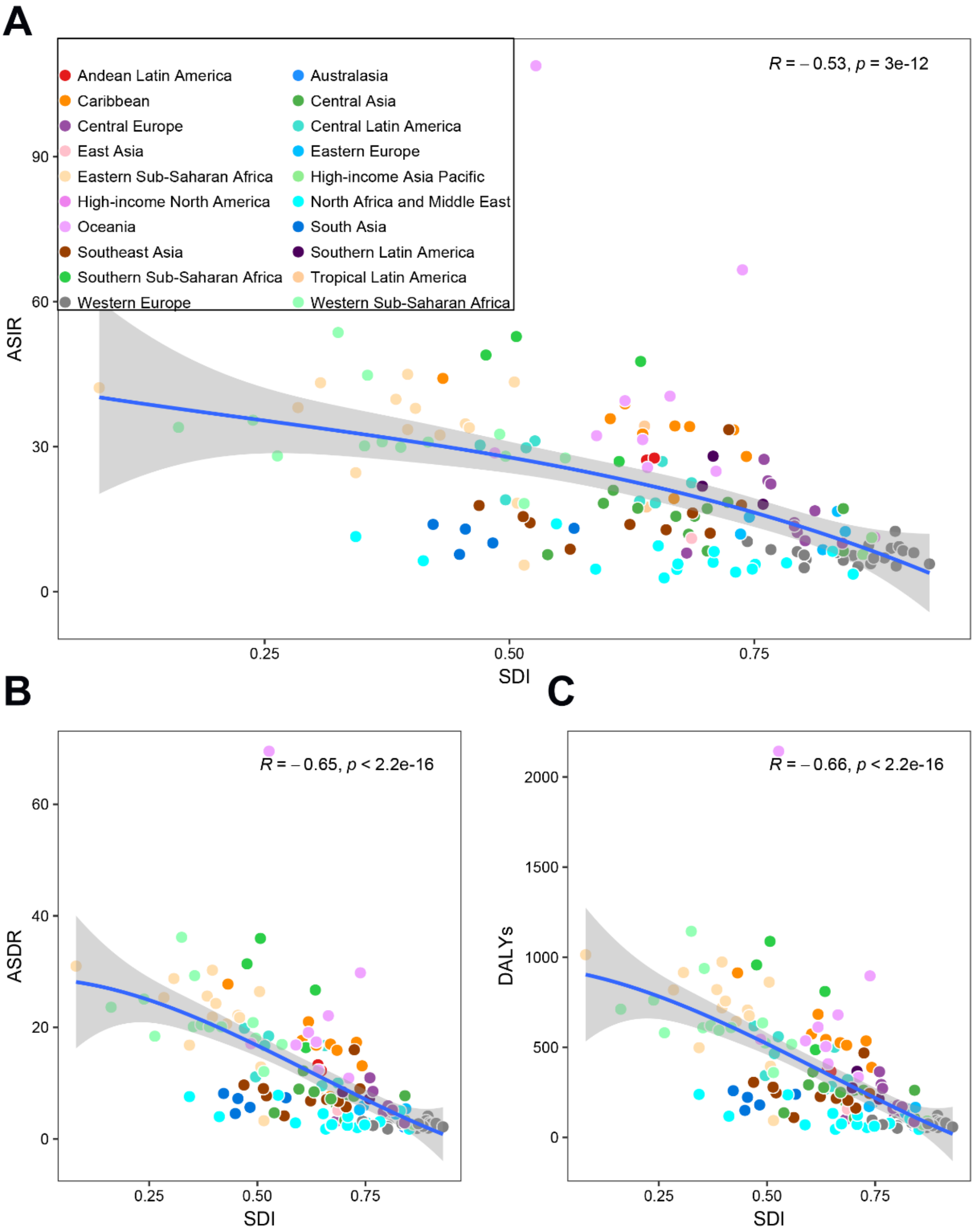

Supplementary Figure4. The proportion of different ages in cervical cancer death (A) and incidence (B) and by years.

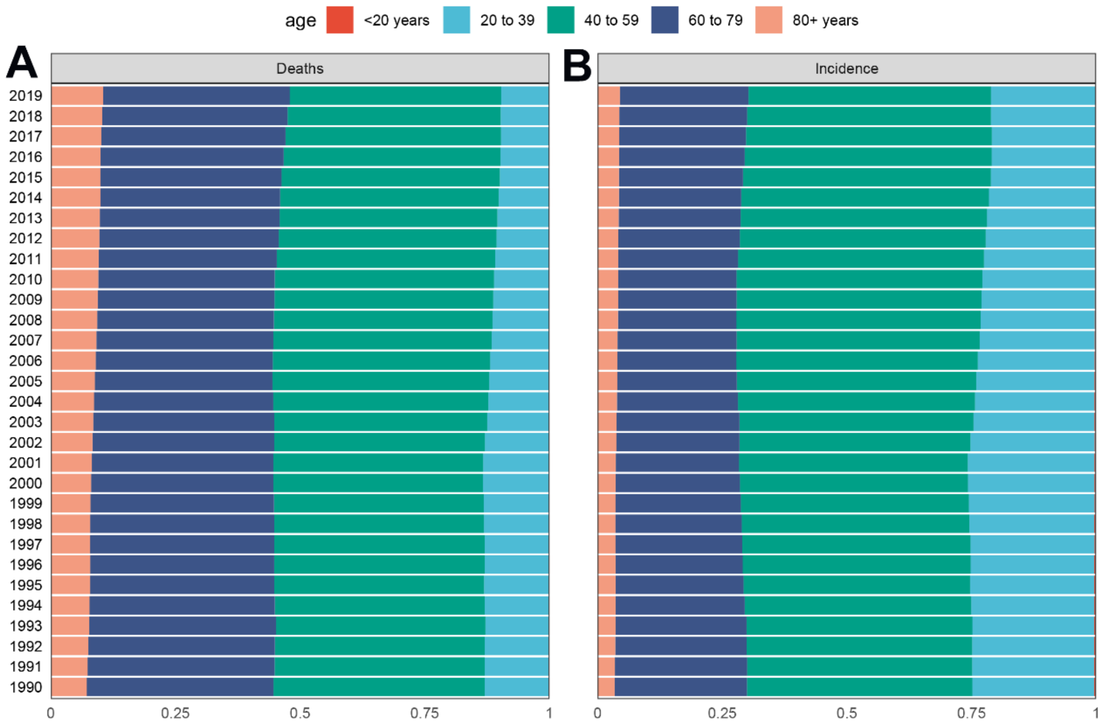

Supplementary Figure5. Distribution of different ages in cervical incidence(A), death(B), and DALYs(B) among different SDI quantiles from 1990 to 2019.

A

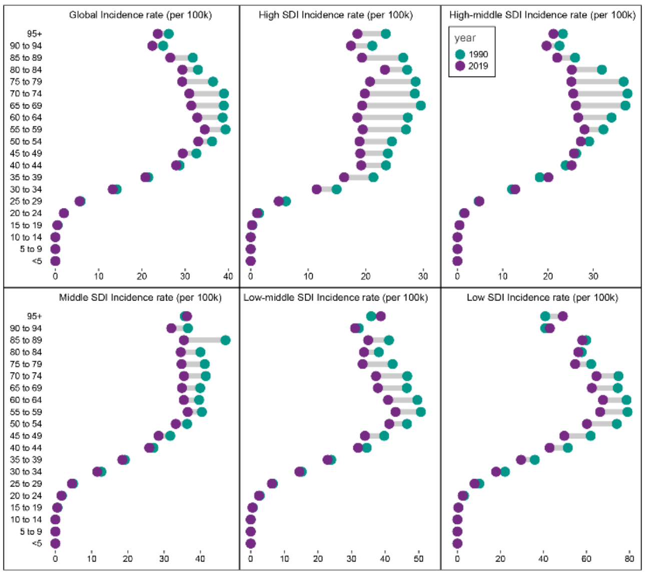
Global Incidence rate (per 100k)
High SDI Incidence rate (per 100k)
High-middle SDI Incidence rate (per 100k)
Middle SDI Incidence rate (per 100k)
Low-middle SDI Incidence rate (per 100k)
Low SDI Incidence rate (per 100k)
year
1990
2019
95+
90 to 94
85 to 89
80 to 84
75 to 79
70 to 74
65 to 69
60 to 64
55 to 59
50 to 54
45 to 49
40 to 44
35 to 39
30 to 34
25 to 29
20 to 24
15 to 19
10 to 14
5 to 9
<5
0 10 20 30 40
0 10 20 30
0 10 20 30
0 10 20 30 40
0 10 20 30 40 50
0 20 40 60 80

B

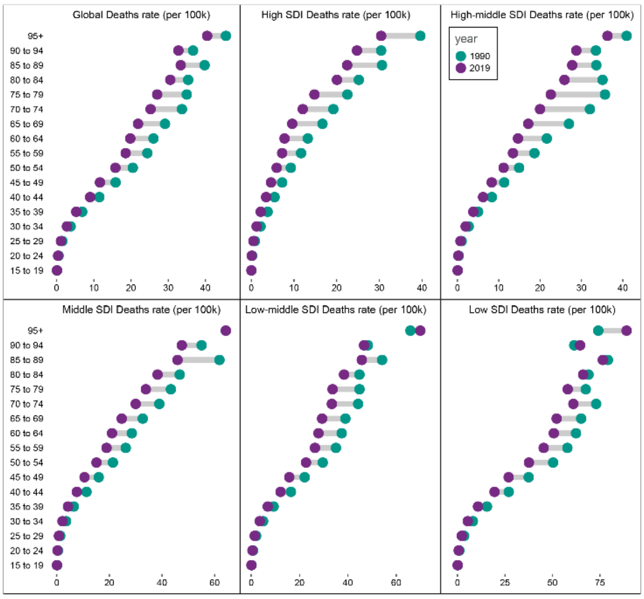
Global Deaths rate (per 100k)
High SDI Deaths rate (per 100k)
High-middle SDI Deaths rate (per 100k)
Middle SDI Deaths rate (per 100k)
Low-middle SDI Deaths rate (per 100k)
Low SDI Deaths rate (per 100k)
year
1990
2019
95+
90 to 94
85 to 89
80 to 84
75 to 79
70 to 74
65 to 69
60 to 64
55 to 59
50 to 54
45 to 49
40 to 44
35 to 39
30 to 34
25 to 29
20 to 24
15 to 19
0 10 20 30 40
0 10 20 30 40
0 10 20 30 40
0 20 40 60
0 20 40 60
0 25 50 75

C

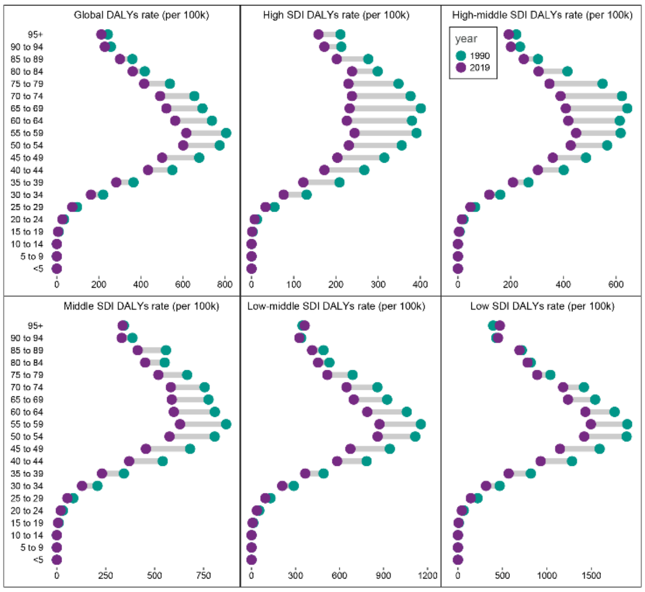
Global DALYs rate (per 100k)
High SDI DALYs rate (per 100k)
High-middle SDI DALYs rate (per 100k)
Middle SDI DALYs rate (per 100k)
Low-middle SDI DALYs rate (per 100k)
Low SDI DALYs rate (per 100k)
year
1990
2019
95+
90 to 94
85 to 89
80 to 84
75 to 79
70 to 74
65 to 69
60 to 64
55 to 59
50 to 54
45 to 49
40 to 44
35 to 39
30 to 34
25 to 29
20 to 24
15 to 19
10 to 14
5 to 9
<5
0 200 400 600 800
0 100 200 300 400
0 200 400 600
0 250 500 750
0 300 600 900 1200
0 500 1000 1500

Supplementary Figure6. The global EAPC of cervical cancer in 192 countries. (A). The EAPC of ASDR. (B). The EAPC of ASIR.(C) The EAPC of age-standardized DALY rate. ASDR, age-standardized death rate; ASIR, age-standardized incidence rate; EAPC, estimated annual percentage changes.

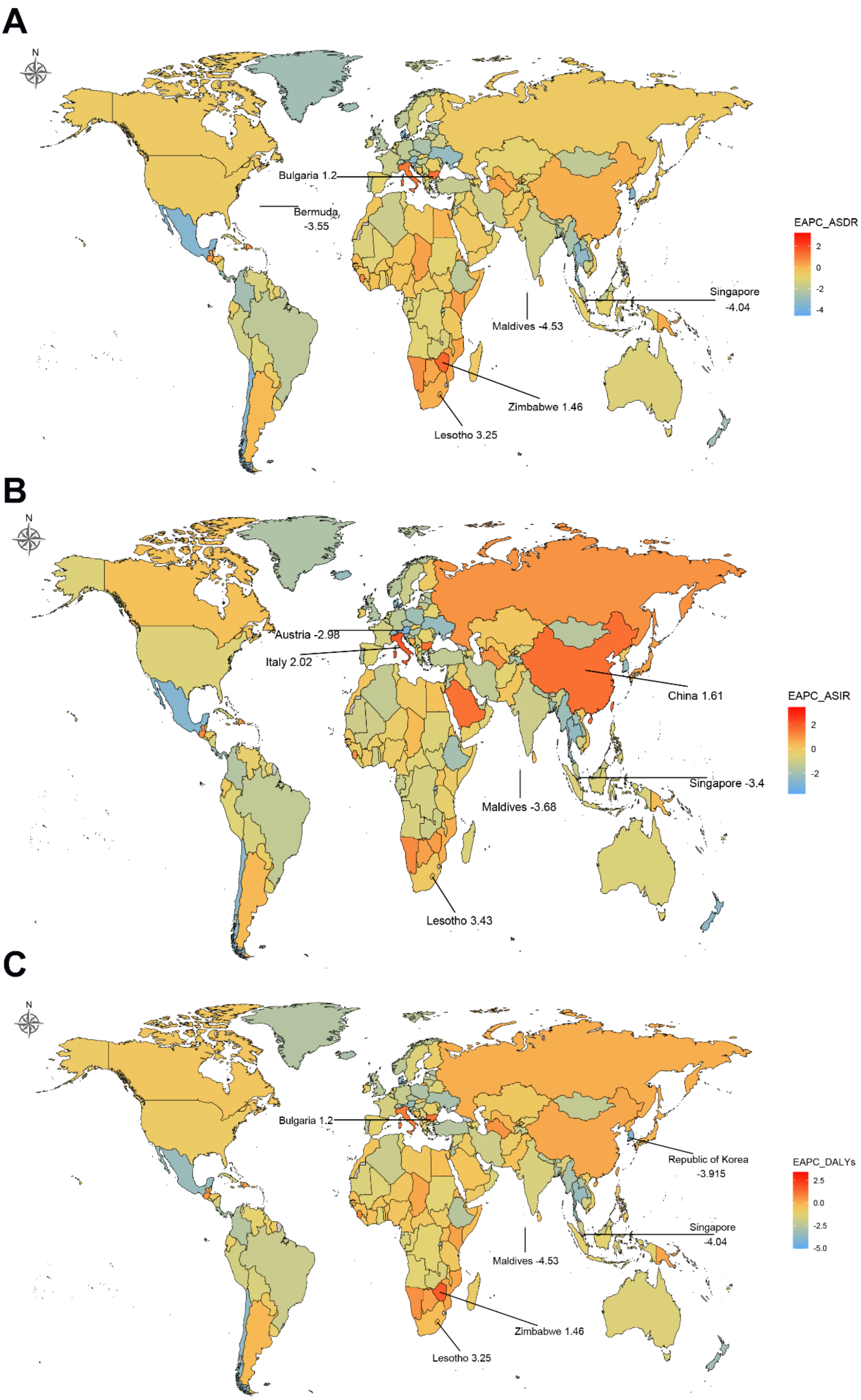
A
N
Bulgaria 1.2
Bermuda
-3.55
EAPC_ASDR
2
0
-2
-4
Singapore
-4.04
Maldives -4.53
Zimbabwe 1.46
Lesotho 3.25
B
N
Austria -2.98
Italy 2.02
China 1.61
EAPC_ASIR
2
0
-2
Singapore -3.4
Maldives -3.68
Lesotho 3.43
C
N
Bulgaria 1.2
Republic of Korea
-3.915
EAPC_DALYs
2.5
0.0
-2.5
-5.0
Singapore
-4.04
Maldives -4.53
Zimbabwe 1.46
Lesotho 3.25
